# Population Genomic Analyses Based on 1 Million SNPs in Commercial Egg Layers

**DOI:** 10.1371/journal.pone.0094509

**Published:** 2014-04-16

**Authors:** Mahmood Gholami, Malena Erbe, Christian Gärke, Rudolf Preisinger, Annett Weigend, Steffen Weigend, Henner Simianer

**Affiliations:** 1 Animal Breeding and Genetics Group, Department of Animal Sciences, Georg-August-University Göttingen, Göttingen, Germany; 2 LOHMANN Tierzucht GMBH, Cuxhaven, Germany; 3 Institute of Farm Animal Genetics (ING), Friedrich-Loeffler-Institut (FLI), Neustadt, Germany; Georg-August-Universitaet Goettingen, Germany

## Abstract

Identifying signatures of selection can provide valuable insight about the genes or genomic regions that are or have been under selective pressure, which can lead to a better understanding of genotype-phenotype relationships. A common strategy for selection signature detection is to compare samples from several populations and search for genomic regions with outstanding genetic differentiation. Wright's fixation index, F_ST_, is a useful index for evaluation of genetic differentiation between populations. The aim of this study was to detect selective signatures between different chicken groups based on SNP-wise F_ST_ calculation. A total of 96 individuals of three commercial layer breeds and 14 non-commercial fancy breeds were genotyped with three different 600K SNP-chips. After filtering a total of 1 million SNPs were available for F_ST_ calculation. Averages of F_ST_ values were calculated for overlapping windows. Comparisons of these were then conducted between commercial egg layers and non-commercial fancy breeds, as well as between white egg layers and brown egg layers. Comparing non-commercial and commercial breeds resulted in the detection of 630 selective signatures, while 656 selective signatures were detected in the comparison between the commercial egg-layer breeds. Annotation of selection signature regions revealed various genes corresponding to productions traits, for which layer breeds were selected. Among them were *NCOA1*, *SREBF2* and *RALGAPA1* associated with reproductive traits, broodiness and egg production. Furthermore, several of the detected genes were associated with growth and carcass traits, including *POMC*, *PRKAB2*, *SPP1*, *IGF2*, *CAPN1*, *TGFb2* and *IGFBP2*. Our approach demonstrates that including different populations with a specific breeding history can provide a unique opportunity for a better understanding of farm animal selection.

## Introduction

Charles Darwin suggested that the domestic chicken is descended from a single original species, the Red Jungle fowl (*Gallus gallus*), and that this happened in Southeast Asia nearly 10,000 years ago [1]. On the contrary, new studies suggested that the origin of domestic chickens lies in multiple origins in South and Southeast Asia [2], [3]. Selective breeding of chicken has been documented as early as Roman times. However, in contrast to current worldwide consumption of chicken meat and eggs as the major protein source [4] chicken may have been domesticated for cultural purposes such as religion, decoration, and cock fighting rather than for food production [5]. Strong selection of production traits started in the 20^th^ century when commercial breeds were selected for either egg-laying or meat production [6].

Strong selection has a direct effect on nucleotide diversity. Reduction or loss of nucleotide diversity at and near the selected locus caused by strong selection on desirable alleles is often referred to as genetic hitch-hiking or as a selective sweep [7]. Studying such signatures of selection can provide valuable insights about the genes or genomic regions that are or have been under selective pressure and hence can help in understanding important genotype-phenotype relationships. The discovery of a massive number of single nucleotide polymorphisms (SNPs) in the genomes of several species has enabled exploration of genome-wide signatures of selection via an assessment of variation in marker allele frequencies among populations [8]. A common strategy in this context is to compare samples from several populations, and look for genomic regions with outstanding genetic differentiation. Wright's fixation index, F_ST_, is a useful index of genetic differentiation between populations [9] and reflects the degree of differentiation between populations at any given locus, ranging from 0 (no differentiation) to 1 (fixed difference between populations). Negative or balancing selection tends to decrease F_ST_, whereas local positive selection tends to increase F_ST_ [10]. Genes responsible for phenotypic differences between populations are expected to show large allele frequency differences [11].

The growing genomic resources, the relatively rapid reproduction time and the existence of several inbred lines together with strong agricultural interest makes chicken an excellent model for studying the signatures of selection under artificial conditions [12]. A number of recent studies have investigated selection signatures in chicken either using sequence data or genotype data from low to medium density SNP chips. For example, Rubin *et al.* [13] studied the signatures of domestication and selective sweeps in various commercial broiler and layer lines using Next Generation Sequencing data from pooled DNA samples by searching for genomic regions with high degree of fixation of alleles. Johansson *et al.* [14] used a 60K SNP chip to study the genome wide effect of divergent selection between two chicken lines with a 9-fold difference in body weight. Elferink *et al.* [15] studied selective sweeps using the same method described by Rubin *et al.* [13] but carried out the study on a large number of chicken breeds (67 in total) using a 58K SNP chip.

In this study, 96 individuals from three commercial layer breeds and 14 non-commercial fancy breeds, including Red Jungle fowl (Cochin-Chinese) (*G. g. gallus*) and Red Jungle fowl (Burmese) (*G. g. spadiceus*), were genotyped with three different 600K SNP-chip from Affymetrix (with substantial proportion of overlapping SNPs between the three chips). This data set was produced during the validation of pre-screening arrays of the newly developed Axiom® Genome-Wide Chicken Genotyping Array [16]. Wright's fixation index, F_ST_, was used to study signatures of selection in the large dataset. The analysis of this large dataset provides an excellent basis for detecting selection signatures in the genomes of the chicken breeds under study and is unprecedented regarding the combination of number of genotyped individuals and marker density applied. This in turn can provide important information on the genomic regions which have been under selection and associated with specific layer traits.

## Material and Methods

### Animals, data collection and filtering

Two sets of samples, commercial egg layers and non-commercial fancy breeds (coded respectively LY and OG), were used for this study. The commercial individuals from Lohmann Tierzucht GmbH originated from three breeds: One commercial white egg layer breed based on White Leghorn (WL) with three separate lines and two brown egg layer breeds based on White Rock (WR) and Rhode Island Red (RIR), respectively, with two separate lines per breed. In each of these lines (seven in total) ten individuals were sampled and genotyped. The non-commercial fancy breeds consist of 26 individuals from 14 fancy breeds which were sampled within Synbreed project. The list of breeds with more details is presented in Table 1. OG breeds present a group of breeds that were not selected for commercial purpose such as egg or meat production. They consist of various breeds that were mainly selected for phonotypical traits such as feather color, feather style and comb style.

**Table 1:** Name, abbreviation, number of individuals and the egg color for each breed used in this study.

Breed	Abbreviation	# of lines	# of individuals	Egg color
White Leghorn	WL(1/2/3)	3	30(0♂,30♀)	White
Rhode Island Red	RIR(1/2)	2	20(2♂,18♀)	Brown
White Rock	WR(1/2)	2	20(2♂,18♀)	Brown
Asil	OG/Asil	1	2(0♂,2♀)	Brown
Brahma	OG/Brah	1	2(0♂,2♀)	Brown
Cochin	OG/Coch	1	2(0♂,2♀)	Brown
Fayoumi	OG/Fayo	1	2(0♂,2♀)	White
Gallus gallus gallus	OG/Ggal	1	2(0♂,2♀)	Brown
Gallus gallus spadiceus	OG/Gspa	1	2(0♂,2♀)	Brown
Green legged Partridge	OG/GreP	1	2(0♂,2♀)	White
Hungarian White Goedoelloe	OG/HunW	1	2(0♂,2♀)	Brown
Jaerhoens	OG/Jaer	1	2(0♂,2♀)	White
Malay	OG/Mala	1	2(0♂,2♀)	Brown
Marans	OG/Mara	1	2(0♂,2♀)	Brown
Orlov	OG/Orlo	1	2(0♂,2♀)	White
Paduaner	OG/Padu	1	1(0♂,1♀)	White
Transylvanian Naked Neck	OG/Tran	1	1(0♂,1♀)	Brown

DNA was isolated using a phenol/chloroform method for the DNA isolation [17] from whole blood collected from the wing vein using EDTA as anticoagulant. DNA quality and concentration of each sample was calculated and equal amounts of DNA were used for genotyping on three Affymetrix 600K SNP arrays using the Affymetrix® GeneTitan® system according to the procedure described by Affymetrix [18]. Data is available from the authors upon request.

This study was carried out in strict accordance with the German Animal Welfare regulations. The blood taking protocol was approved by the Committee of Animal Welfare at the Institute of Farm Animal Genetics of the Friedriech-Loeffler-Institut. Blood sampling was also notified to the Lower Saxonian authorities according to § 8a para. 1 of the German Animal Welfare Act. The blood takings were registered at the Lower Saxony State Office for Consumer Protection and Food Safety (Registration Number 33.9-42502-05-10A064).

Overlapping SNPs between the three 600K SNP arrays were removed and a total of 1,139,073 SNPs remained. To avoid imputation error in further analyses and due to the high amount of SNP and good coverage of the genome, 148,712 SNPs with at least one missing value were removed. Next the included SNPs were filtered for minor allele frequencies lower than 5% (74,202 were removed) in order to avoid genotyping errors, this approach was suggested by the data provider. The SNPs were located on autosomal chromosomes (1–28), one sex chromosome (Z), and two linkage groups, LGE22C19W28_E50C23 and LGE64, which were named Chr40 and Chr41, respectively. A total of 916,159 SNPs remained after filtering (throughout this paper, 916,159 is referred to as 1M SNPs). The entire filtering process was done by using the software PLINK (http://pngu.mgh.harvard.edu/purcell/plink/) [19].

### Population structure analysis

Two methods were used in order to retrieve the structure of the studied samples; principal component analysis (PCA) using the R package ADEGENET [20], [21] and maximum likelihood estimation of individual ancestries using ADMIXTURE software with several null hypotheses [22].

### F_ST_ calculation and permutation test

To identify the regions under selection, Wright's F_ST_ [9] was calculated for all pairwise combinations of breeds and average F_ST_ values were calculated for overlapping windows along each chromosome. Each window consisted of 40 SNPs with an overlap of 20 SNPs with the next window. Average window size was 20,554 bp with a minimum of 2,029 bp and a maximum of 6,633,801 bp.

To assess distribution of the F_ST_ values we conducted a permutation test with 100 replications. For each replicate the individuals were randomly assigned to one of two groups, then F_ST_ was calculated for each SNP and averaged for the same windows as with the non-permuted data. The maximum and minimum F_ST_ value then was stored for each replicate.

### Signatures of selection

**Figure 1:**
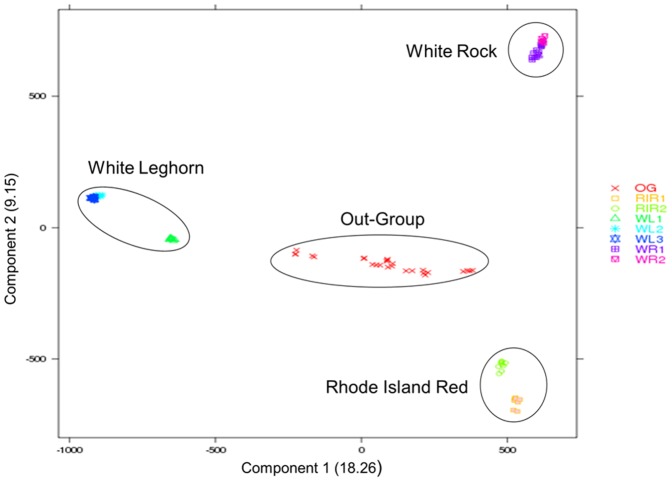
PCA analysis for all the 96 individuals with 1 million SNPs.

**Figure 2:**
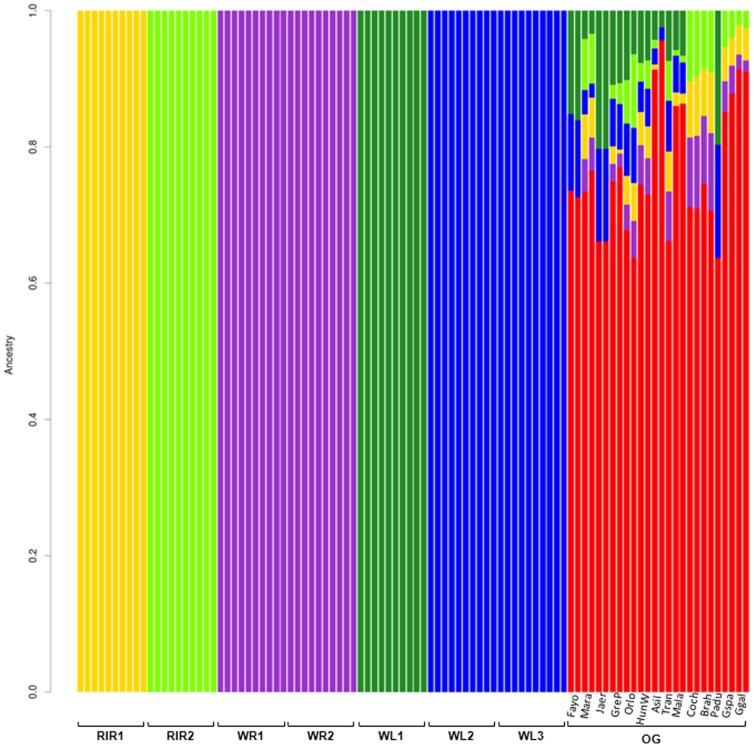
Result of ADMIXTURE structural analysis with null hypothesis of six breeds. Two rightmost individuals in OG are Gallus gallus gallus, and the third and fourth last individuals are Gallus gallus spadiceus.

According to the PCA and ADMIXTURE structural analysis (Figure 1 and Figure 2, respectively), breeds were arranged in six different groups; the two White Rock lines were pooled together (WR, n = 20), each of the Rhode Island Red lines remained in one group (RIR1, n = 10 and RIR2, n = 10), White Leghorn line one was kept as one group (WL1, n = 10), line two and line three from White Leghorn were pooled together (WL2&3, n = 20), and all the non-commercial chicken breeds were pooled in one group (OG, n = 26).

Two sets of comparisons were made in this study in order to detect selection signatures. First, a comparison between commercial egg layers and the out-group (LY vs. OG) was carried out. For this comparison, F_ST_ values between the out-group and each of the commercial groups (RIR1, RIR2, WR, WL1 and WL2&3) were calculated for each SNP in the window and averaged. Second a comparison between white egg layers and brown egg layers (WL vs. BL) was conducted. In this case, the average of F_ST_ values between the white egg layers (WL1 and WL2&3) and the brown egg layers (RIR1, RIR2 and WR) in each window was calculated.

Next, based on the genome-wide distribution of F_ST_, a threshold cutting of the upper and lower 1% was used for the definition of extreme values. To compensate for the higher average F_ST_ on sex-chromosome Z compared to the autosomes, the thresholds for chromosome Z were determined separately, by cutting of the upper and lower 1% of the F_ST_ distribution on chromosome Z [23].

### Annotation

The regions with extreme F_ST_ values can be considered as good candidates for selective sweeps. For each comparison all the extreme windows (the upper or lower 1%) that were within 500 kb of each other were grouped to form a set of joined windows. For all joined windows gene annotation and pathway annotation was completed. Gene annotations were done with the biomaRt R package [24] based on Ensembl data base [25]. For pathway annotation KEGG database [26] was used. Fisher exact test was run for gene enrichment analysis for all annotated genes using DAVID (The Database for Annotation, Visualization and Integrated Discovery) [27], [28]. We assumed pathways and gene ontologies with p≤0.05 as being under selection.

## Results and Discussion

Components one and two of the PCA analysis with 1M SNPs, jointly accounting for 27.4 per cent of the total variance, are plotted in Figure 1. The commercial white egg-layer breeds were separated by component 1 from brown egg-layers. In addition, two brown egg-layer breeds (RIR and WR) were separated from each other by component 2. The outgroup is rather diverse and stays in the center of the distribution. As expected from the Lohmann breeding program, line two and line three of White Leghorns, and both lines in White Rock clustered together, respectively.

Additionally, based on the cross validation test of admixture with all the commercial breeds, maximum likelihood estimation of the individual ancestries under the null hypothesis of six populations was run for 1M SNPs. The result is shown in Figure 2. These analyses are largely in agreement with the expected historical origin of the breeds [5] and the result of the PCA. Admixture analysis clustered OG breeds as one group; however there was an admixture between different breeds in OG with layer breeds. Interestingly, there is no admixture between White Leghorns and ancestral chicken breeds (*Gallus gallus* and *Gallus spadiceus*).

Based on these results, individuals were arranged in six different breed groups of WL1, WL2&3, WR, RIR1, RIR2 and OG.

Average F_ST_ within brown layers (RIR vs. WR, 0.18) was lower than the average F_ST_ value between white layers and brown layers (RIR vs. WL (0.24) and WR vs. WL (0.26)) (shown in Table 2), which shows that the similarity within the brown layers is higher than between white layers and brown layers, as it is expected. The average F_ST_ values along with the standard deviation for all group comparisons are shown in Table 3. In general, F_ST_ values between the out-group and commercial layer breeds are lower than the F_ST_ values between two commercial layer breeds, which is due to the fact that the allele frequency spectrum in commercial layers follows a U-shaped distribution while in the out-group it follows approximately a uniform distribution (results are not shown). F_ST_ values between lines of breeds are always lower than between breeds, which show the similarity within breeds is much higher than between breeds.

**Table 2:** Average F_ST_ values with standard deviation between different breeds.

	WL	WR
**RIR**	0.2419(±0.25)	0.1768(±0.20)
**WR**	0.2641(±0.27)	

**Table 3:** Average F_ST_ values with standard deviation over all SNPs for all compression.

	WL2and3	RIR1	RIR2	WR	OG
**WL1**	0.1543(±0.21)	0.2653(±0.31)	0.2524(±0.30)	0.2382(±0.29)	0.1184(±0.14)
**WL2and3**		0.2715(±0.32)	0.2590(±0.30)	0.2567(±0.30)	0.1570(±0.17)
**RIR1**			0.1148(±0.17)	0.1662(±0.23)	0.1006(±0.13)
**RIR2**				0.1523(±0.24)	0.0904(±0.11)
**WR**					0.1155(±0.13)

The permutation test showed that the F_ST_ distribution under randomization is much lower than the observed distribution of F_ST_ (results not shown). In all cases the minimum F_ST_ value obtained from the permuted data was close to zero and the maximum was around 0.3, which corresponds to a threshold 10 times lower than the threshold that we used, and is not helpful for the derivation of empirical threshold values.

**Figure 3:**
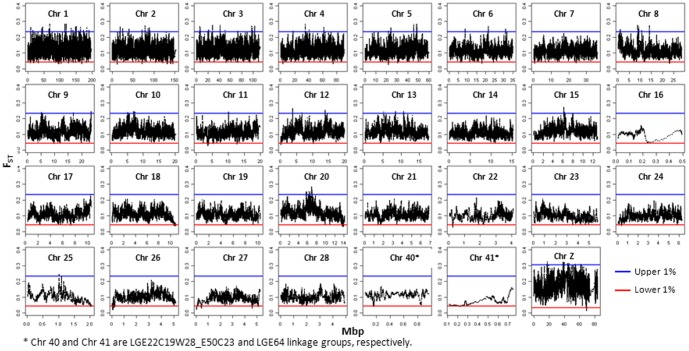
FST-values of overlapping windows for comparison between commercial layers and out-group. Red (blue) line indicates the upper (lower) 1% of FST distribution.

**Figure 4:**
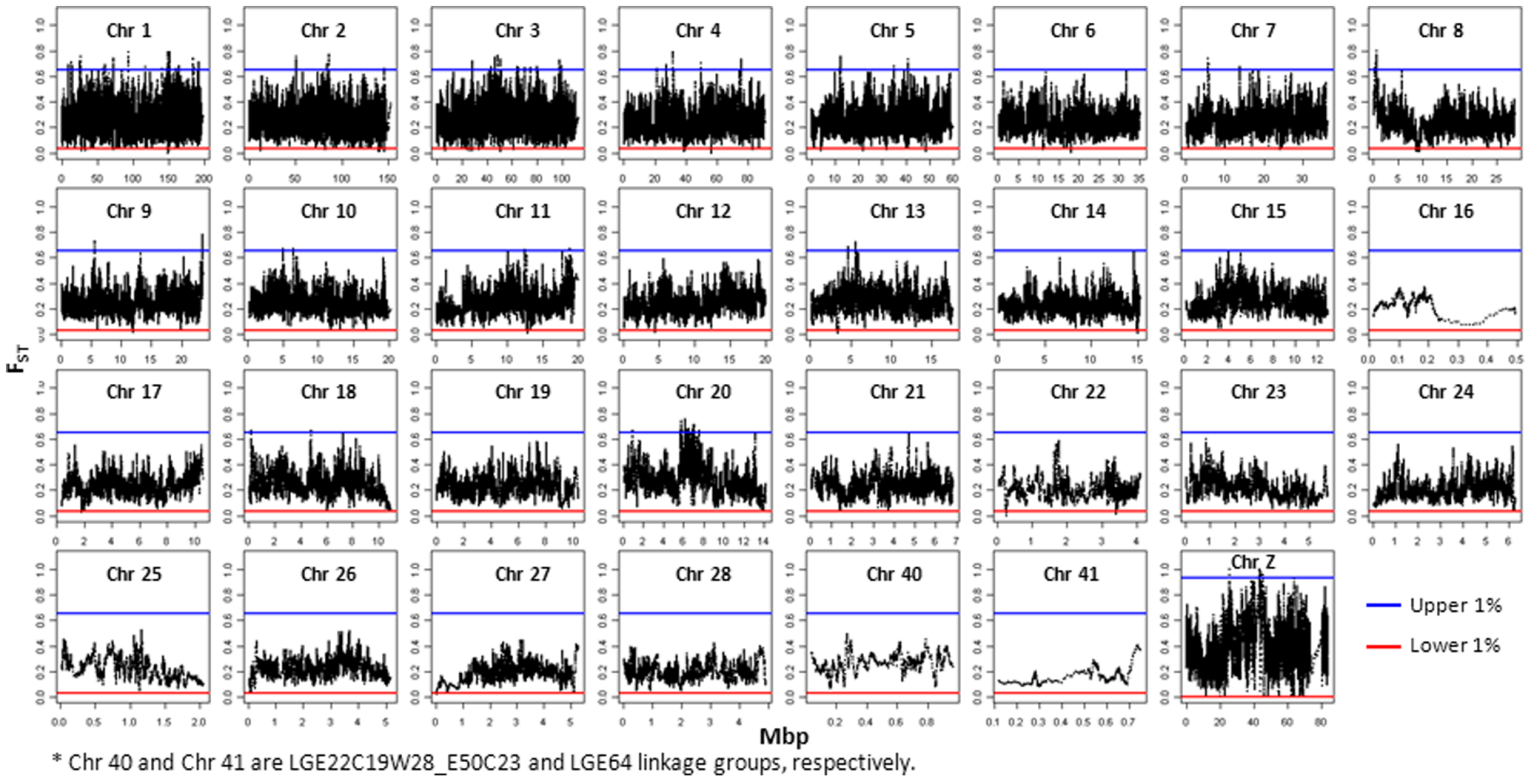
FST-values of overlapping windows for comparison between brown layers and white layers. Red (blue line) indicates the upper (lower) 1% of FST distribution.

Based on F_ST_ values averaged in overlapping windows a total of 656 selective signatures (321 and 335 regions for the upper and lower 1% F_ST_ distribution, respectively) were detected when comparing commercial egg-layer breeds. In the comparison between non-commercial and commercial breeds, a total of 630 selective signatures (322 and 308 regions for the upper and lower 1% F_ST_ distribution respectively) were detected. The genome-wide distribution of F_ST_ values obtained with the comparison LY vs. OG and WL vs. BL are depicted in Figures 3 and 4, respectively.

The overlapping windows method was used for two reasons: to reduce the noisiness of single-locus statistics by combining data from several adjacent markers, and to avoid the risk of passing over genomic gaps. As Qanbari *et al.* [29] suggested, the use of overlapping windows has a higher power of detecting selective sweeps compared to sliding windows. In this work, defining a window size of 40 SNPs was a subjective decision, but it was motivated by previous studies [13], [30] and the requirement of having sufficient coverage all over the genome. SNPs on each of the three Affymetrix 600K SNP arrays are distributed equally with respect to the genetic distance; this explains the large difference of window size based on bp. The outlier approach is an effective method for identifying the genes under selection lacking known phenotypes [31]. However, as Akey [32] explained, an outlier signal is not necessarily synonymous with regions being under selection.

Many of the detected outliers could be considered false positives. This might be the case because the F_ST_ calculations assume that the populations have the same effective size and were derived independently from the same ancestral population. The error caused by this assumption is similar to well-known effects of cryptic structure in genome-wide association studies [33].

Regions with F_ST_ values in the lower tail of the distribution are of interest for comparison of commercial breeds, which have been selected for very similar traits but starting from a very diverse genetic background, especially so for white and brown layers. In contrast, F_ST_ values in the upper tail of the distribution are of interest since they may display regions under selection for different breeding goals such as egg shell color. For comparing commercial breeds with the non-commercial breeds, the regions with F_ST_ values in the upper tail of the distribution are relevant because of the large contrast in breeding goals between these groups while the regions with F_ST_ values in the lower tail of the distribution might show regions that have been selected naturally or artificially before the intense selection on laying performance in commercial breeds.

Annotation was carried out for all regions with extreme F_ST_ values, i.e. potential selective sweeps. The lists of genes for selective sweeps are available in the supplementary tables (Table S1, S2, S3 and S4). In general, the annotation list is enriched with genes of biological interest involved in various pathways such as cellular amino acid catabolic process (p = 0.012), regulation of growth (p = 0.012), calcium ion binding (p = 0.033), B cell activation (p = 0.031), immune system development (p = 0.034) and post-embryonic development (p = 0.035), all of which could be related to production traits indirectly. The lists of pathways and gene ontologies under selection are available in the supplementary tables (Table S5, S6, S7 and S8). In both comparisons (LY vs. OG and WL vs. BL), we were able to identify several genes related to the breeding goals of egg-layer chickens, such as the age at sexual maturity, laying rate, body weight, and feed conversion [34] (Table 4).

**Table 4:** Genes associated to productive traits in both comparisons. ≠ symbol stands for difference between two group and  =  symbol stand for similarity between two groups.

Gene	Chr	Function	Comparison
**SREBF2**	1	Involved in the rapid growth stages of follicle development.	B≠W
**POU1F1**	1	Associated with growth performance in chicken.	L = G
**CST3**	3	Involve in calcium release into the medium.	B = W
**TGFB2**	3	Significantly associated with chicken growth traits and not associated with any reproduction traits	L≠G,B≠W
**CAPN1**	3	Associated with meat quality traits in chicken.	B≠W
**NCOA1**	3	Associated with total egg production at (age 300 day) and age at first egg	L≠G
**POMC**	3	Associated with feed conversion and body weight in commercial broiler	L≠G
**SPP1**	4	Associated with 5-week body weight and quality of egg shells in laying hens	L≠G
**IGFII**	5	Influencing growth and carcass traits.	B≠W
**RALGAPA1**	5	Associated with reproductive traits and broodiness.	B≠W
**IGFBP2**	7	Associated with body composition, body weight, and affects fatness traits in chickens	L≠G, B = W
**PRKAB2**	8	Associated with live-weight, carcass-weight, leg-muscle-weight and abdomen-fat-weight	L≠G
**CCT6A**	19	Associated with sexual maturity in hens.	L = G
**IL 19**	26	Assoiciated with responses to intracellular poultry pathogens like bacteria and protozoa.	L = G
**AMH**	28	Expression is significantly greater in broiler breeder hens as compared with laying hens.	L = G
**SLC45A2**	Z	Inhibitor of expression of red pheomelanin in Silver chickens.	L = G

B and W stand for comparison between brown and white egg layers and L and G stand for comparison between commercial layers and out-group.

Many genes were identified in selective sweep regions in the comparison between brown and white layers. *TGFb2*, *CAPN1* and *IGF2* were all located in regions that were different between brown and white layers. *TGFb2* (transforming growth factor, beta 2) is significantly associated with chicken growth traits and is not associated with any reproduction traits [35]. *TGFb2* is expressed 4-fold greater in broiler compared with layer hens at 15 weeks of age [36]. *CAPN1* is associated with meat quality [37], [38]. *IGF2* (insulin-like growth factor 2), which is believed to be a major fetal growth factor in contrast to insulin-like growth factor 1 [39], has a great influence on growth and carcass traits in chicken [40]. The presence of genes associated with meat quality and production in regions that were different between brown and white layers reflects the fact that brown egg-layers were originally a dual-purpose breed. Specifically, brown layers were bred for meat production as well as egg-production, whereas white egg layers were bred only for egg production [5]. *SREBF2* and *RALGAPA1*, which are both associated with reproductive traits and broodiness [41], [42], were also located in the regions with high contrast between the two layer breeds. This can indicate that different regions were selected for reproductive traits in the different egg-layer breeds.

In the comparison of commercial-layers and out-group, *NCOA1*, which corresponds to the total egg production (at age 300 days) and age at first egg [43], along with *SPP1*, which is associated with 5-week body weight and quality of egg shells in laying hens [44], were located in the regions that were different between commercial-layers and out-group chicken. This may reflect the intense selection of the regions associated with egg production and quality traits in laying breeds. *PRKAB2*, *POMC* and *TGFb2* which are associated with live-weight, carcass-weight, leg-muscle-weight, abdomen-fat-weight and feed conversion, were also located in the regions that differ between commercial-layers and out-group chicken [35], [45], [46]. This may be due to the existence of brown egg layers with a dual purpose ancestral background in the commercial-layers group under study.

*IGFBP2*, which inhibits or stimulates the growth promoting effects of the *IGFs* [47],is associated with body composition, body weight and affects fatness traits in chickens [48], [49], was identified both in the similarity between the two layer breeds (white layers and brown layers) and in the difference between the layers and the out-group. This indicates positive selection of this gene in both groups of layer breeds, although they have different genetic background and they have been selected separately.

Several further regions were identified as selective signatures in the comparison between commercial lines and the out-group. These regions mainly corresponded to primary genes such as *CCT6A* and *IL19*. *CCT6A* is a gene associated with sexual maturity in hens [50], and *IL19* plays an important role in responses to intracellular poultry pathogens like bacteria and protozoa [51]. *POU1F1* and *AMH*, which are both genes related to the growth performance in broiler chickens [52]–[54], were identified in regions that show similarity between the out-group and commercial layers.

In this study, we have identified more regions as putative selective sweeps compared to previously reported data by Rubin *et al.* [13] and Elferink *et al.* [15]. However, several of these regions were not associated with any genes related to production traits. This could be due to insufficient knowledge about these regions or it could also reflect false positives caused by genetic drift following the separation of the breeds [55]. Although, we have not annotated selection signatures reported in other studies [13], [15], our results agree with previously reported findings with respect to identified homologs of the same genes. For instance, *IGF2* is a homolog of *IGF1* which was identified in two studies [13], [15]. We also identified *POU1F1* which binds to and transactivates promoters of growth hormone (GH) and thyroid-stimulating hormone chain (*TSHB*)-encoding genes [56], which were identified by Rubin and Elferink [13], [15]. Another reason that the detected genes are different from the previous studies [13], [15] could be that our study was based on layer breeds while other works included broiler breeds.

In conclusion we were able to identify several putative selective signature regions with genes corresponding to the productions traits layer breeds were selected for. These identified regions are good candidates for further studies. It was demonstrated that layers with a specific breeding history, which has led to animals with a very similar performance profile coming from a much differentiated genetic background, provide a unique opportunity for a better understanding of farm animal selection.

## Supporting Information

Table S1 List of genes in lower 1% distribution of the comparision of commercial layers and out-group.
(PDF)

Table S2 List of genes in of upper 1% FST distribution in comparision of commercial layers and out-group.
(PDF)

Table S3 List of genes in lower 1% FST distribution in comparision of brown layers and white layers.
(PDF)

Table S4 List of genes in upper 1% FST distribution in comparision of brown layers and white layers.
(PDF)

Table S5 List of pathways and gene ontologies in lower 1% distribution of the comparison of commercial layers and out-group.
(PDF)

Table S6 List of pathways and gene ontologies in upper 1% distribution of the comparison of commercial layers and out-group.
(PDF)

Table S7 List of pathways and gene ontologies in lower 1% FST distribution in comparison of brown layers and white layers.
(PDF)

Table S8 List of pathways and gene ontologies in upper1% FST distribution in comparison of brown layers and white layers.
(PDF)
