# Supplementary material for: Population Genomic Analyses Based on 1 Million SNPs in Commercial Egg Layers
Source: PLoS One. 2014 Apr 16;9(4):e94509. doi: 10.1371/journal.pone.0094509 (PMC3989219; doi:10.1371/journal.pone.0094509)
Supplement: Table S1 — List of genes in lower 1% distribution of the comparision of commercial layers and out-group. (PDF) [file pone.0094509.s001.pdf]

Table S1. List of genes in lower 1% distribution of the comparision of commercial layers and out-group.

Chr	Start	End	Description	Fst
1	1361642	1822074	exocyst complex component 4	0.043
1	5715214	5839991	CUGBP Elav-like family member 2	0.053
1	5900537	6010810	USP6 N-terminal-like protein	0.053
1	6030820	6035332	enoyl-CoA hydratase domain-containing protein 3, mitochondrial	0.053
1	6081854	6134287	UPF2 regulator of nonsense transcripts homolog (yeast)	0.053
1	6143197	6160464	dehydrogenase E1 and transketolase domain containing 1	0.053
1	6167427	6183132	Sec61 alpha 2 subunit (S. cerevisiae)	0.053
1	6184287	6193751	nudix (nucleoside diphosphate linked moiety X)-type motif 5	0.053
1	8177104	8318907	Semaphorin-3D	0.036
1	8522175	8850302	semaphorin-3A	[illegible]
1	9561909	9881531	piccolo presynaptic cytomatrix protein	0.042
1	13626518	13719427	SRSF protein kinase 2	0.057
1	16987071	17299542	family with sequence similarity 19 (chemokine (C-C motif)-like), member A5	0.050
1	18537716	18596571	bromodomain-containing protein 1	0.036
1	18646102	18656551	alpha-1,6-mannosyltransferase ALG12 precursor	0.036
1	18657758	18668817	cysteine-rich with EGF-like domain protein 2 precursor	0.036
1	21513823	21575759	protection of telomeres protein 1	0.050
1	21614383	21629956	probable G-protein coupled receptor 37 precursor	0.050
1	32863934	32927887	ubiquitin carboxyl-terminal hydrolase 15 isoform 1	0.036
1	32942077	33016611	protein MON2 homolog	0.036
1	52867138	53186826	synapsin III	0.057
1	53058721	53091132	Metalloproteinase inhibitor 3	0.057
1	56510668	56527880	SVOP-like	0.055
1	56529994	56569101	tripartite motif containing 24	0.055
1	67177978	67190068	L-lactate dehydrogenase B chain	0.049
1	67311818	67331462	cancer susceptibility candidate 1	0.049
1	81373015	81656000	Limbic system-associated membrane protein	0.042

1	81680485	81734254	growth associated protein 43	0.042
1	87611977	87655399	poliovirus receptor-related 3	0.039
1	87804275	87808822	phosphatidylinositol-specific phospholipase C, X domain containing 2	0.039
1	87878151	87933199	pleckstrin homology-like domain, family B, member 2	0.039
1	88084658	88092055	uncharacterized protein LOC418424 precursor	0.039
1	88543354	88765491	ephrin type-A receptor 3 precursor	0.049
1	90911605	90946738	protein kinase substrate MK2S4	0.052
1	90955206	90960077	Protein CREG1	0.052
1	91027333	91088674	POU domain, class 2, transcription factor 1	0.052
1	91759587	91767822	acid phosphatase 6, lysophosphatidic	0.047
1	91781764	91800664	gap junction alpha-5 protein	0.047
1	91850790	91873866	Golgi pH regulator	0.047
1	91872169	91878587	PDZ domain containing 1	0.047
1	91839809	91841011	gap junction alpha-8 protein	0.047
1	94474405	94635350	glucan (1,4-alpha-), branching enzyme 1	0.033
1	95642548	95948471	roundabout, axon guidance receptor, homolog 1 (Drosophila)	0.033
1	94975844	94976132	Metazoan signal recognition particle RNA	0.033
1	96554624	96554779	TUC338	0.033
1	94992672	94992743	U6 spliceosomal RNA	0.033
1	97936529	98030131	ubiquitin specific peptidase 25	0.031
1	98638187	98668892	coxsackie virus and adenovirus receptor	0.031
1	98700854	98720026	protein EURL	0.031
1	98864912	98893667	chondrolectin	0.031
1	97651374	97654841	nuclear receptor interacting protein 1	0.031
1	98348216	98348323	gga-let-7c	0.031
1	103284519	103312107	E3 ubiquitin-protein ligase listerin	0.052
1	103318664	103336698	ubiquitin specific peptidase 16	0.052
1	103338188	103348898	T-complex protein 1 subunit theta	0.052
1	103488818	103564194	glutamate receptor, ionotropic, kainate 1	0.052
1	103227959	103228104	TUC338	0.052

1	104404065	104411097	uncharacterized protein C21orf59 homolog	0.034
1	104478101	104503622	PAX3 and PAX7 binding protein 1	0.034
1	106011589	106039528	MORC family CW-type zinc finger 3	0.055
1	106039840	106057388	chromatin assembly factor 1 subunit B	0.055
1	111360104	111377529	ATP-dependent RNA helicase DDX3X	0.053
1	111416662	111489576	Ubiquitin carboxyl-terminal hydrolase	0.053
1	117286493	117477666	DNA polymerase	0.055
1	117443614	117443744	Small Cajal body specific RNA 24	0.055
1	119398188	119620052	SH3 domain-containing kinase-binding protein 1	0.050
1	119632060	119703945	mitogen-activated protein kinase kinase kinase 15	0.050
1	125536411	125555323	patatin-like phospholipase domain-containing protein 4	0.057
1	125766037	125867934	steroid sulfatase (microsomal), isozyme S	0.057
1	127508430	127539291	protein kinase, X-linked	0.036
1	127679910	127698770	matrix-remodelling associated 5	0.036
1	127805905	127818492	arylsulfatase H precursor	0.036
1	127871298	127881303	glycogenin 2	0.036
1	127916919	127941663	CD99 antigen precursor	0.036
1	128381550	128389045	hydroxyindole O-methyltransferase	0.036
1	128396788	128405095	splicing factor, arginine/serine-rich 17A	0.036
1	128441122	128467057	P2Y purinoceptor 8	0.036
1	128639645	128657470	cytokine receptor-like factor 2	0.036
1	156898969	157114305	kelch-like family member 1	0.050
1	158732522	158732579	Small nucleolar RNA SNORD63	0.049
1	169373629	169393173	serpin peptidase inhibitor, clade E (nexin, plasminogen activator inhibitor type 1), member 3	0.032
1	174734826	174993334	microtubule associated tumor suppressor candidate 2	0.051
1	182715565	183138508	contactin-5 precursor	0.040
1	186948732	187052406	NADPH oxidase 4	0.056
2	4706213	4774487	mitogen-activated protein kinase kinase kinase 3-like	0.056
2	6247476	6461068	5'-AMP-activated protein kinase subunit gamma-2	0.049
2	27518562	27854244	diacylglycerol kinase, beta 90kDa	0.047

2	33497042	33670877	juxtaposed with another zinc finger protein 1	0.055
2	34829807	34925176	raftlin	0.053
2	38757266	38807866	leucine rich repeat containing 3B	0.052
2	50031906	50327049	POU class 6 homeobox 2	0.041
2	50214117	50214294	TUC338	0.041
2	56697424	56780391	nuclear factor of activated T-cells, cytoplasmic, calcineurin-dependent 1	0.050
2	73536943	73537003	Small nucleolar RNA R11/Z151	0.044
2	77784558	78033401	catenin (cadherin-associated protein), delta 2	0.050
2	92484359	92551804	neuropilin (NRP) and tolloid (TLL)-like 1	0.049
2	111761499	111804172	neutral sphingomyelinase (N-SMase) activation associated factor	0.042
2	111851189	112072073	thymocyte selection-associated high mobility group box protein TOX	0.042
2	112517303	112558232	Ras-related protein Rab-2A	0.042
2	112612964	112699976	Chromodomain-helicase-DNA-binding protein 7	0.042
2	113543309	113551391	YTH domain family protein 3	0.046
2	113888475	113888659	TUC338	0.046
2	117914310	117920391	ganglioside induced differentiation associated protein 1	0.045
2	119025058	119150906	Zinc finger homeobox protein 4	0.045
2	119193465	119216508	peroxin 2	0.045
2	127436600	127569389	serine/threonine kinase 3	0.054
2	128591603	128652059	grainyhead-like 2 (Drosophila)	0.050
2	130946459	131114196	angiopoietin-1 precursor	0.031
2	131277175	131326213	R-spondin 2	0.031
2	131405148	131425618	eukaryotic translation initiation factor 3 subunit E	0.031
2	131497565	131532447	tetratricopeptide repeat protein 35	0.031
2	131690687	131704507	thyrotropin-releasing hormone receptor	0.031
2	131729098	131754322	NudC domain containing 1	0.031
2	131770048	131789265	receptor-binding cancer antigen expressed on SiSo cells	0.031
2	131790739	131821463	syntabulin (syntaxin-interacting)	0.031
2	132812344	133381395	CUB and Sushi multiple domains 3	0.031
2	140145617	140283188	ArfGAP with SH3 domain, ankyrin repeat and PH domain 1	0.033

2	140874788	140936443	EFR3 homolog A (S. cerevisiae)	0.033
2	140938828	140957795	Otoconin	0.033
2	140961259	140974304	HERV-H LTR-associating 1	0.033
2	140989921	141021182	potassium voltage-gated channel, KQT-like subfamily, member 3	0.033
2	140759405	140759511	U6 spliceosomal RNA	0.033
2	144142255	144374426	collagen, type XXII, alpha 1 precursor	0.046
3	6450555	7094776	neurexin-1-alpha isoform 1 precursor	0.038
3	9652846	9689626	aftiphilin	0.055
3	9699424	9700374	SERTA domain-containing protein 2	0.055
3	10359052	10449924	Meis homeobox 1	0.044
3	10819837	10824742	Ewing tumor-associated antigen 1	0.044
3	11105742	11120517	nuclear nucleic acid-binding protein C1D	0.044
3	11131168	11137396	WD repeat-containing protein 92	0.044
3	11137452	11140992	RNA-binding protein PNO1	0.044
3	11142465	11175965	calcineurin subunit B type 1	0.044
3	11215025	11230471	pleckstrin	0.044
3	11272177	11281262	prokineticin receptor 2	0.044
3	11363004	11381764	filensin	0.044
3	11382922	11480137	proprotein convertase subtilisin/kexin type 2	0.044
3	11635647	11793882	serine/threonine-protein kinase MRCK alpha	0.044
3	31632109	31793597	cysteine-rich motor neuron 1 protein precursor	0.041
3	36498791	36661068	ryanodine receptor 2 (cardiac)	0.052
3	48447032	48720659	spectrin repeat containing, nuclear envelope 1	0.057
3	48776284	48795822	VIP peptides isoform 2 preproprotein	0.057
3	54542844	54704551	phosphodiesterase 7B	0.053
3	54859271	54941536	Abelson helper integration site 1	0.053
3	54967896	54990656	transcriptional activator Myb	0.053
3	60315841	60432093	triadin	0.045
3	60548727	60598139	clavesin 2	0.045
3	60674294	60677648	Fatty acid-binding protein, brain	0.045

3	60687661	60743857	protein kinase (cAMP-dependent, catalytic) inhibitor beta	0.045
3	60764156	60778623	serine incorporator 1 precursor	0.045
3	60779761	60800976	Heat shock factor protein 2	0.045
3	72576579	72595135	mannosidase, endo-alpha	0.043
3	72615240	72615343	U6 spliceosomal RNA	0.043
3	80928945	80936582	protein MTO1 homolog, mitochondrial	0.053
3	80937537	80943626	Mab-21 domain containing 1	0.053
3	86766922	86825286	bone morphogenetic protein 5 precursor	0.055
3	88949756	89752149	CUB and Sushi multiple domains 1	0.041
3	92589243	92805041	myelin transcription factor 1-like	0.041
3	94137728	94138918	transcription factor SOX-11	0.039
3	101078563	101093823	Matrilin-3	0.054
3	101097118	101110445	lysosomal-associated transmembrane protein 4A	0.054
3	109338625	109369202	regulator of calcineurin 2	0.047
4	1432814	1438671	probable G-protein coupled receptor 174	0.056
4	1447785	1456762	integral membrane protein 2A	0.056
4	3175772	3206798	kelch-like protein 13	0.032
4	5889627	5934997	protein diaphanous homolog 1	0.041
4	15167277	15482153	teneurin-1	0.044
4	15491788	15504671	SH2 domain containing 1A	0.044
4	15612770	15632841	baculoviral IAP repeat-containing protein 4	0.044
4	20652981	20768790	Platelet-derived growth factor C Platelet-derived growth factor C	0.048
4	20823798	20857554	Glycine receptor beta-subunit; Uncharacterized protein	0.048
4	32292871	32664499	LPS-responsive vesicle trafficking, beach and anchor containing	0.046
4	42203702	42635333	UDP-N-acetyl-alpha-D-galactosamine:polypeptide N-acetylgalactosaminyltransferase-like 6	0.054
4	43301385	43363404	glycine receptor, alpha 3	0.054
4	47308221	47790789	latrophilin 3	0.050
4	54233641	54321943	synaptopodin 2	0.055
4	56333065	56644463	Ankyrin 2; Uncharacterized protein	0.048
4	59791983	59835703	endomucin	0.052

4	64565276	64587028	exocyst complex component 1	0.055
4	74015279	74072320	G protein-coupled receptor 125	0.050
4	75401346	75452145	Ligand-dependent nuclear receptor corepressor-like protein	0.031
4	75480300	75503309	non-SMC condensin I complex, subunit G	0.031
4	75524898	75546434	family with sequence similarity 184, member B	0.031
4	75551241	75553275	mediator complex subunit 28	0.031
4	75553556	75565346	cytosol aminopeptidase	0.031
4	76956828	76994096	biorientation of chromosomes in cell division 1-like 1	0.051
4	85000644	85030294	polymerase (RNA) I polypeptide A, 194kDa	0.057
4	86948459	87057215	Catenin alpha-2	0.046
4	87267761	87345268	Catenin alpha-2	0.046
5	543852	563058	deformed epidermal autoregulatory factor 1 homolog	0.050
5	561678	569342	D(4) dopamine receptor	0.050
5	577101	581907	Secretin	0.050
5	582970	596206	cadherin-related family member 5	0.050
5	601359	621958	mitotic checkpoint serine/threonine-protein kinase BUB1 beta	0.050
5	665318	702105	phospholipase C, beta 2	0.050
5	792319	797351	putative TRAF4-associated factor 1	0.050
5	797511	813243	isovaleryl-CoA dehydrogenase, mitochondrial	0.050
5	851698	867344	bromo adjacent homology domain-containing 1 protein	0.050
5	902224	910999	uncharacterized protein C15orf57 homolog	0.050
5	911237	915085	Protein-L-isoaspartate O-methyltransferase	0.050
5	920667	948755	cancer susceptibility candidate 5	0.050
5	950879	957935	DNA repair protein RAD51 homolog 1	0.050
5	969359	999049	regulator of microtubule dynamics 3	0.050
5	1003403	1011083	GTP cyclohydrolase 1 feedback regulatory protein	0.050
5	1011384	1027952	DnaJ (Hsp40) homolog, subfamily C, member 17	0.050
5	1028182	1029979	Olfactory receptor-like protein COR9	0.050
5	1107502	1274845	leucine zipper protein 2	0.050
5	1393483	1395022	olfactory receptor, family 5, subfamily AS, member 1	0.050

5	1434907	1445810	39S ribosomal protein L21, mitochondrial	0.050
5	1452080	1486584	immunoglobulin mu binding protein 2	0.050
5	1461628	1495209	synaptotagmin-12	0.050
5	1496986	1534286	Phosphatidylserine synthase 2	0.050
5	1537037	1551688	anoctamin 9	0.050
5	1555415	1560214	single Ig IL-1-related receptor	0.050
5	1572182	1591429	beta-1,4-N-acetyl-galactosaminyl transferase 4	0.050
5	1600194	1601763	interferon-induced transmembrane protein 5	0.050
5	1616921	1625767	proteasome (prosome, macropain) 26S subunit, non-ATPase, 13	0.050
5	1625769	1629803	sirtuin	0.050
5	1632149	1643201	Synembryn-A	0.050
5	1643314	1647648	blocked early in transport 1 homolog (S. cerevisiae)-like	0.050
5	1649051	1658648	Vacuolar protein sorting-associated protein 51 homolog	0.050
5	1655755	1673396	glutamine-dependent NAD(+) synthetase	0.050
5	1673770	1680338	7-dehydrocholesterol reductase	0.050
5	1699237	1709220	cysteine and glycine-rich protein 3	0.050
5	873373	875881	carbohydrate (N-acetylgalactosamine 4-0) sulfotransferase 14	0.050
5	1041317	1042285	Olfactory receptor-like protein COR9	0.050
5	1046130	1047068	olfactory receptor-like protein COR4	0.050
5	1388197	1389135	olfactory receptor-like protein COR6	0.050
5	5014653	5046383	dnaJ homolog subfamily C member 24	0.053
5	14243984	14284324	Mucin-5B	0.042
5	14423044	14459159	Mucin-6	0.042
5	14460720	14503618	adaptor-related protein complex 2, alpha 2 subunit	0.042
5	14508350	14612966	chitinase domain-containing protein 1 precursor	0.042
5	14631769	14696703	tetraspanin 4	0.042
5	15481413	15488239	ribonuclease inhibitor	0.057
5	16622191	16622361	TUC338	0.044
5	18137578	18152041	probable methylthioribulose-1-phosphate dehydratase	0.055
5	18152188	18191503	pyruvate dehydrogenase complex, component X	0.055

5	18248884	18303314	CD44 antigen precursor	0.055
5	18316563	18391081	excitatory amino acid transporter 2	0.055
5	18394011	18447963	peptidase domain containing associated with muscle regeneration 1	0.055
5	20702880	20716727	Apoptosis inhibitor 5	0.053
5	20718591	20772087	tetratricopeptide repeat domain 17	0.053
5	20863830	20943207	hydroxysteroid (17-beta) dehydrogenase 12	0.053
5	21901389	21963072	alpha-(1,6)-fucosyltransferase	0.025
5	22080490	22083151	family with sequence similarity 180, member B	0.025
5	22088781	22093418	NADH dehydrogenase	0.025
5	22095231	22100669	kelch repeat and BTB domain-containing protein 4	0.025
5	22100815	22102871	protein tyrosine phosphatase, mitochondrial 1	0.025
5	22103005	22150651	CUGBP Elav-like family member 1	0.025
5	22161202	22170398	43 kDa receptor-associated protein of the synapse	0.025
5	22170650	22181783	26S protease regulatory subunit 6A	0.025
5	22196883	22215277	Zinc transporter ZIP13	0.025
5	22247550	22268506	transcription factor PU.1	0.025
5	22076422	22077558	C1q and tumor necrosis factor related protein 4	0.025
5	25492780	25585952	protein numb homolog	0.055
5	30506655	30675196	homeobox protein Meis2	0.038
5	30717442	30832671	uncharacterized protein C15orf41 homolog	0.038
5	31253451	31438936	ATP-binding domain-containing protein 4	0.038
5	31481161	31528089	aquarius homolog (mouse)	0.038
5	31546121	31552902	Actin, alpha cardiac muscle 1	0.038
5	31568439	31572498	gap junction delta-2 protein	0.038
5	31693846	31748417	syntaxin-binding protein 6	0.038
5	30662303	30662490	TUC338	0.038
5	30589905	30590067	TUC338	0.038
5	40042540	40090213	thyrotropin receptor isoform 1 precursor	0.041
5	40099534	40119234	transcription initiation factor IIA subunit 1	0.041
5	40144828	40210981	stonin 2	0.041

5	50044677	50054344	tumor necrosis factor, alpha-induced protein 2	0.048
6	452392	518973	granule cell antiserum positive 14	0.056
6	6129271	6562977	catenin alpha-3	0.040
6	11114347	11184726	sphingosine-1-phosphate lyase 1	0.043
6	11185502	11189454	Pterin-4-alpha-carbinolamine dehydratase	0.043
6	12928166	13365708	calcium-activated potassium channel subunit alpha-1	0.042
6	15388339	15449462	uncharacterized protein LOC423739	0.042
6	15493933	15500233	alpha-(1,3)-fucosyltransferase 11 precursor	0.042
6	15645287	15696643	ubiquitin specific peptidase 54	0.042
6	15705273	15750035	serine/threonine-protein phosphatase 2B catalytic subunit beta isoform	0.042
6	15752205	15762949	Annexin	0.042
6	15922043	15977706	SH3 domain containing ring finger 1	0.042
6	16111040	16115551	zona pellucida sperm-binding protein 4	0.042
6	17984757	18098872	WDFY family member 4	0.033
7	295799	302486	asparagine synthetase domain-containing protein 1	0.035
7	331708	343582	solute carrier family 40 member 1	0.035
7	571200	611944	collagen alpha-1(III) chain precursor	0.035
7	2220524	2272149	NCK-associated protein 1	0.041
7	2280810	2296210	secreted frizzled-related protein 3 precursor	0.041
7	2460244	2525374	Microtubule-associated protein	0.041
7	2653627	2655073	ribulose-5-phosphate-3-epimerase	0.041
7	2659245	2710824	KAT8 regulatory NSL complex subunit 1-like	0.041
7	2715471	2729321	long-chain specific acyl-CoA dehydrogenase, mitochondrial	0.041
7	2739204	2756780	Myosin light chain 1, skeletal muscle isoform	0.041
7	2772090	2785749	lanC-like protein 1	0.041
7	2785930	2886932	carbamoyl-phosphate synthase	0.041
7	3031382	3433912	receptor tyrosine-protein kinase erbB-4	0.041
7	4154100	4197932	BRCA1-associated RING domain protein 1	0.046
7	4209985	4288793	ATP-binding cassette, sub-family A (ABC1), member 12	0.046
7	4312646	4330369	bifunctional purine biosynthesis protein PURH	0.046

7	4333468	4382817	fibronectin precursor	0.046
7	4527554	4590262	NEDD8-conjugating enzyme UBE2F	0.046
7	4591385	4595864	receptor (G protein-coupled) activity modifying protein 1	0.046
7	6552331	6592902	NADH dehydrogenase	0.047
7	12072509	12076665	elongation factor 1-beta	0.053
7	12077169	12090936	NADH-ubiquinone oxidoreductase 75 kDa subunit, mitochondrial	0.053
7	12074025	12074158	Small nucleolar RNA SNORA41	0.053
7	12074705	12074740	Small nucleolar RNA Z196/R39/R59 family	0.053
7	13103070	13154191	abl-interactor 2	0.047
7	13299094	13309416	Isocitrate dehydrogenase	0.047
7	13452385	13595936	phosphodiesterase 1A, calmodulin-dependent	0.047
7	13656874	13697980	sperm specific antigen 2	0.047
7	13767734	13821804	ceramide kinase-like	0.047
7	14019120	14074118	ubiquitin-conjugating enzyme E2 E3	0.047
7	14281313	14312291	pre-mRNA-splicing factor CWC22 homolog	0.047
7	13758808	13759956	neurogenic differentiation factor 1	0.047
7	13572929	13573037	U4 spliceosomal RNA	0.047
7	19657871	19705480	growth factor receptor-bound protein 14	0.050
7	26051937	26233120	sema domain, seven thrombospondin repeats (type 1 and type 1-like)	0.049
7	28420755	28433434	DEAD (Asp-Glu-Ala-Asp) box polypeptide 18	0.053
7	28692505	28874290	dipeptidyl-peptidase 10 (non-functional)	0.053
7	31350097	31932431	low density lipoprotein receptor-related protein 1	0.053
7	32211979	32530918	Rho GTPase-activating protein 15	0.051
8	7093244	7291205	rab GTPase-activating protein 1-like	0.046
8	7324976	7355098	ring finger and CCCH-type domains 1	0.046
8	7367591	7372515	serpin peptidase inhibitor, clade C (antithrombin), member 1	0.046
8	7398818	7402043	heme binding protein 2	0.046
8	7411851	7421087	N-acetylneuraminate lyase	0.046
8	7449296	7493463	laminin, gamma 1 (formerly LAMB2)	0.046
8	7392831	7392897	Small nucleolar RNA SNORD75	0.046

8	7397456	7397530	Small nucleolar RNA SNORD47	0.046
8	7393134	7393217	Small nucleolar RNA SNORD24	0.046
8	7396328	7396406	Small nucleolar RNA SNORD79	0.046
8	7392012	7392089	Small nucleolar RNA SNORD74	0.046
8	7397669	7397747	Small nucleolar RNA SNORD81	0.046
8	7393569	7393651	Small nucleolar RNA snR60/Z15/Z230/Z193/J17	0.046
8	7393820	7393903	Small nucleolar RNA snR60/Z15/Z230/Z193/J17	0.046
8	11522078	11839939	dihydropyrimidine dehydrogenase	0.042
8	11896946	11945494	polypyrimidine tract binding protein 2	0.042
8	18082728	18146552	GPI-anchor transamidase precursor	0.055
8	18155675	18221761	Alpha-2,6-sialyltransferase ST6GalNAc V; Uncharacterized protein	0.055
8	18504655	18513032	PIF1 5'-to-3' DNA helicase	0.055
8	18560220	18571250	tyrosine kinase with immunoglobulin-like and EGF-like domains 1	0.055
8	18572741	18576864	thrombopoietin receptor precursor	0.055
8	18577132	18582553	cell division cycle protein 20 homolog	0.055
8	18585120	18597571	elongation of very long chain fatty acids protein 1	0.055
8	18671922	18675086	hydroxypyruvate isomerase (putative)	0.055
8	20565276	20583037	diencephalon/mesencephalon homeobox 1	0.057
8	23276661	23291886	origin recognition complex subunit 1	0.033
8	23344643	23353210	glutathione peroxidase 7	0.033
8	23364732	23378739	family with sequence similarity 159, member A	0.033
8	23434614	23446751	Non-specific lipid-transfer protein	0.033
8	23550024	23551699	Protein mago nashi homolog	0.033
8	23561280	23710577	low-density lipoprotein receptor-related protein 8 precursor	0.033
8	23769011	23778972	doublesex- and mab-3-related transcription factor B1	0.033
8	23804849	23900621	GLIS family zinc finger 1	0.033
8	27623284	27647208	protein wntless homolog precursor	0.056
9	3921563	3937753	insulin-like growth factor 2 mRNA binding protein 2	0.051
9	9694581	9736770	ras GTPase-activating protein 2	0.039
9	9743479	9749897	RING-box protein 2	0.039

9	9788144	9809727	Sodium/potassium-transporting ATPase subunit beta-3	0.039
9	9818914	9863003	transcription factor Dp-2	0.039
9	9877217	9898347	Putative glycerol kinase 5	0.039
9	11379753	11384969	Zic family member 4	0.049
9	11938121	11940148	phosphatidylinositol glycan anchor biosynthesis, class Z	0.049
9	11940276	11956868	melanotransferrin precursor	0.049
9	11959073	12089444	discs, large homolog 1 (Drosophila)	0.049
9	12115163	12127577	D-beta-hydroxybutyrate dehydrogenase, mitochondrial precursor	0.049
9	11878868	11880012	Type-1 angiotensin II receptor	0.049
9	21936183	21948704	myeloid leukemia factor 1	0.053
9	21958768	22066377	arginine/serine-rich coiled-coil 1	0.053
10	12540336	12583712	Aggrecan core protein	0.052
10	13154344	13445021	ATP/GTP binding protein-like 1	0.052
11	2154521	2175204	autocrine motility factor receptor, E3 ubiquitin protein ligase	0.046
11	2195648	2306313	guanine nucleotide binding protein (G protein), alpha activating activity polypeptide O	0.046
11	5418071	5433815	sal-like protein 1	0.041
11	6524058	6525793	cerebellin 1 precursor	0.047
11	6773329	6790051	NEDD4-binding protein 1	0.047
11	6864118	6903373	peroxisomal Lon protease homolog 2	0.047
11	6992610	7061529	phosphorylase b kinase regulatory subunit beta	0.047
11	7202523	7210231	dnaJ homolog subfamily A member 2	0.047
11	7284405	7304607	myosin light chain kinase 3	0.047
11	7307255	7312433	origin recognition complex, subunit 6	0.047
11	ĂĂĂĂ585	7334449	vacuolar protein sorting-associated protein 35	0.047
11	7351098	7365112	SHC SH2-domain binding protein 1	0.047
11	7596758	7603866	Cytochrome b-c1 complex subunit Rieske, mitochondrial	0.047
11	12288497	12432361	cadherin-8	0.039
11	13255162	13323250	ADAM metallopeptidase with thrombospondin type 1 motif, 18	0.039
11	13424046	13427036	nudix (nucleoside diphosphate linked moiety X)-type motif 7	0.039
11	12276171	12276338	TUC338	0.039

11	12638991	12639143	TUC338	0.039
11	14761130	14784372	chromodomain protein, Y-like 2	0.053
11	17486311	17616586	protein BANP isoform 2	0.053
12	3504495	3594440	isoleucyl-tRNA synthetase	0.049
13	185486	285392	protocadherin alpha 11 precursor	0.045
13	4187413	4738485	teneurin-2 isoform 1	0.048
13	13578334	13606087	collagen, type XXIII, alpha 1	0.054
13	13614747	13621757	5-phosphohydroxy-L-lysine phospho-lyase	0.054
13	13624726	13631823	heterogeneous nuclear ribonucleoprotein A/B	0.054
13	13640151	13649060	NME/NM23 family member 5	0.054
13	13661789	13665719	protein Wnt-8c precursor	0.054
13	13695529	13725044	family with sequence similarity 13, member B	0.054
13	13728686	13739566	polycystic kidney disease 2-like 2	0.054
13	13818620	13851076	kelch-like family member 3	0.054
13	13906202	14163956	sparc/osteonectin, cwcv and kazal-like domains proteoglycan (testican) 1	0.054
13	13786987	13788111	neuropeptide Y receptor type 6	0.054
14	5230050	5338554	ent T-type calcium channel subunit alpha-1H	0.053
14	9934515	9974931	ubiquitin carboxyl-terminal hydrolase 7	0.050
14	10846837	11212402	RNA binding protein, fox-1 homolog (C. elegans) 1	0.046
16	225233	229543	kinesin family member C1	0.045
16	291965	332200	intestinal zipper protein	0.045
16	335307	346680	MHC B-G antigen isoform 2 precursor	0.045
17	2100704	2358309	Voltage-dependent N-type calcium channel subunit alpha-1B	0.043
17	5207121	5216583	protein SET	0.041
17	5218454	5236535	protein kinase N3	0.041
17	5284710	5289952	cysteine conjugate-beta lyase, cytoplasmic	0.041
17	5404390	5430983	nucleoporin NUP188 homolog	0.041
17	5432744	5457800	Endophilin-B2	0.041
17	5477916	5490687	dolichyl pyrophosphate phosphatase 1	0.041
17	5509515	5533835	serine/threonine-protein phosphatase 2A activator	0.041

17	5646584	5651589	ankyrin repeat and SOCS box protein 6	0.041
17	5655699	5671868	Paired mesoderm homeobox protein 2	0.041
17	5676177	5679429	prostaglandin E synthase	0.041
17	5700662	5704295	torsin-1A precursor	0.041
17	5709761	5728577	ubiquitin specific peptidase 20	0.041
17	5732998	5792450	formin binding protein 1	0.041
17	5824408	5854549	protein GPR107 precursor	0.041
17	5864357	5873610	Neuronal calcium sensor 1	0.041
17	5881860	5902534	argininosuccinate synthase	0.041
17	5911623	5947740	far upstream element-binding protein 3	0.041
17	5964138	5968638	exosome component 2	0.041
17	6773214	6778591	carboxyl ester lipase precursor	0.038
17	6780509	6787429	general transcription factor 3C polypeptide 5	0.038
17	6830389	6837593	globoside alpha-1,3-N-acetylgalactosaminyltransferase 1	0.038
17	6856076	6858808	surfeit locus protein 6	0.038
17	6864523	6865513	Mediator of RNA polymerase II transcription subunit 22	0.038
17	6866152	6869274	60S ribosomal protein L7a	0.038
17	6869498	6873120	Surfeit locus protein 1	0.038
17	6873177	6876256	surfeit locus protein 2	0.038
17	6878957	6889748	Surfeit locus protein 4	0.038
17	6900726	6906262	REX4, RNA exonuclease 4 homolog (S. cerevisiae)	0.038
17	6906389	6925818	ADAM metallopeptidase with thrombospondin type 1 motif, 13	0.038
17	6936944	6940561	solute carrier family 2 (facilitated glucose transporter), member 6	0.038
17	7013210	7024311	Dopamine beta-hydroxylase	0.038
17	7026160	7045384	sarcosine dehydrogenase	0.038
17	7049744	7148021	guanine nucleotide exchange factor VAV2	0.038
17	7198594	7208667	WD repeat-containing protein 5	0.038
17	7329258	7353026	retinoid X receptor, alpha	0.038
17	7462510	7561096	collagen alpha-1(V) chain precursor	0.038
17	7609613	7627804	Noelin	0.038

17	7710495	7713122	protein phosphatase 1, regulatory subunit 26	0.038
17	7737254	7744146	1-acylglycerol-3-phosphate O-acyltransferase 2	0.038
17	7809850	7842342	NOTCH-1; Uncharacterized protein	0.038
17	7723324	7723456	Small nucleolar RNA SNORA17	0.038
17	6867450	6867526	Small nucleolar RNA SNORD36	0.038
17	6866555	6866629	Small nucleolar RNA SNORD24	0.038
17	7209751	7209878	U6atac minor spliceosomal RNA	0.038
17	7722798	7722930	Small nucleolar RNA SNORA17	0.038
17	6867896	6867968	Small nucleolar RNA SNORD36	0.038
18	4283804	4289327	alpha-N-acetylgalactosaminide alpha-2,6-sialyltransferase 2	0.057
18	6254682	6261401	tripartite motif containing 25	0.050
18	6262355	6267812	coilin	0.050
18	6267875	6278615	serine carboxypeptidase 1 precursor	0.050
18	6282874	6369966	RAB11 family interacting protein 4 (class II)	0.050
18	10393676	10406868	luc7-like protein 3	0.035
18	10407626	10414276	ankyrin repeat domain-containing protein 40	0.035
18	10415256	10447157	ATP-binding cassette, sub-family C (CFTR/MRP), member 3	0.035
18	10578502	10590280	spermatogenesis associated 20	0.035
18	10591546	10596249	epsin 3	0.035
18	10670853	10683456	xylosyltransferase 2	0.035
18	10683850	10688333	CD300a molecule precursor	0.035
18	10690316	10691046	uncharacterized protein LOC769812 precursor	0.035
18	10703909	10709349	RAB37, member RAS oncogene family	0.035
18	10711117	10718410	Na(+)/H(+) exchange regulatory cofactor NHE-RF1	0.035
18	10769812	10777868	glutamate receptor, ionotropic, N-methyl D-aspartate 2C	0.035
18	10812362	10827732	Usher syndrome 1G (autosomal recessive)	0.035
18	10823003	10827945	otopetrin 2	0.035
18	10832874	10838829	otopetrin 3	0.035
18	10867250	10883572	cerebellar degeneration-related protein 2-like	0.035
18	10900894	10907983	BTB/POZ domain-containing protein KCTD2	0.035

18	10354079	10355879	protein Tob1	0.035
19	4473399	4477417	DNA repair protein RAD51 homolog 4	0.046
19	4478902	4502458	E3 ubiquitin-protein ligase rififylin	0.046
19	4503027	4518242	DNA ligase 3	0.046
19	4788614	4789702	Chemokine	0.046
19	4790687	4791888	chemokine (C-C motif) ligand 1 precursor	0.046
19	4792410	4793550	Chemokine; Uncharacterized protein	0.046
19	4812218	4813843	uncharacterized protein LOC417536 precursor	0.046
19	4852010	4866040	protein NipSnap homolog 2	0.046
19	4870122	4880847	phosphoserine phosphatase	0.046
19	4884981	4891209	T-complex protein 1 subunit zeta	0.046
19	4896318	4901236	phosphorylase b kinase gamma catalytic chain, skeletal muscle isoform	0.046
19	4905637	4915760	vitamin K epoxide reductase complex subunit 1-like protein 1	0.046
19	4921170	4931757	beta-glucuronidase precursor	0.046
19	4932422	4939398	argininosuccinate lyase	0.046
19	4945236	4952988	Delta-1 crystallin	0.046
19	4886414	4886545	Small nucleolar RNA SNORA22	0.046
19	4887558	4887689	Small nucleolar RNA SNORA15	0.046
19	9192393	9211569	nitric oxide synthase, inducible	0.048
20	9837210	9851741	Uridine kinase	0.047
20	9883864	9899970	tumor protein D54	0.047
20	10917368	10921453	matrix metalloproteinase-9 precursor	0.048
20	13433463	13447739	sal-like protein 4	0.031
20	13454134	13507093	ATPase, class II, type 9A	0.031
20	13731017	13739373	dolichyl-phosphate mannosyltransferase polypeptide 1, catalytic subunit	0.031
20	13811824	13824523	partitioning defective 6 homolog beta	0.031
20	13824100	13879197	family with sequence similarity 65, member C	0.031
20	13883933	13922802	Tyrosine-protein phosphatase non-receptor type 1	0.031
20	14084227	14098181	Ubiquitin-conjugating enzyme E2 variant 1	0.031
20	14054696	14055682	CCAAT/enhancer-binding protein beta	0.031

20	14046407	14046516	TUC338	0.031
21	1432928	1434563	transcription factor HES-5	0.045
21	1435527	1459981	pantothenate kinase 4	0.045
21	1456791	1466358	Probable glutamate receptor	0.045
21	1552285	1555875	peroxisome biogenesis factor 10	0.045
21	1558528	1565754	Protein RER1	0.045
21	1679120	1769271	ski oncogene	0.045
22	243826	248360	gastrokine 1	0.027
22	276543	282395	Bone morphogenetic protein-10; Uncharacterized protein	0.027
22	291968	308523	rho GTPase-activating protein 25	0.027
22	314518	323055	Phosphatidate cytidylyltransferase	0.027
22	1989333	2026713	potassium channel, subfamily U, member 1	0.046
22	2203023	2212432	G protein-coupled receptor 124	0.046
22	2219556	2228649	RAB11 family interacting protein 1 (class I)	0.046
22	2230210	2232651	prolactin-releasing peptide receptor-like protein	0.046
22	2256515	2263926	set1/Ash2 histone methyltransferase complex subunit ASH2	0.046
22	2263879	2266815	Steroidogenic acute regulatory protein, mitochondrial	0.046
22	2268642	2270014	LSM1 homolog, U6 small nuclear RNA associated (S. cerevisiae)	0.046
22	2269948	2273194	BCL2-associated athanogene 4	0.046
22	2274522	2282463	DDHD domain containing 2	0.046
22	2282970	2285701	phosphatidic acid phosphatase type 2 domain containing 1B	0.046
22	2321653	2327164	leucine zipper-EF-hand containing transmembrane protein 2	0.046
22	2331115	2345392	fibroblast growth factor receptor 1 precursor	0.046
23	114057	143603	Ribosomal protein S6 kinase 2 alpha	0.053
23	155931	159391	Non-histone chromosomal protein HMG-17	0.053
23	3153762	3156554	family with sequence similarity 110, member D	0.042
23	3186847	3190095	platelet-activating factor acetylhydrolase 2, 40kDa	0.042
23	3198617	3201919	Stathmin	0.042
23	3204152	3207296	progestin and adipoQ receptor family member VII	0.042
23	3217150	3225967	ras-related GTP-binding protein C	0.042

23	3502047	3514489	metal regulatory transcription factor 1	0.042
23	3551602	3581930	EPH receptor A10	0.042
23	3708593	3713721	MYST/Esa1-associated factor 6	0.042
23	3805041	3874419	glutamate receptor, ionotropic, kainate 3	0.042
23	3965824	3970617	granulocyte colony-stimulating factor receptor	0.042
23	3977706	3987124	organic solute carrier partner 1	0.042
23	3988933	4004007	serine/threonine-protein kinase 40	0.042
23	4007214	4007707	eva-1 homolog B (C. elegans)	0.042
23	4008688	4024899	thyroid hormone receptor-associated protein 3	0.042
23	4031627	4038165	MAP7 domain containing 1	0.042
23	4050995	4056202	Trafficking protein particle complex subunit 3	0.042
23	4092973	4097739	poly(ADP-ribose) glycohydrolase ARH3	0.042
23	4098117	4102886	tektin 2 (testicular)	0.042
23	4111971	4133770	protein argonaute-3	0.042
23	4176483	4190009	claspin	0.042
23	4222202	4234872	Proteasome subunit beta type	0.042
23	4256435	4263091	neurochondrin	0.042
23	4264511	4288627	KIAA0319-like	0.042
23	4295102	4296014	interferon alpha-inducible protein 27-like protein 2	0.042
23	4343978	4352202	Gizzard PTB-associated splicing factor; Uncharacterized protein	0.042
23	4361390	4361969	ZMYM6 neighbor	0.042
23	4379948	4393033	discs, large (Drosophila) homolog-associated protein 3	0.042
23	4398099	4400981	connexin 37	0.042
23	4570631	4804042	CUB and Sushi multiple domains 2	0.042
23	4824370	4834663	collagen, type IX, alpha 2	0.042
23	4835192	4847417	small ArfGAP2	0.042
23	4901524	4907272	potassium voltage-gated channel, KQT-like subfamily, member 4	0.042
23	4910836	4916448	tubulointerstitial nephritis antigen-like 1	0.042
23	4918792	4920966	penta-EF-hand domain containing 1	0.042
23	4921631	4939423	collagen, type XVI, alpha 1	0.042

23	4943828	4960852	brain-specific angiogenesis inhibitor 2	0.042
23	5033894	5048705	KH domain-containing, RNA-binding, signal transduction-associated protein 1	0.042
23	5072000	5074434	MARCKS-related protein	0.042
23	5075963	5089294	Histone deacetylase 1	0.042
23	5103396	5107519	eukaryotic translation initiation factor 3 subunit I	0.042
23	5108365	5113741	doublecortin domain containing 2B	0.042
23	5118998	5126267	taxilin alpha	0.042
23	5126931	5142990	importin subunit alpha-7	0.042
23	5145916	5152332	BSD domain-containing protein 1	0.042
23	5179676	5186712	histone-binding protein RBBP4	0.042
23	5208687	5215113	KIAA1522	0.042
23	5216609	5221415	tyrosyl-tRNA synthetase, cytoplasmic	0.042
23	5229742	5235821	fibronectin type III domain containing 5	0.042
23	3211327	3212868	gap junction protein, alpha 9, 59kDa	0.042
23	4404012	4404779	gap junction protein, beta 3, 31kDa	0.042
24	27288	31762	dolichyl-phosphate (UDP-N-acetylglucosamine) N-acetylglucosaminephosphotransferase 1	0.037
24	62299	94625	salt-inducible kinase 2	0.037
24	121487	131479	fasciculation and elongation protein zeta 1 (zygin I)	0.037
24	134977	155779	homeobox protein PKNOX2	0.037
24	209825	217136	solute carrier family 37 (glucose-6-phosphate transporter), member 2	0.037
24	235948	239677	hepatic and glial cell adhesion molecule	0.037
24	241553	246273	roundabout, axon guidance receptor, homolog 4 (Drosophila)	0.037
24	247239	255967	roundabout, axon guidance receptor, homolog 3 (Drosophila)	0.037
24	264021	283376	uncharacterized protein C11orf61 homolog	0.037
24	289700	292570	endothelial cell adhesion molecule	0.037
24	294677	296798	neurogranin (protein kinase C substrate, RC3)	0.037
24	309430	315623	pseudouridylate synthase 3	0.037
24	322587	362342	cell adhesion associated, oncogene regulated	0.037
24	373687	377118	serine/threonine-protein kinase Chk1	0.037
24	384626	392693	etoposide induced 2.4 mRNA	0.037

24	395902	404137	signal recognition particle receptor subunit alpha	0.037
24	404255	412337	uncharacterized protein LOC419714	0.037
24	412584	417125	toll/interleukin-1 receptor domain-containing adapter protein	0.037
24	442597	449861	ST3 beta-galactoside alpha-2,3-sialyltransferase 4	0.037
24	32960	33547	H2A histone family, member X	0.037
24	4392700	4566947	ubiquitin conjugation factor E4 A	0.051
24	5803421	5884198	neural cell adhesion molecule 1 precursor	0.048
25	1522828	1528457	threonyl-tRNA synthetase 2, mitochondrial (putative)	0.041
25	1531522	1538194	regulation of nuclear pre-mRNA domain containing 2	0.041
25	1548432	1559282	U4/U6 small nuclear ribonucleoprotein Prp3	0.041
25	1566333	1569413	carbonic anhydrase XIV	0.041
25	1569584	1573307	APH1A gamma secretase subunit	0.041
25	1587904	1591630	Fc receptor family member	0.041
25	1655296	1671257	cell adhesion molecule 3	0.041
25	1674627	1675765	C-reactive protein precursor	0.041
25	1678200	1680026	dual specificity phosphatase 23	0.041
25	1696346	1699747	translocon-associated protein subunit beta precursor	0.041
25	1700503	1705069	cathepsin S precursor	0.041
25	1705617	1707797	cathepsin K precursor	0.041
25	1709351	1730021	aryl hydrocarbon receptor nuclear translocator	0.041
25	1751126	1755032	family with sequence similarity 63, member A	0.041
25	1761749	1763263	CDC42 small effector protein 1	0.041
25	1777988	1783588	sema domain, transmembrane domain (TM), and cytoplasmic domain, (semaphorin) 6C	0.041
25	1788992	1961542	tropomodulin 4 (muscle)	0.041
25	1792982	1795236	vacuolar protein sorting 72 homolog (S. cerevisiae)	0.041
25	1813687	1817715	proteasome (prosome, macropain) 26S subunit, non-ATPase, 4	0.041
25	1930800	1933117	CDC42 small effector protein 1	0.041
25	1934174	1936824	myeloid/lymphoid or mixed-lineage leukemia (trithorax homolog, Drosophila)	0.041
25	1954448	1956826	sodium channel modifier 1	0.041
25	1963704	1979923	phosphatidylinositol-4-phosphate 5-kinase, type I, alpha	0.041

25	2068269	2086129	pogo transposable element with ZNF domain	0.041
25	2086672	2089191	Proteasome subunit beta type	0.041
25	2093995	2102545	cingulin	0.041
25	1672509	1673429	Duffy blood group, atypical chemokine receptor	0.041
26	16131	27205	NEDD9-interacting protein with calponin homology and LIM domains	0.020
26	33325	43701	TEA domain family member 3	0.020
26	55541	59279	tubby-related protein 1	0.020
26	61868	80894	peptidyl-prolyl cis-trans isomerase FKBP5	0.020
26	87655	88370	colipase precursor	0.020
26	89352	93029	Tetraspan membrane protein of hair cell stereocilia homolog	0.020
26	95396	113328	SRSF protein kinase 1	0.020
26	184444	202634	bromodomain and PHD finger containing, 3	0.020
26	224386	227839	pim-1 oncogene	0.020
26	246613	275469	Voltage-dependent L-type calcium channel subunit alpha-1S	0.020
26	276159	308776	kinesin family member 21B	0.020
26	1342854	1345933	RNA binding motif protein 15	0.057
26	1356714	1365537	solute carrier family 16, member 4 (monocarboxylic acid transporter 5)	0.057
26	1367168	1369391	hepatitis B virus x interacting protein	0.057
26	1371526	1374791	prokineticin 1	0.057
26	1376304	1379433	embryonic pepsinogen precursor	0.057
26	1468607	1473077	PNPLA1	0.057
26	1482961	1487317	ets variant 7	0.057
26	1521378	1522415	Parathyroid hormone-like peptide	0.057
26	1527549	1533439	splicing factor, arginine/serine-rich 3	0.057
26	1543127	1545030	cdk inhibitor CIP1 (p21)	0.057
26	1545390	1549195	EF-hand calcium binding domain 4B	0.057
26	1553858	1557199	RAB44, member RAS oncogene family	0.057
26	1557671	1579275	uncharacterized protein C6orf89 homolog	0.057
26	1620031	1622178	Small nuclear ribonucleoprotein E	0.057
26	1629296	1655910	SRY (sex determining region Y)-box 13	0.057

26	1658325	1665181	ethanolamine kinase 2	0.057
26	1667696	1671368	renin	0.057
26	1676675	1717188	pleckstrin homology domain containing, family A member 6	0.057
26	1732339	1747306	phosphatidylinositol-4-phosphate 3-kinase, catalytic subunit type 2 beta	0.057
26	1381507	1383057	Potassium voltage-gated channel subfamily A member 10	0.057
26	1413003	1414502	potassium voltage-gated channel subfamily A member 2	0.057
26	1435026	1436588	potassium voltage-gated channel subfamily A member 3	0.057
26	1783648	1785738	leucine rich repeat neuronal 2	0.057
26	1592118	1592200	gga-let-7k	0.057
26	1591918	1592000	gga-let-7j	0.057
26	2417505	2427240	inhibitor of kappa light polypeptide gene enhancer in B-cells, kinase epsilon	0.053
26	2438015	2447837	Ras association (RalGDS/AF-6) domain family member 5	0.053
26	2446906	2458197	eukaryotic translation initiation factor 2D	0.053
26	2504099	2506388	Interleukin-10	0.053
26	2517673	2519712	interleukin 19	0.053
26	2520032	2531914	polymeric immunoglobulin receptor precursor	0.053
26	2556703	2560172	ubiquitin thioesterase OTU1	0.053
26	2576519	2583374	C4b-binding protein alpha chain precursor	0.053
26	2599568	2605277	complement component (3b/4b) receptor 1-like precursor	0.053
26	2627129	2639856	complement component 4 binding protein, alpha chain precursor	0.053
26	4960037	4974337	transcription factor EB	0.048
26	4976148	4979037	progastricsin (pepsinogen C)	0.048
26	4980962	4984806	gastricsin precursor	0.048
26	4989811	4997320	fibroblast growth factor receptor substrate 3	0.048
26	5001119	5006673	prickle homolog 4 (Drosophila)	0.048
26	5028663	5038433	Ubiquitin carboxyl-terminal hydrolase	0.048
26	5056220	5060765	Mediator of RNA polymerase II transcription subunit 20	0.048
26	5060817	5063516	bystin-like	0.048
26	5065132	5077551	G1/S-specific cyclin-D3	0.048
27	116781	119543	Dolichyl-diphosphooligosaccharide--protein glycosyltransferase subunit DAD1	0.022

27	1056370	1079949	gametocyte specific factor 1	0.022
27	1081554	1087770	Golgi SNAP receptor complex member 2	0.022
27	1111810	1122644	Protein Wnt	0.022
27	18285	19232	olfactory receptor, family 6, subfamily A, member 2	0.022
27	2226991	2253136	corticotropin-releasing factor receptor 1 precursor	0.049
27	2302640	2319626	integrin beta-3 precursor	0.049
27	2332100	2344617	Methyltransferase-like protein 2	0.049
27	2357455	2399073	tousled-like kinase 2	0.049
27	2412671	2426010	mannose receptor, C type 2	0.049
27	2524612	2629807	tetratricopeptide repeat, ankyrin repeat and coiled-coil containing 2	0.049
27	2634670	2639226	cytochrome b561	0.049
27	2646484	2662837	angiotensin-converting enzyme precursor	0.049
27	2671507	2696162	potassium voltage-gated channel, subfamily H (eag-related), member 6	0.049
27	2698550	2714282	DDB1- and CUL4-associated factor 7	0.049
27	2764310	2769010	LIM domain-containing protein 2	0.049
27	2775142	2781204	ring finger protein 113A	0.049
27	2782104	2793046	STE20-related kinase adapter protein alpha	0.049
27	2803637	2820051	ATP-dependent RNA helicase DDX42	0.049
27	2822027	2826383	Parathyroid hormone receptor 3	0.049
27	2837938	2864740	cell division cycle protein 27 homolog	0.049
27	2884724	2934787	KAT8 regulatory NSL complex subunit 1	0.049
27	4505836	4644100	Eukaryotic translation initiation factor 1	0.030
27	4618778	4622608	keratin, type I cytoskeletal 14	0.030
27	4645878	4646526	gastrin/cholecystokinin-like peptide precursor	0.030
27	4647054	4655682	huntingtin-associated protein 1	0.030
27	4658140	4665314	Plakoglobin	0.030
27	4674822	4678761	leprecan-like 4	0.030
27	4679153	4685639	FK506 binding protein 10, 65 kDa	0.030
27	4685681	4690379	Cytosolic 5'-nucleotidase III-like protein	0.030
27	4698639	4700783	kelch-like family member 11	0.030

27	4701712	4720355	ATP-citrate synthase	0.030
27	4733938	4738865	2',3'-cyclic nucleotide 3' phosphodiesterase	0.030
27	4739049	4758336	dnaJ homolog subfamily C member 7	0.030
27	4758782	4759794	NF-kappa-B inhibitor-interacting Ras-like protein 2	0.030
27	4822592	4826644	DEXH (Asp-Glu-X-His) box polypeptide 58	0.030
27	4827918	4832197	histone acetyltransferase KAT2A	0.030
27	4837933	4841685	Ras-related protein Rab-5C	0.030
27	4847654	4856867	potassium voltage-gated channel, subfamily H (eag-related), member 4	0.030
27	4857256	4858313	hypocretin (orexin) neuropeptide precursor	0.030
27	4871019	4873562	GH3 domain containing	0.030
27	4875164	4886606	signal transducer and activator of transcription 5B	0.030
27	4896267	4907552	Signal transducer and activator of transcription 3	0.030
27	4913996	4926852	polymerase I and transcript release factor	0.030
27	4929945	4958104	V-type proton ATPase 116 kDa subunit a isoform 1	0.030
27	4962219	4963268	17-beta-hydroxysteroid dehydrogenase	0.030
27	4963812	4965610	CoA synthase	0.030
27	4967021	4970406	max-like protein X	0.030
27	4970565	4973603	PSMC3 interacting protein	0.030
27	4982347	4989442	tubulin, gamma 1	0.030
27	4990263	4995457	pleckstrin homology domain containing, family H (with MyTH4 domain) member 3	0.030
27	5003802	5012710	contactin associated protein 1	0.030
27	5030186	5032034	receptor activity-modifying protein 2 precursor	0.030
27	5039150	5051893	WNK lysine deficient protein kinase 4	0.030
27	5055739	5060519	beclin-1	0.030
27	5060694	5067398	Proteasome activator complex subunit 3	0.030
27	5095037	5098091	RUN domain containing 1	0.030
27	5099248	5101501	60S ribosomal protein L27	0.030
27	5102844	5105307	interferon-induced protein 35	0.030
27	5126612	5140086	rho-related GTP-binding protein RhoN	0.030
27	5150701	5171059	breast cancer 1, early onset	0.030

27	5171677	5189435	neighbor of BRCA1 gene 1	0.030
27	5000132	5001181	chemokine (C-C motif) receptor 10	0.030
27	5173809	5173871	Neighbour of BRCA1 gene 2 converved region	0.030
28	509129	534490	heterogeneous nuclear ribonucleoprotein M	0.057
28	1630487	1705053	long-chain-fatty-acid--CoA ligase ACSBG2	0.045
28	1650455	1678571	myeloid/lymphoid or mixed-lineage leukemia (trithorax homolog, Drosophila)	0.045
28	1712018	1720854	Acidic leucine-rich nuclear phosphoprotein 32 family member B	0.045
28	1729378	1742502	myosin-If	0.045
28	1745352	1791963	ADAM metallopeptidase with thrombospondin type 1 motif, 10	0.045
28	1808517	1817532	Tyrosine-protein kinase	0.045
28	1846964	1848279	actin-like 9	0.045
28	1851800	1862161	mucin 16, cell surface associated	0.045
28	1930170	1932508	Ornithine decarboxylase antizyme 1	0.045
28	1956623	1960825	muellerian-inhibiting factor precursor	0.045
28	1962063	1966197	splicing factor 3A subunit 2	0.045
28	1967285	1973793	Pleckstrin homology domain-containing family J member 1	0.045
28	1978406	2033934	DOT1-like histone H3K79 methyltransferase	0.045
28	2607273	2609239	tubulin polyglutamylase complex subunit 1	0.055
28	2617593	2628605	Basigin	0.055
28	3651445	3653912	cytokine receptor-like factor 1	0.053
28	3657472	3659967	KxDL motif containing 1	0.053
28	3666228	3700318	RNA polymerase II elongation factor ELL	0.053
28	3728960	3735148	LSM4 homolog, U6 small nuclear RNA associated (S. cerevisiae)	0.053
28	3748373	3753211	phosphodiesterase 4C, cAMP-specific	0.053
28	3758472	3759385	MPV17 mitochondrial membrane protein-like 2	0.053
28	3759501	3762347	Interferon-gamma-inducible lysosomal thiol reductase	0.053
28	3782857	3800231	microtubule associated serine/threonine kinase 3	0.053
Z	9066147	9077155	tropomyosin beta chain	0.055
Z	9084930	9095262	carbonic anhydrase IX	0.055
Z	9141509	9155184	vav-like protein C9orf100 homolog	0.055

Z	9157781	9247668	collagen and calcium binding EGF domains 1	0.055
Z	9316120	9506598	PDZ domain containing 2	0.055
Z	9523777	9555867	golgi phosphoprotein 3 (coat-protein)	0.055
Z	9567397	9601423	myotubularin related protein 12	0.055
Z	9660188	9675175	Activated RNA polymerase II transcriptional coactivator p15	0.055
Z	9904567	9945803	natriuretic peptide receptor C/guanylate cyclase C (atrionatriuretic peptide receptor C)	0.055
Z	9298109	9298211	U6 spliceosomal RNA	0.055
Z	9639903	9640039	Small nucleolar RNA SNORA66	0.055
Z	12046216	12121536	RPTOR independent companion of MTOR, complex 2	0.050
Z	12129418	12192609	FYN-binding protein	0.050
Z	12205815	12230277	Dab, mitogen-responsive phosphoprotein, homolog 2 (Drosophila)	0.050
Z	15630610	15707360	integrin alpha-1 precursor	0.049
Z	15721124	15783248	integrin, alpha 2 (CD49B, alpha 2 subunit of VLA-2 receptor)	0.049
Z	15930691	15938408	Follistatin	0.049
Z	15962742	16010010	NADH dehydrogenase; Uncharacterized protein	0.049
Z	16062783	16259554	ADP-ribosylation factor-like 15	0.049
Z	16129627	16129795	TUC338	0.049
Z	16950760	17001487	ankyrin repeat domain 55	0.071
Z	44205924	44207300	nuclear factor interleukin-3-regulated protein	0.070
Z	55091689	55091845	TUC338	0.049
Z	63876645	63893410	dihydrofolate reductase	0.045
Z	68346670	68380646	intraflagellar transport 74 homolog (Chlamydomonas)	0.054
Z	68352558	68358247	leucine rich repeat containing 19	0.054
Z	68386224	68425628	TEK tyrosine kinase, endothelial	0.054
Z	81378368	81397400	FERM and PDZ domain containing 1	0.059
Z	81411705	81424704	DDB1 and CUL4 associated factor 10	0.059
