# Supplementary material for: Population Genomic Analyses Based on 1 Million SNPs in Commercial Egg Layers
Source: PLoS One. 2014 Apr 16;9(4):e94509. doi: 10.1371/journal.pone.0094509 (PMC3989219; doi:10.1371/journal.pone.0094509)
Supplement: Table S2 — List of genes in of upper 1% FST distribution in comparision of commercial layers and out-group. (PDF) [file pone.0094509.s002.pdf]

Table S2. List of genes in of upper 1% F_{ST} distribution in comparision of commercial layers and out-group.

Chr	Start	End	Description	Fst
1	9325096	9459193	semaphorin-3E precursor	0.261
1	9561909	9881531	piccolo presynaptic cytomatrix protein	0.261
1	15929586	15971452	ceramide kinase	0.242
1	15997492	16152675	TBC1 domain family member 22A	0.242
1	25847642	26191414	FoxP2; Uncharacterized protein	0.227
1	31999958	32082280	monocarboxylate transporter 2	0.230
1	35013708	35017857	Interferon gamma	0.203
1	35062146	35064598	interleukin-22 precursor	0.203
1	35074941	35098051	Mdm1 nuclear protein homolog (mouse)	0.203
1	35173230	35206537	Ras-related protein Rap-1b	0.203
1	35208345	35233387	nucleoporin 107kDa	0.203
1	35234300	35239841	solute carrier family 35 member E3	0.203
1	35255810	35267006	Mdm2, transformed 3T3 cell double minute 2, p53 binding protein	0.203
1	35273571	35305258	carboxypeptidase M precursor	0.203
1	35343616	35368673	Cleavage and polyadenylation specificity factor subunit 6	0.203
1	36575949	36791862	thyrotropin-releasing hormone degrading enzyme	0.213
1	43171637	43175978	dual specificity protein phosphatase 6	0.252
1	43207669	43254712	WD repeat-containing protein 51B	0.252
1	43273706	43305815	plasma membrane calcium-transporting ATPase 1	0.252
1	43798643	43821219	Epiphycan	0.252
1	43843145	43851618	Keratocan	0.252
1	43860883	43872867	Lumican	0.252
1	43894096	43932320	decorin precursor	0.252
1	44271338	44273899	Protein BTG1	0.252
1	44690009	44710912	nudix (nucleoside diphosphate linked moiety X)-type motif 4	0.252
1	44714689	44733745	ubiquitin-conjugating enzyme E2 N	0.252
1	44764153	44768210	suppressor of cytokine signaling 2	0.252

1	44775335	44851153	death domain-containing protein CRADD	0.252
1	45179843	45187320	NADH dehydrogenase	0.252
1	45195370	45239814	nuclear receptor subfamily 2 group C member 1	0.252
1	45245468	45308710	FYVE, RhoGEF and PH domain containing 6	0.252
1	45406753	45421533	methionine aminopeptidase 2	0.252
1	45425569	45437573	Ubiquitin carboxyl-terminal hydrolase	0.252
1	46488737	46510905	lamina-associated polypeptide 2, isoform beta	0.265
1	46514910	46522801	phosphate carrier protein, mitochondrial	0.265
1	46530982	46536438	IKBKB interacting protein	0.265
1	46520990	46521230	Small nucleolar RNA SNORA53	0.265
1	49977775	50105059	trinucleotide repeat containing 6B	0.219
1	50154190	50196801	GRB2-related adaptor protein 2	0.219
1	50448547	50450694	active regulator of SIRT1	0.219
1	50456693	50459626	cyclic AMP-dependent transcription factor ATF-4	0.219
1	50517363	50525791	TGF-beta-activated kinase 1 and MAP3K7-binding protein 1	0.219
1	50534583	50544913	synaptogyrin 1	0.219
1	50548764	50554568	60S ribosomal protein L3	0.219
1	50582287	50594411	platelet-derived growth factor subunit B precursor	0.219
1	50609571	50619165	chromobox homolog 7	0.219
1	50490804	50492294	beta-1,4-mannosyl-glycoprotein 4-beta-N-acetylglucosaminyltransferase	0.219
1	50552408	50552500	Small nucleolar RNA U83B	0.219
1	50354165	50354490	7SK RNA	0.219
1	51759800	51835231	RNA binding protein, fox-1 homolog (C. elegans) 2	0.211
1	51849854	51853554	Myoglobin	0.211
1	51939574	51947830	DNA replication licensing factor MCM5	0.211
1	51949101	51955226	heme oxygenase 1	0.211
1	51962485	51983162	Target of Myb protein 1	0.211
1	54494944	54562153	Solute carrier family 41 member 2	0.213
1	55745325	55820855	myosin binding protein C, slow type	0.198
1	58356941	58391611	myotrophin	0.205

1	58482771	58513575	family with sequence similarity 180, member A	0.205
1	60698479	60974728	ELKS/RAB6-interacting/CAST family member 1	0.219
1	61044205	61059847	protein Wnt-5b precursor	0.219
1	61067944	61110429	adiponectin receptor protein 2	0.219
1	61241662	61287347	decapping mRNA 1B	0.219
1	61121260	61123185	CHUNK-1 protein	0.219
1	62933511	63081609	receptor-type tyrosine-protein phosphatase O	0.199
1	63087279	63140780	epidermal growth factor receptor pathway substrate 8	0.199
1	63250603	63259735	Serine-threonine kinase receptor-associated protein	0.199
1	63261392	63316947	putative deoxyribose-phosphate aldolase	0.199
1	63429485	63435977	microsomal glutathione S-transferase 1	0.199
1	66068227	66322847	transcription factor SOX-5	0.250
1	68357089	68442009	liprin-beta-1	0.292
1	68516162	68549653	ADP-ribosylation factor GTPase-activating protein 3	0.292
1	68556334	68619317	protein kinase C and casein kinase substrate in neurons protein 2 isoform 1	0.292
1	68626823	68645266	tubulin tyrosine ligase-like family, member 1	0.292
1	68667862	68672968	malonyl CoA:ACP acyltransferase (mitochondrial)	0.292
1	68683782	68704548	tubulin--tyrosine ligase-like protein 12	0.292
1	68715574	68928375	signal peptide, CUB domain, EGF-like 1	0.292
1	69162384	69195805	sulfotransferase family 4A, member 1	0.292
1	69327839	69350968	parvin, gamma	0.292
1	68833800	68833975	TUC338	0.292
1	70098157	70154426	PHD finger protein 21B	0.215
1	72902806	73148067	fatty acyl CoA reductase 2	0.224
1	76247535	76251880	cystatin B (stefin B)	0.233
1	76253583	76258549	cystatin A (stefin A)	0.233
1	76278352	76285046	family with sequence similarity 162, member A	0.233
1	76288629	76339983	Importin subunit alpha-1	0.233
1	76360081	76368699	tapasin-related protein	0.233
1	76382783	76390957	amiloride-sensitive sodium channel subunit alpha	0.233

1	76396666	76398676	vesicle-associated membrane protein 1 (synaptobrevin 1)	0.233
1	76399984	76401537	39S ribosomal protein L51, mitochondrial	0.233
1	76401717	76426364	non-SMC condensin I complex, subunit D2	0.233
1	76430011	76431726	C-type natriuretic peptide 1 precursor	0.233
1	76434647	76437498	Glyceraldehyde-3-phosphate dehydrogenase	0.233
1	76463548	76468438	NOP2 nucleolar protein	0.233
1	76468778	76489284	chromodomain helicase DNA binding protein 4	0.233
1	76507821	76515304	lysophosphatidic acid receptor 5	0.233
1	76520460	76539139	acrosin binding protein	0.233
1	76548297	76563040	Inhibitor of growth protein 4	0.233
1	76630627	76633759	COP9 signalosome complex subunit 7a	0.233
1	76644865	76651986	myeloid leukemia factor 2	0.233
1	76713681	76725911	leprecan-like 2	0.233
1	76738644	76753824	ubiquitin specific peptidase 5 (isopeptidase T)	0.233
1	76754054	76757054	Triosephosphate isomerase	0.233
1	76766963	76776197	gamma-enolase	0.233
1	76472752	76472892	Small Cajal body specific RNA 11	0.233
1	76402298	76402618	Small nucleolar RNA U85	0.233
1	78926395	79112873	syntaxin binding protein 5-like	0.197
1	84389704	84521596	ABI family, member 3 (NESH) binding protein	0.223
1	84610640	84660808	interphotoreceptor matrix proteoglycan 2 precursor	0.223
1	84665305	84703930	SUMO1/sentrin specific peptidase 7	0.223
1	84714811	84724364	PEST proteolytic signal-containing nuclear protein	0.223
1	84747544	84751604	ribosomal protein L24	0.223
1	84799655	84816004	NF-kappa-B inhibitor zeta	0.223
1	85893284	86010821	CD166 antigen	0.223
1	86037078	86159219	Cbl proto-oncogene B, E3 ubiquitin protein ligase	0.223
1	91358735	91424437	prostaglandin F2 receptor inhibitor	0.208
1	91431223	91441019	CD101 molecule	0.208
1	1.08E+08	1.08E+08	Down syndrome cell adhesion molecule	0.227

1	1.09E+08	1.09E+08	radial spoke head 1 homolog (Chlamydomonas)	0.207
1	1.09E+08	1.09E+08	solute carrier family 37 (glucose-6-phosphate transporter), member 1	0.207
1	1.09E+08	1.09E+08	phosphodiesterase 9A	0.207
1	1.09E+08	1.09E+08	NADH dehydrogenase	0.207
1	1.17E+08	1.17E+08	DNA polymerase	0.283
1	1.17E+08	1.18E+08	phosphate cytidylyltransferase 1, choline, beta	0.283
1	1.17E+08	1.17E+08	U1 spliceosomal RNA	0.283
1	1.17E+08	1.17E+08	Small Cajal body specific RNA 24	0.283
1	1.3E+08	1.31E+08	gamma-aminobutyric acid (GABA) A receptor, gamma 3	0.210
1	1.31E+08	1.31E+08	Gamma-aminobutyric acid receptor subunit beta-3	0.210
1	1.36E+08	1.36E+08	ras GTPase-activating protein 3	0.255
1	1.44E+08	1.44E+08	dedicator of cytokinesis 9	0.224
1	1.45E+08	1.46E+08	multidrug resistance-associated protein 4	0.230
1	1.47E+08	1.47E+08	glypican 5	0.204
1	1.49E+08	1.49E+08	SLIT and NTRK-like family, member 5	0.264
1	1.52E+08	1.52E+08	protein sprouty homolog 2	0.267
1	1.55E+08	1.55E+08	progesterone immunomodulatory binding factor 1	0.202
1	1.62E+08	1.62E+08	tudor domain-containing protein 3	0.230
1	1.65E+08	1.65E+08	ETS-related transcription factor Elf-1	0.261
1	1.65E+08	1.65E+08	WW domain-binding protein 4	0.261
1	1.65E+08	1.65E+08	N(alpha)-acetyltransferase 16, NatA auxiliary subunit	0.261
1	1.65E+08	1.65E+08	regulator of cell cycle	0.261
1	1.65E+08	1.65E+08	von Willebrand factor A domain containing 8	0.261
1	1.66E+08	1.66E+08	diacylglycerol kinase, eta	0.261
1	1.68E+08	1.68E+08	esterase D	0.215
1	1.68E+08	1.68E+08	5-hydroxytryptamine (serotonin) receptor 2A, G protein-coupled	0.215
1	1.68E+08	1.68E+08	succinate-CoA ligase, ADP-forming, beta subunit	0.215
1	1.68E+08	1.68E+08	integral membrane protein 2B	0.215
1	1.68E+08	1.68E+08	retinoblastoma-associated protein	0.215
1	1.68E+08	1.68E+08	regulator of chromosome condensation (RCC1) and BTB (POZ) domain containing protein 2	0.215

1	1.68E+08	1.68E+08	fibronectin type-III domain-containing protein 3a	0.215
1	1.68E+08	1.68E+08	motilin receptor	0.215
1	1.68E+08	1.69E+08	calcium binding protein 39-like	0.215
1	1.74E+08	1.74E+08	sister chromatid cohesion protein PDS5 homolog B	0.243
1	1.74E+08	1.74E+08	phosphonoformate immuno-associated protein 5	0.243
1	1.74E+08	1.74E+08	breast cancer 2, early onset	0.243
1	1.74E+08	1.74E+08	ZAR1-like protein	0.243
1	1.74E+08	1.74E+08	furry homolog (Drosophila)	0.243
1	1.77E+08	1.77E+08	spastic ataxia of Charlevoix-Saguenay (sacsin)	0.225
1	1.78E+08	1.78E+08	glia-activating factor	0.225
1	1.78E+08	1.78E+08	Paraspeckle component 1	0.223
1	1.78E+08	1.78E+08	M-phase phosphoprotein 8	0.223
1	1.79E+08	1.79E+08	ring finger protein 17	0.223
1	1.8E+08	1.8E+08	serine-protein kinase ATM	0.207
1	1.8E+08	1.8E+08	acetyl-CoA acetyltransferase 1	0.207
1	1.8E+08	1.8E+08	cullin 5	0.207
1	1.82E+08	1.82E+08	transient receptor potential cation channel, subfamily C, member 6	0.202
1	1.84E+08	1.84E+08	Myotubularin-related protein 2	0.226
1	1.93E+08	1.93E+08	erythroblast NAD(P)(+)--arginine ADP-ribosyltransferase precursor	0.195
1	1.94E+08	1.94E+08	glycerophosphodiester phosphodiesterase domain-containing protein 5	0.196
2	13068736	13498548	partitioning defective 3 homolog	0.234
2	13816140	13922505	neuropilin-1 precursor	0.234
2	13975831	14017232	integrin beta-1 precursor	0.234
2	13771061	13771230	TUC338	0.234
2	14597995	14657468	supervillin	0.209
2	14932503	14950908	mitogen-activated protein kinase kinase kinase 8	0.209
2	15410320	15499606	membrane protein, palmitoylated 7 (MAGUK p55 subfamily member 7)	0.201
2	18960948	18977002	ADP-ribosylation factor-like 5B	0.262
2	19567820	19575886	protein tyrosine phosphatase-like (proline instead of catalytic arginine), member A	0.262
2	19614633	19653024	alpha-2,8-sialyltransferase 8F	0.262

2	19677910	19684966	vimentin	0.262
2	19737380	19876287	cubilin (intrinsic factor-cobalamin receptor)	0.262
2	19881822	19979217	ras suppressor protein 1	0.262
2	26168484	26327087	PHD finger protein 14	0.252
2	31286666	31393727	Insulin-like growth factor 2 mRNA-binding protein 3	0.203
2	41036173	41058663	Cytoplasmic dynein 1 light intermediate chain 1	0.251
2	41080291	41109486	CCR4-NOT transcription complex subunit 10	0.251
2	41117521	41127915	E3 ubiquitin-protein ligase TRIM71	0.251
2	45255770	45389575	cytoplasmic linker associated protein 2	0.210
2	50484818	50543299	cyclin-dependent kinase 13	0.205
2	50564486	50571264	M-phase specific PLK1 interacting protein	0.205
2	66581522	66713653	exocyst complex component 2	0.200
2	67868597	67888199	vacuolar protein sorting-associated protein 4B	0.194
2	67898884	67917107	3-ketodihydrosphingosine reductase	0.194
2	79163255	79192329	5-methyltetrahydrofolate-homocysteine methyltransferase reductase	0.315
2	79193761	79203732	FAST kinase domains 3	0.315
2	79220949	79424359	adenylate cyclase 2 (brain)	0.315
2	81014599	81124708	cordon-bleu WH2 repeat protein	0.216
2	83099424	83462516	formin homology 2 domain containing 3	0.235
2	83451751	83451909	TUC338	0.235
2	86624614	86631727	iroquois-class homeodomain protein IRX-2	0.253
2	88558023	88560120	protein FAM206A	0.220
2	1.03E+08	1.03E+08	RIO kinase 3	0.220
2	1.03E+08	1.03E+08	Niemann-Pick disease, type C1	0.220
2	1.17E+08	1.17E+08	telomeric repeat-binding factor 1	0.198
2	1.23E+08	1.23E+08	cyclic nucleotide gated channel beta 3	0.196
2	1.23E+08	1.24E+08	matrix metalloproteinase-16	0.200
2	1.29E+08	1.3E+08	regulating synaptic membrane exocytosis 2	0.229
2	1.3E+08	1.3E+08	low density lipoprotein receptor-related protein 12	0.229
2	1.36E+08	1.36E+08	sterile alpha motif domain containing 12	0.212

2	1.36E+08	1.36E+08	tumor necrosis factor receptor superfamily member 11B precursor	0.212
2	1.36E+08	1.36E+08	Collectin-10	0.212
2	1.36E+08	1.36E+08	protein MAL2	0.212
2	1.36E+08	1.36E+08	protein NOV precursor	0.212
2	1.36E+08	1.36E+08	ectonucleotide pyrophosphatase/phosphodiesterase family member 2	0.212
2	1.36E+08	1.36E+08	TAF2 RNA polymerase II, TATA box binding protein (TBP)-associated factor, 150kDa	0.212
2	1.37E+08	1.37E+08	hyaluronan synthase 2	0.224
3	6450555	7094776	neurexin-1-alpha isoform 1 precursor	0.256
3	7268194	7268297	U6 spliceosomal RNA	0.256
3	9521292	9539167	protein pellino homolog 1	0.204
3	14702016	14714699	D-tyrosyl-tRNA(Tyr) deacylase 1	0.217
3	14716777	14733389	Protein transport protein Sec23A	0.217
3	20356020	20391564	centromere protein F	0.237
3	20511715	20538253	N-lysine methyltransferase SMYD2	0.237
3	21504436	21511540	serine/threonine-protein kinase Nek2	0.245
3	21605815	21621949	TNF receptor-associated factor 5	0.245
3	21628366	21650478	REST corepressor 3	0.245
3	26287945	26291968	multiple coagulation factor deficiency 2 precursor	0.226
3	26292215	26454063	tetratricopeptide repeat protein 7A	0.226
3	26488768	26495658	Calmodulin	0.226
3	26566431	26596265	Serine/threonine-protein phosphatase PP1-beta catalytic subunit	0.226
3	26632539	26715148	phospholipase B1	0.226
3	26737453	26743407	fos-related antigen 2	0.226
3	26766303	26929435	BRCA1-A complex subunit BRE	0.226
3	28093815	28285437	dishevelled associated activator of morphogenesis 2	0.273
3	28650945	28730284	glucagon-like peptide 1 receptor precursor	0.273
3	28737986	28830043	dynein, axonemal, heavy chain 8	0.273
3	29519203	29541046	FtsJ methyltransferase domain containing 2	0.206
3	29557966	29574363	probable alanyl-tRNA synthetase, mitochondrial	0.206
3	29611732	29620288	nuclear factor of kappa light polypeptide gene enhancer in B-cells inhibitor, epsilon	0.206

3	29630922	29636756	heat shock cognate protein HSP 90-beta	0.206
3	34322339	34455636	v-akt murine thymoma viral oncogene homolog 3	0.198
3	34464087	34563969	serologically defined colon cancer antigen 8	0.198
3	36498791	36661068	ryanodine receptor 2 (cardiac)	0.210
3	41393868	41474285	myeloid/lymphoid or mixed-lineage leukemia (trithorax homolog, Drosophila)	0.275
3	41687479	41708127	unc-93 homolog A (C. elegans)	0.275
3	41711697	41713292	tubulin tyrosine ligase-like family, member 2	0.275
3	41759132	41766064	C-C chemokine receptor type 6	0.275
3	41783071	41803951	FGFR1 oncogene partner	0.275
3	41810099	41831299	ribonuclease T2 precursor	0.275
3	41958446	42114412	ribosomal protein S6 kinase, 90kDa, polypeptide 2	0.275
3	42131326	42141404	brain protein 44-like protein	0.275
3	42233586	42241528	brachyury protein	0.275
3	42463951	42626252	phosphodiesterase 10A	0.275
3	43765827	44445298	parkinson protein 2, E3 ubiquitin protein ligase (parkin)	0.200
3	45215648	45566922	utrophin	0.217
3	45769623	45811385	epilepsy, progressive myoclonus type 2A, Lafora disease (laforin)	0.217
3	45868769	45921436	SNF2 histone linker PHD RING helicase, E3 ubiquitin protein ligase	0.217
3	45937157	46118743	Glutamate receptor metabotropic 1 isoform f transcript variant 1	0.217
3	46182574	46256838	androglobin	0.217
3	46388675	46489008	syntaxin binding protein 5 (tomosyn)	0.217
3	46522593	46647011	sterile alpha motif domain containing 5	0.217
3	47394890	47423136	peptidylprolyl isomerase (cyclophilin)-like 4	0.220
3	47425648	47442269	uncharacterized protein C6orf72 homolog precursor	0.220
3	47446107	47459848	Katanin p60 ATPase-containing subunit A1	0.220
3	48276571	48431441	estrogen receptor	0.216
3	48447032	48720659	spectrin repeat containing, nuclear envelope 1	0.216
3	48776284	48795822	VIP peptides isoform 2 preproprotein	0.216
3	48910153	48981998	Regulator of G-protein signaling 17	0.216
3	50465764	50754522	AT rich interactive domain 1B (SWI1-like)	0.243

3	73318964	73462488	Ephrin type-A receptor 7	0.236
3	75787755	75824704	origin recognition complex subunit 3	0.203
3	75858402	75874162	CMP-sialic acid transporter	0.203
3	79966253	79995357	SUMO1/sentrin specific peptidase 6	0.211
3	80071280	80164378	filamin A interacting protein 1	0.211
3	80180051	80193545	cell cycle control protein 50A	0.211
3	80197965	80201301	cytochrome c oxidase subunit 7A2, mitochondrial	0.211
3	80212182	80312375	collagen alpha-1(XII) chain precursor	0.211
3	81337238	81638081	regulating synaptic membrane exocytosis 1	0.195
3	86249530	86545594	dystonin	0.227
3	86766922	86825286	bone morphogenetic protein 5 precursor	0.227
3	88949756	89752149	CUB and Sushi multiple domains 1	0.228
3	96291158	96342936	Hippocalcin-like protein 1	0.268
3	96350213	96356450	ornithine decarboxylase	0.268
3	96480139	96496443	ATPase, H+ transporting, lysosomal 42kDa, V1 subunit C2	0.268
3	96729661	96737554	PQ loop repeat containing 3	0.268
3	96746492	96802174	Rho-associated, coiled-coil containing protein kinase 2	0.268
3	96852537	96861622	transcription factor E2F6	0.268
3	96906122	96965158	growth regulation by estrogen in breast cancer 1	0.268
3	96971362	97044067	Lipin 1; Uncharacterized protein	0.268
3	97427885	97448910	tribbles homolog 2	0.268
3	98343307	98504175	neuroblastoma amplified sequence	0.268
3	98505120	98526381	ATP-dependent RNA helicase DDX1	0.268
3	98778414	98780948	N-myc proto-oncogene protein	0.268
3	99136709	99173961	Protein FAM49A	0.268
3	1.04E+08	1.05E+08	putative Polycomb group protein ASXL2	0.212
3	1.09E+08	1.09E+08	cytochrome P450, family 39, subfamily A, polypeptide 1	0.216
3	1.09E+08	1.09E+08	tudor domain containing 6	0.216
3	1.09E+08	1.09E+08	platelet-activating factor acetylhydrolase	0.216
3	1.1E+08	1.1E+08	G protein-coupled receptor 116	0.216

3	1.1E+08	1.1E+08	tumor necrosis factor receptor superfamily member 21 precursor	0.216
3	1.1E+08	1.1E+08	CD2-associated protein	0.216
3	1.09E+08	1.09E+08	taste receptor type 2 member 7	0.216
4	10450054	10450218	TUC338	0.217
4	17161323	17256387	Nik related kinase	0.214
4	22824459	22828895	neuropeptide Y receptor type 1	0.196
4	34202352	34367249	Fraser syndrome 1	0.299
4	34415667	34425383	CCR4-NOT transcription complex, subunit 6-like	0.299
4	34433792	34435418	Chemokine	0.299
4	34460742	34467515	cyclin G2	0.299
4	34487202	34488289	PIGY upstream reading frame	0.299
4	34496081	34542198	probable E3 ubiquitin-protein ligase HERC3	0.299
4	34542533	34656257	family with sequence similarity 13, member A	0.299
4	34818548	34885635	alpha-synuclein	0.299
4	34906759	34949844	multimerin 1	0.299
4	34693911	34696151	GPRIN family member 3	0.299
4	37847835	37861897	aminoacyl tRNA synthetase complex-interacting multifunctional protein 1	0.227
4	37878922	37971616	TBC domain-containing protein kinase-like protein	0.227
4	37998529	38044094	nephronectin	0.227
4	38124523	38140681	integrator complex subunit 12	0.227
4	38146410	38172500	Rho guanine nucleotide exchange factor (GEF) 38	0.227
4	38883037	38919985	long-chain-fatty-acid--CoA ligase 1	0.209
4	38921548	38931422	centromere protein U	0.209
4	42203702	42635333	UDP-N-acetyl-alpha-D-galactosamine:polypeptide N-acetylgalactosaminyltransferase-like 6	0.224
4	53397253	53412498	annexin A5	0.202
4	53488372	53510295	pyroglutamylated RFamide peptide receptor	0.202
4	53544222	53582180	TNFAIP3 interacting protein 3	0.202
4	53644937	53729832	PR domain containing 5	0.202
4	53693495	53693674	TUC338	0.202
4	59910288	60049717	protein phosphatase 3, catalytic subunit, alpha isozyme	0.218

4	61227165	61276241	Melatonin receptor type 1A	0.260
4	63228829	63342046	tumor suppressor candidate 3	0.230
4	63469831	63819243	sarcoglycan, zeta	0.230
4	68691120	68764869	sister chromatid cohesion protein PDS5 homolog A	0.226
4	69522430	69572232	KIAA1239	0.213
4	69781392	69796531	death domain containing 1	0.213
4	69799893	69923066	ArfGAP with RhoGAP domain, ankyrin repeat and PH domain 2	0.213
4	75401346	75452145	Ligand-dependent nuclear receptor corepressor-like protein	0.196
4	75480300	75503309	non-SMC condensin I complex, subunit G	0.196
4	81699702	81774162	huntingtin	0.226
4	81780294	81810730	G protein-coupled receptor kinase 4	0.226
4	81861191	81918087	alpha-adducin	0.226
4	81924388	81959922	SH3 domain-binding protein 2	0.226
4	82006422	82013592	TNFAIP3-interacting protein 2	0.226
4	82049672	82097810	family with sequence similarity 193, member A	0.226
4	81902213	81902370	U1 spliceosomal RNA	0.226
5	6774141	6845804	PTPRF interacting protein, binding protein 2 (liprin beta 2)	0.224
5	6836663	6856264	NADH-cytochrome b5 reductase 2	0.224
5	8301559	8330255	murine retrovirus integration site 1 homolog	0.229
5	8332204	8342809	lymphatic vessel endothelial hyaluronic acid receptor 1	0.229
5	8344527	8355772	RING finger protein 141	0.229
5	8361714	8387323	AMP deaminase 3	0.229
5	8868538	8920092	DENN/MADD domain containing 5A	0.198
5	8927057	8961764	signal peptide, CUB domain, EGF-like 2	0.198
5	12238926	12267404	receptor-type tyrosine-protein phosphatase eta	0.234
5	22080490	22083151	family with sequence similarity 180, member B	0.238
5	22088781	22093418	NADH dehydrogenase	0.238
5	22095231	22100669	kelch repeat and BTB domain-containing protein 4	0.238
5	22100815	22102871	protein tyrosine phosphatase, mitochondrial 1	0.238
5	22103005	22150651	CUGBP Elav-like family member 1	0.238

5	22076422	22077558	C1q and tumor necrosis factor related protein 4	0.238
5	22947434	22948426	midkine precursor	0.218
5	22949353	22992334	diacylglycerol kinase zeta	0.218
5	22944471	22945943	muscarinic acetylcholine receptor M4	0.218
5	23959882	23993960	inositol-trisphosphate 3-kinase A	0.257
5	24139477	24176035	RNA polymerase II associated protein 1	0.257
5	24215769	24250023	tyrosine-protein kinase receptor TYRO3 precursor	0.257
5	24268925	24313980	MGA, MAX dimerization protein	0.257
5	24330575	24419530	mitogen-activated protein kinase binding protein 1	0.257
5	24424421	24430668	jmjC domain-containing protein 7	0.257
5	24487476	24583608	spectrin, beta, non-erythrocytic 5	0.257
5	24595101	24618120	EH-domain containing 4	0.257
5	24865079	24890722	vam6/Vps39-like protein	0.257
5	24915661	24936853	neutral alpha-glucosidase C	0.257
5	24937025	24960748	calpain-3	0.257
5	25000359	25015379	synaptosomal-associated protein 23	0.257
5	26489235	26559259	pecanex homolog (Drosophila)	0.221
5	33101816	33216460	serine/threonine-protein kinase D1	0.201
5	36015953	36247167	solute carrier family 25 (mitochondrial oxoadipate carrier), member 21	0.217
5	36843282	36865890	Protein transport protein Sec23A	0.217
5	36871372	36880109	survival of motor neuron protein-interacting protein 1	0.217
5	36880151	36883970	trafficking protein particle complex 6B	0.217
5	36884690	36894010	pinin, desmosome associated protein	0.217
5	36989049	37003244	Ectonucleoside triphosphate diphosphohydrolase 5	0.217
5	37025663	37064969	Protein lin-52 homolog	0.217
5	36632383	36633663	somatostatin receptor type 1	0.217
5	42319730	42344911	spermatogenesis associated 7	0.200
5	42427791	42521303	echinoderm microtubule associated protein like 5	0.200
5	43694395	43711430	tandem C2 domains, nuclear	0.218
5	43832919	43844552	Ataxin-3	0.218

5	43845120	43847133	NADH dehydrogenase	0.218
5	43847339	43860849	cleavage and polyadenylation specificity factor subunit 2	0.218
5	45876530	45909816	serine/threonine-protein kinase VRK1	0.284
5	47783078	47906765	Histone-lysine N-methyltransferase setd3	0.281
5	47907119	47919817	cyclin-K	0.281
5	48046014	48063255	HHIP-like 1	0.281
5	48067831	48083159	cholesterol 24-hydroxylase	0.281
5	48159648	48241305	echinoderm microtubule associated protein like 1	0.281
5	48312771	48378821	ena/VASP-like protein	0.281
5	48418077	48431424	transcriptional repressor protein YY1	0.281
5	48456828	48465791	solute carrier family 25, member 47	0.281
5	48490625	48546715	WD repeat domain 25	0.281
5	48566385	48715173	brain-enriched guanylate kinase-associated	0.281
5	48844046	48853652	protein delta homolog 1 precursor	0.281
5	53874253	53882060	small nuclear RNA activating complex, polypeptide 1, 43kDa	0.220
5	53884053	53914606	hypoxia-inducible factor 1-alpha	0.220
6	11556862	11568617	Histone H2A	0.217
6	20754954	20843012	DNA nucleotidylexotransferase	0.266
6	21051932	21053666	VENT homeobox	0.266
6	21074071	21135637	kinase non-catalytic C-lobe domain (KIND) containing 1	0.266
6	27087946	27102495	DNA cross-link repair 1A protein	0.202
6	27103400	27134851	NHL repeat-containing protein 2	0.202
6	31243849	31257991	G protein-coupled receptor 26	0.249
6	31382330	31425438	carbohydrate (N-acetylgalactosamine 4-sulfate 6-O) sulfotransferase 15	0.249
6	31539142	31551526	ornithine aminotransferase, mitochondrial	0.249
6	31560638	31561609	NK1 homeobox 2	0.249
6	31636707	31661235	family with sequence similarity 53, member B	0.249
6	31686516	31702105	BRISC complex subunit Abro1	0.249
7	5508762	5538667	transient receptor potential cation channel subfamily M member 8	0.212
7	5546056	5553781	secreted phosphoprotein 24	0.212

7	5654088	5654666	ADP-ribosylation factor-like 4C	0.212
7	10900361	10919427	CASP8 and FADD-like apoptosis regulator	0.200
7	10924385	10932701	caspase 10, apoptosis-related cysteine peptidase	0.200
7	10936137	10944290	caspase 18	0.200
7	10949123	10955503	caspase-8	0.200
7	10990622	10997507	STE20-related kinase adaptor beta	0.200
7	11017902	11042872	membrane protein, palmitoylated 4 (MAGUK p55 subfamily member 4)	0.200
7	11084221	11117761	cyclin-dependent kinase 15	0.200
7	11182131	11185182	frizzled-7 precursor	0.200
7	16313906	16361739	WAS/WASL-interacting protein family member 1	0.206
7	17214612	17252865	integrin alpha-6 precursor	0.223
7	18074706	18082768	Sjogren syndrome antigen B (autoantigen La) isoform 1	0.203
7	18115845	18140425	peptidylprolyl isomerase G (cyclophilin G)	0.203
7	18171747	18181640	Bardet-Biedl syndrome 5 protein	0.203
7	18254209	18342504	low density lipoprotein receptor-related protein 2	0.203
7	18366761	18411191	ATP-binding cassette, sub-family B (MDR/TAP), member 11	0.203
7	18583602	18668725	serine threonine kinase 39	0.203
7	26674970	26859021	myosin light chain kinase, smooth muscle	0.207
7	30459507	30701723	thrombospondin, type I, domain containing 7B	0.209
7	31350097	31932431	low density lipoprotein receptor-related protein 1	0.195
8	822750	960453	netrin G1	0.247
8	987129	1134223	guanine nucleotide exchange factor VAV3	0.247
8	1182030	1195519	solute carrier family 25 (mitochondrial carrier; phosphate carrier), member 24	0.247
8	1233367	1239025	Small RNA 2'-O-methyltransferase	0.247
8	1264534	1289487	syntaxin-binding protein 3	0.247
8	1303357	1327945	G-protein signaling modulator 2	0.247
8	1362791	1578757	calmodulin regulated spectrin-associated protein family, member 2	0.247
8	1448262	1477082	kinesin family member 14	0.247
8	1585806	1668861	nuclear receptor subfamily 5 group A member 2	0.247
8	2019148	2076592	receptor-type tyrosine-protein phosphatase C precursor	0.247

8	3981797	3985419	cysteine-rich protein 2	0.204
8	3995482	4052393	metastasis-associated protein MTA1	0.204
8	4130593	4139427	Vesicle-trafficking protein SEC22b	0.204
8	4145809	4225658	neurogenic locus notch homolog protein 2 precursor	0.204
8	4229378	4233158	3-hydroxy-3-methylglutaryl-CoA synthase 2 (mitochondrial)	0.204
8	4233645	4236981	phosphoglycerate dehydrogenase	0.204
8	4241111	4268884	phosphodiesterase 4D interacting protein	0.204
8	4269072	4275923	5'-AMP-activated protein kinase subunit beta-2	0.204
8	4284025	4287967	phosphatidic acid phosphatase type 2 domain containing 2	0.204
8	4296031	4302926	peroxiredoxin-6	0.204
8	4303065	4316854	transcriptional adaptor 1 (HFI1 homolog, yeast)-like	0.204
8	5890359	5957040	acyl-CoA binding domain containing 6	0.221
8	10722448	10772172	CDC14 cell division cycle 14 homolog A	0.243
8	10783844	10785051	G protein-coupled receptor 88	0.243
8	10837704	10851191	RNA 3'-terminal phosphate cyclase	0.243
8	10851933	10864943	lipoamide acyltransferase component of branched-chain alpha-keto acid dehydrogenase complex	0.243
8	10883574	10895986	spindle assembly abnormal protein 6 homolog	0.243
8	10918535	10935926	UDP-N-acetylglucosamine transporter	0.243
8	11017203	11051067	amylo-alpha-1, 6-glucosidase, 4-alpha-glucanotransferase	0.243
8	11071107	11128777	palmdelphin	0.243
8	14118086	14145849	protein kinase N2	0.271
8	27103615	27128984	tyrosine-protein kinase JAK1	0.200
9	1075334	1241140	Heparan-sulfate 6-O-sulfotransferase 1	0.209
9	4978758	5005060	period circadian protein homolog 2	0.238
9	5092108	5175814	phosphatidylinositol-4,5-bisphosphate 3-kinase catalytic subunit beta isoform	0.238
9	5411909	5436836	retinol binding protein 1, cellular	0.238
9	5566977	5861817	calsyntenin 2	0.238
9	5200075	5200992	forkhead box protein L2	0.238
9	12596242	12623350	ATPase type 13A5	0.218
9	12628993	12700392	zonadhesin	0.218

9	12741369	12791772	Mab-21 domain containing 2	0.218
9	12913816	12999648	fibroblast growth factor 12	0.218
9	13178354	13185135	urotensin-2B precursor	0.218
9	13186472	13199826	osteocrin precursor	0.218
9	13337288	13367409	interleukin 1 receptor accessory protein	0.218
9	13417660	13427011	claudin-1	0.218
9	13458582	13513540	prolyl 3-hydroxylase 2 precursor	0.218
9	13522136	13675334	tumor protein 63	0.218
9	16565596	16587667	fragile X mental retardation syndrome-related protein 1	0.236
9	16663283	16672397	tetratricopeptide repeat protein 14	0.236
9	16760382	16850020	peroxisomal biogenesis factor 5-like	0.236
9	16861502	16902461	Ubiquitin carboxyl-terminal hydrolase 13	0.236
9	16903953	16907474	NADH dehydrogenase	0.236
9	16911951	16921482	actin-like 6A	0.236
9	16984938	17003984	mitofusin-1	0.236
9	16342563	16343510	Transcription factor SOX-2	0.236
9	16352844	16353105	SOX2 overlapping transcript exon 3	0.236
9	16391649	16391874	SOX2 overlapping transcript exon 1	0.236
9	16820695	16820798	U6 spliceosomal RNA	0.236
9	16340729	16340829	SOX2 overlapping transcript exon 4	0.236
9	18196758	18471834	neuroligin-1	0.194
9	20419761	20464371	cholinesterase precursor	0.198
9	23105804	23118255	eukaryotic translation initiation factor 2A	0.243
9	23119142	23147022	TSC22 domain family, member 2	0.243
9	23218003	23220395	profilin-2	0.243
9	23226057	23254253	E3 ubiquitin-protein ligase RNF13	0.243
9	23254409	23259438	COMM domain containing 2	0.243
9	23264511	23304671	WW domain containing transcription regulator 1	0.243
9	23359472	23377519	ceruloplasmin (ferroxidase)	0.243
9	23410228	23425241	glycogenin-1	0.243

10	3952464	4034731	vacuolar protein sorting 13 homolog C (S. cerevisiae)	0.196
10	4876780	4886390	BCL2/adenovirus E1B 19kDa interacting protein 2	0.237
10	4892733	4897305	transcription initiation factor IIA subunit 2	0.237
10	5072623	5098236	Kruppel-like factor 13	0.237
10	5146745	5227651	transient receptor potential cation channel, subfamily M, member 1	0.237
10	5235131	5266963	myotubularin related protein 10	0.237
10	5299493	5303542	methylmalonyl CoA epimerase	0.237
10	5396943	5466890	family with sequence similarity 189, member A1	0.237
10	5524064	5593805	tight junction protein 1	0.237
10	6434758	6619897	disintegrin and metalloproteinase domain-containing protein 10 precursor	0.244
10	6480269	6521283	lipase, hepatic	0.244
10	6572777	6596733	aquaporin 9	0.244
10	6604818	6661741	Retinal dehydrogenase 2	0.244
10	6816944	6857906	cingulin-like 1	0.244
10	6903270	7058903	transcription factor 12	0.244
10	7158297	7164937	meiosis-specific nuclear structural 1	0.244
10	7159553	7183472	testis expressed 9	0.244
10	7249251	7256715	regulatory factor X, 7	0.244
10	7266832	7316503	E3 ubiquitin-protein ligase	0.244
10	7329784	7416677	protogenin precursor	0.244
10	7427934	7435865	pygopus homolog 1	0.244
10	7444550	7452475	dyslexia susceptibility 1 candidate 1	0.244
10	7454665	7473825	Cell cycle progression protein 1	0.244
10	7470471	7480656	phosphatidylinositol glycan anchor biosynthesis, class B	0.244
10	7484146	7513189	ras-related protein Rab-27A	0.244
10	7641038	7759221	unc-13 homolog C (C. elegans)	0.244
10	7807793	7874460	WD repeat domain 72	0.244
10	8193075	8224905	Protein FAM214A	0.244
10	11740664	11788096	KIAA1199	0.214
10	16601477	16763501	ADAM metallopeptidase with thrombospondin type 1 motif, 17	0.211

11	1970808	2023641	craniofacial development protein 1	0.220
11	10016612	10144234	carbohydrate (N-acetylgalactosamine 4-0) sulfotransferase 8	0.241
12	3713196	4003351	ATPase, Ca++ transporting, plasma membrane 2	0.261
12	4857558	4878220	peroxisome proliferator-activated receptor gamma	0.204
12	4929821	4939522	probable E3 ubiquitin-protein ligase makorin-2	0.204
12	4943218	4971156	RAF proto-oncogene serine/threonine-protein kinase	0.204
12	5041289	5050563	cellular nucleic acid-binding protein	0.204
12	5783474	5867616	fibulin 2	0.236
12	6023840	6055635	protein Wnt-7a	0.236
12	6125099	6126552	homeobox protein BarH-like 1b	0.236
12	6282467	6367527	PHD finger protein 2	0.236
12	6375304	6426198	family with sequence similarity 120A	0.236
12	6453845	6563101	WNK lysine deficient protein kinase 2	0.236
12	6644909	6670629	sushi domain containing 3	0.236
12	6695375	6799774	FYVE, RhoGEF and PH domain containing 3	0.236
12	6077974	6078065	gga-let-7f	0.236
12	6076930	6077032	gga-let-7d	0.236
12	6078389	6078478	gga-let-7a-1	0.236
12	8976730	9011876	NCK interacting protein with SH3 domain	0.216
12	13983652	14275283	membrane associated guanylate kinase, WW and PDZ domain containing 1	0.251
12	15329789	15396454	FERM domain containing 4B	0.199
12	15468612	15557117	microphthalmia-associated transcription factor	0.199
12	15571303	15598096	hydroxysteroid (17-beta) dehydrogenase 14	0.199
13	3574865	4046215	slit homolog 3 protein precursor	0.222
13	4187413	4738485	teneurin-2 isoform 1	0.243
13	6399216	6470747	Gamma-aminobutyric acid receptor subunit gamma-2	0.215
13	6533385	6575415	Gamma-aminobutyric acid receptor subunit alpha-1	0.215
13	6621733	6646067	Gamma-aminobutyric acid receptor subunit alpha-6	0.215
13	8133602	8180065	serine/threonine kinase 10	0.244
13	8254942	8280226	neuralized homolog 1B (Drosophila)	0.244

13	8380376	8384733	dual specificity protein phosphatase 1	0.244
13	8470024	8474437	60S ribosomal protein L26	0.244
13	8477223	8481039	V-type proton ATPase subunit e 1	0.244
13	8535721	8537935	homeobox protein Nkx-2.5	0.244
13	10737034	10764716	IL2-inducible T-cell kinase	0.243
13	10778915	10785137	hepatitis A virus cellular receptor 1 precursor	0.243
14	6602197	6621651	leucine carboxyl methyltransferase 1	0.194
14	8877922	8897487	cytohesin 3	0.218
14	10103934	10114456	methyltransferase-like protein 22	0.204
14	12090645	12099745	Hemoglobin subunit pi	0.198
15	4855484	4908313	dynein, axonemal, heavy chain 10	0.269
15	4909039	4924155	V-type proton ATPase 116 kDa subunit a isoform 2	0.269
15	4933001	4937788	general transcription factor IIH, polypeptide 3, 34kDa	0.269
15	4941748	4948314	ATP-dependent RNA helicase DDX55	0.269
15	4977536	4979234	small nuclear ribonucleoprotein 35kDa (U11/U12)	0.269
15	4986361	4990790	SET domain containing (lysine methyltransferase) 8	0.269
15	5002524	5026119	protein strawberry notch homolog 1	0.269
15	5038370	5051012	cyclin-dependent kinase 2-associated protein 1	0.269
15	5053460	5077681	M-phase phosphoprotein 9	0.269
15	5080304	5188058	phosphatidylinositol transfer protein, membrane-associated 2	0.269
15	5192449	5195746	ADP-ribosylation factor-like protein 6-interacting protein 4	0.269
15	5208507	5219304	ATP-binding cassette, sub-family B (MDR/TAP), member 9	0.269
15	5243010	5257347	huntingtin-interacting protein 1-related protein	0.269
15	5264075	5270199	Density-regulated protein	0.269
15	5297891	5339287	Sarcoplasmic/endoplasmic reticulum calcium ATPase 2	0.269
15	5368868	5407536	intraflagellar transport protein 81 homolog	0.269
15	5407225	5424559	P2X purinoceptor	0.269
15	5425109	5431053	P2X purinoceptor 4	0.269
15	5446352	5457999	anaphase-promoting complex subunit 5	0.269
15	5460336	5468349	E3 ubiquitin-protein ligase RNF34	0.269

15	5469305	5570414	lysine (K)-specific demethylase 2B	0.269
15	5574193	5584055	calcium release-activated calcium channel protein 1	0.269
15	5585816	5594140	MORN repeat containing 3	0.269
15	5612621	5620330	rho-related GTP-binding protein RhoF	0.269
15	5634231	5677257	histone-lysine N-methyltransferase SETD1B	0.269
15	5682530	5686156	4-hydroxyphenylpyruvate dioxygenase	0.269
15	5686962	5689686	26S proteasome non-ATPase regulatory subunit 9	0.269
15	5691074	5704256	WD repeat domain 66	0.269
15	5749503	5768970	MLX interacting protein	0.269
15	5810440	5862422	CAP-Gly domain-containing linker protein 1	0.269
15	5890702	5903184	arginine/serine-rich coiled-coil 2	0.269
15	5935811	5940908	vacuolar protein sorting-associated protein 29	0.269
15	5941111	5947675	RAD9 homolog B (S. pombe)	0.269
15	5981119	5990657	voltage-gated hydrogen channel 1	0.269
15	5994099	6009307	serine/threonine-protein phosphatase PP1-gamma catalytic subunit	0.269
15	6068672	6089862	cut-like homeobox 2	0.269
15	6102036	6113781	SH2B adaptor protein 3	0.269
15	6176354	6211838	BRCA1-associated protein	0.269
15	6212456	6231931	MAP kinase-activated protein kinase 5	0.269
15	6254850	6258922	Endoplasmic reticulum resident protein 29	0.269
15	6259423	6286521	N-alpha-acetyltransferase 25, NatB auxiliary subunit	0.269
15	6287966	6297369	TRAF-type zinc finger domain containing 1	0.269
15	6301498	6347392	HECT domain containing E3 ubiquitin protein ligase 4	0.269
15	6377025	6380366	60S ribosomal protein L6	0.269
15	6380588	6402006	Tyrosine-protein phosphatase non-receptor type 11	0.269
15	6429258	6443243	rabphilin 3A homolog (mouse)	0.269
15	6530372	6560293	coronin-1C	0.269
15	6610623	6628850	SV2 related protein homolog (rat)	0.269
15	6631170	6642322	ubiquitin specific peptidase 30	0.269
15	5789347	5790438	UDP-GlcNAc:betaGal beta-1,3-N-acetylglucosaminyltransferase 4	0.269

15	7678294	7690938	serine/threonine-protein kinase Chk2	0.234
15	7691060	7694579	HscB iron-sulfur cluster co-chaperone homolog (E. coli)	0.234
15	7713031	7716156	X-box binding protein 1	0.234
15	7808411	7819197	kringle containing transmembrane protein 1	0.234
15	7829620	7845797	sushi domain containing 2	0.234
15	7877611	7915137	DiGeorge syndrome critical region gene 2	0.234
15	7928727	7933299	Ig lambda chain V-1 region	0.234
15	7998509	8008463	SWI/SNF-related matrix-associated actin-dependent regulator of chromatin subfamily B member 1	0.234
15	8010321	8014793	Der1-like domain family, member 3	0.234
15	11212414	11257367	kinase suppressor of ras 2	0.213
15	11287023	11315239	Nitric oxide synthase	0.213
15	11340779	11343535	tescalcin	0.213
15	11434073	11439615	UPF0454 protein C12orf49 homolog	0.213
17	9403856	9424535	G protein-coupled receptor 144	0.204
17	10290287	10360101	Ras-specific guanine nucleotide-releasing factor RalGPS1	0.233
17	10385180	10393957	solute carrier family 2, facilitated glucose transporter member 8	0.233
17	10406697	10443313	GTPase-activating Rap/Ran-GAP domain-like protein 3	0.233
18	2569645	2666638	protoheme IX farnesyltransferase, mitochondrial	0.204
18	7205116	7244436	voltage-dependent calcium channel gamma-4 subunit	0.202
19	955427	1061274	calneuron 1	0.200
20	1510312	1559445	ubiquinol-cytochrome c reductase complex chaperone	0.221
20	1565847	1590302	family with sequence similarity 83, member C	0.221
20	1591146	1598063	eukaryotic translation initiation factor 6	0.221
20	1601528	1644296	matrix metallopeptidase 24 (membrane-inserted)	0.221
20	1643850	1862123	adenosylhomocysteinase	0.221
20	3952514	3969262	myb-related protein B	0.197
20	3973501	3984095	intraflagellar transport protein 52 homolog	0.197
20	4000630	4004091	solute carrier family 32 (GABA vesicular transporter), member 1	0.197
20	4036301	4076622	Rho GTPase-activating protein 18-like	0.197
20	4080901	4091173	Actin-related protein 5	0.197

20	4109316	4154694	protein phosphatase 1 regulatory inhibitor subunit 16B	0.197
20	4159058	4165656	family with sequence similarity 83, member D	0.197
20	5675050	5678607	potassium channel, subfamily K, member 15	0.282
20	5687014	5690326	WNT1 inducible signaling pathway protein 2	0.282
20	5699881	5712513	adenosine deaminase	0.282
20	5740430	5750933	serine incorporator 3 precursor	0.282
20	5748294	5755903	alpha-tocopherol transfer protein-like	0.282
20	5763173	5777781	hepatocyte nuclear factor 4-alpha	0.282
20	5792281	5795875	R3H domain containing-like	0.282
20	5837979	5845069	uncharacterized protein C20orf111 homolog	0.282
20	5847927	5855091	junctophilin 2	0.282
20	5895586	5918161	solute carrier family 13 (sodium-dependent dicarboxylate transporter), member 3	0.282
20	5923593	5925045	TP53 regulating kinase	0.282
20	5928136	5935198	solute carrier family 2 (facilitated glucose transporter), member 10	0.282
20	6012484	6104467	eyes absent homolog 2	0.282
20	6203701	6257441	nuclear receptor coactivator 3	0.282
20	6529946	6608286	phosphatidylinositol-3,4,5-trisphosphate-dependent Rac exchange factor 1	0.282
20	6685428	6743411	ADP-ribosylation factor guanine nucleotide-exchange factor 2 (brefeldin A-inhibited)	0.282
20	6747113	6768662	CSE1 chromosome segregation 1-like (yeast)	0.282
20	6780571	6803177	double-stranded RNA-binding protein Staufen homolog 1	0.282
20	6994464	7004637	transglutaminase 3	0.282
20	7082081	7177518	phosphatase and actin regulator 3	0.282
20	7219136	7226342	family with sequence similarity 217, member B	0.282
20	7702268	8001028	cadherin-4 precursor	0.282
20	7216161	7216973	protein phosphatase 1, regulatory subunit 3D	0.282
20	6814040	6814131	Small nucleolar SNORD12/SNORD106	0.282
20	6811970	6812061	Small nucleolar SNORD12/SNORD106	0.282
20	6813048	6813139	Small nucleolar SNORD12/SNORD106	0.282
20	6814693	6814787	Small nucleolar SNORD12/SNORD106	0.282
21	2676648	2677571	ring finger protein 223	0.205

21	2679190	2699394	protein tyrosine phosphatase, non-receptor type 11-like	0.205
21	2708871	2772320	agrin	0.205
21	2803581	2805434	transcription factor HES-1 isoform 2	0.205
21	2883797	2916830	nucleolar complex protein 2 homolog	0.205
21	2919061	2959863	sterile alpha motif domain-containing protein 11	0.205
21	3033463	3151837	arginine-glutamic acid dipeptide (RE) repeats	0.205
21	4679337	4732155	F-actin-capping protein subunit beta isoforms 1 and 2	0.195
23	861071	915914	human immunodeficiency virus type I enhancer binding protein 3	0.204
25	1033745	1035340	beta-keratin	0.243
25	1132880	1134156	cornulin	0.243
25	1153918	1155640	Protein S100-A11	0.243
25	1156086	1174496	coatomer subunit alpha	0.243
25	1174614	1186654	nicastrin precursor	0.243
26	3240503	3333584	potassium voltage-gated channel subfamily D member 3 precursor	0.206
Z	942435	962360	protein ERGIC-53 precursor	0.273
Z	10001894	10166119	ADAM metallopeptidase with thrombospondin type 1 motif, 12	0.288
Z	10185692	10204950	membrane-associated transporter protein	0.288
Z	10206259	10227283	alpha-methylacyl-CoA racemase	0.288
Z	10173890	10175311	relaxin/insulin-like family peptide receptor 3	0.288
Z	13833652	13891525	fibroblast growth factor 10 precursor	0.293
Z	19339904	19347539	DIM1 dimethyladenosine transferase 1 homolog (S. cerevisiae)	0.298
Z	19348933	19422172	importin 11	0.298
Z	19375862	19375973	U4atac minor spliceosomal RNA	0.298
Z	21981417	22025556	cardiomyopathy associated 5	0.279
Z	26708083	26866140	GLIS family zinc finger 3	0.285
Z	32180631	32551284	basonuclin 2	0.282
Z	38331271	38431898	transducin-like enhancer protein 4	0.328
Z	39039875	39114050	transducin-like enhancer of split 1 (E(sp1) homolog, Drosophila)	0.328
Z	39574349	39615592	RAS and EF-hand domain containing	0.328
Z	39667818	39812156	FERM domain containing 3	0.328

Z	39900490	39924774	kinesin family member 27	0.328
Z	39936510	39954264	Heterogeneous nuclear ribonucleoprotein K	0.328
Z	39954655	39958510	recQ-mediated genome instability protein 1	0.328
Z	38576413	38576570	U1 spliceosomal RNA	0.328
Z	44617583	44658144	CDC42 small effector 2	0.314
Z	46011143	46145593	erythrocyte membrane protein band 4.1 like 4A	0.313
Z	46263610	46283078	neuronal protein 3.1	0.313
Z	46144006	46144137	Small nucleolar RNA SNORA13	0.313
Z	51826653	51834509	tripartite motif-containing protein 14	0.301
Z	51836589	51845476	sialic acid synthase	0.301
Z	51863827	51878606	clathrin light chain A	0.301
Z	51882532	51919708	bifunctional UDP-N-acetylglucosamine 2-epimerase/N-acetylmannosamine kinase	0.301
Z	53357810	53404887	alpha-L-iduronidase precursor	0.289
Z	53427128	53436199	neuronal acetylcholine receptor subunit alpha-6 precursor	0.289
Z	53439539	53450198	neuronal acetylcholine receptor subunit beta-3 precursor	0.289
Z	53495711	53516809	RING finger protein 170	0.289
Z	53516991	53592770	hook homolog 3 (Drosophila)	0.289
Z	54549996	54642132	ATP-binding cassette sub-family A member 1	0.308
Z	54741875	54804427	solute carrier family 44 (choline transporter), member 1	0.308
Z	54832501	54860934	fibronectin type III and SPRY domain containing 1-like	0.308
Z	54866069	54882250	fukutin	0.308
Z	54942939	54943102	U1 spliceosomal RNA	0.308
Z	62344745	62440821	Hyaluronan and proteoglycan link protein 1	0.309
Z	63183171	63185001	ribosomal protein S23	0.303
Z	64830090	64898868	glutamate receptor, ionotropic, N-methyl-D-aspartate 3A	0.291
