# Supplementary material for: Population Genomic Analyses Based on 1 Million SNPs in Commercial Egg Layers
Source: PLoS One. 2014 Apr 16;9(4):e94509. doi: 10.1371/journal.pone.0094509 (PMC3989219; doi:10.1371/journal.pone.0094509)
Supplement: Table S3 — List of genes in lower 1% FST distribution in comparision of brown layers and white layers. (PDF) [file pone.0094509.s003.pdf]

Table S3. List of genes in lower 1% F_{ST} distribution in comparision of brown layers and white layers.

Chr	Start	End	Description	Fst
1	1361642	1822074	exocyst complex component 4	0.059
1	8522175	8850302	semaphorin-3A	0.045
1	9561909	9881531	piccolo presynaptic cytomatrix protein	0.077
1	10471388	10534407	hepatocyte growth factor precursor	0.080
1	14037839	14064141	nicotinamide phosphoribosyltransferase	0.062
1	18537716	18596571	bromodomain-containing protein 1	0.051
1	18646102	18656551	alpha-1,6-mannosyltransferase ALG12 precursor	0.051
1	18657758	18668817	cysteine-rich with EGF-like domain protein 2 precursor	0.051
1	32863934	32927887	ubiquitin carboxyl-terminal hydrolase 15 isoform 1	0.017
1	32942077	33016611	protein MON2 homolog	0.017
1	33019230	33019319	gga-let-7i	0.017
1	45406753	45421533	methionine aminopeptidase 2	0.053
1	45425569	45437573	Ubiquitin carboxyl-terminal hydrolase	0.053
1	45540152	45553445	amidohydrolase domain containing 1	0.053
1	45551218	45563377	histidine ammonia-lyase	0.053
1	45566261	45580903	leukotriene A-4 hydrolase	0.053
1	46574632	46987937	ankyrin repeat and sterile alpha motif domain containing 1B	0.053
1	56390459	56460409	KIAA1549	0.063
1	56510668	56527880	SVOP-like	0.063
1	56529994	56569101	tripartite motif containing 24	0.063
1	56827922	56860120	jumonji C domain containing histone demethylase 1 homolog D (S. cerevisiae)	0.063
1	56901913	56922260	sugar phosphate exchanger 3	0.063
1	56931062	56952880	makorin ring finger protein 1	0.063
1	56983738	57021332	DENN/MADD domain containing 2A	0.063
1	57042452	57053437	aarF domain containing kinase 2	0.063
1	57083560	57138555	Serine/threonine-protein kinase B-raf	0.063
1	60293835	60328513	Transporter	0.081

1	60332193	60357748	Transporter	0.081
1	62933511	63081609	receptor-type tyrosine-protein phosphatase O	0.081
1	66068227	66322847	transcription factor SOX-5	0.081
1	71983303	72042978	Loss of heterozygosity 12 chromosomal region 1 protein homolog	0.025
1	72046586	72113670	dual specificity phosphatase 16	0.025
1	72156810	72166175	G protein-coupled receptor 19	0.025
1	72273762	72277034	cyclin-dependent kinase inhibitor 1B (p27, Kip1)	0.025
1	72476599	72491801	MANSC domain containing 4	0.025
1	72580254	72591026	Parathyroid hormone-related protein Osteostatin	0.025
1	76401717	76426364	non-SMC condensin I complex, subunit D2	0.033
1	76430011	76431726	C-type natriuretic peptide 1 precursor	0.033
1	76434647	76437498	Glyceraldehyde-3-phosphate dehydrogenase	0.033
1	76463548	76468438	NOP2 nucleolar protein	0.033
1	76468778	76489284	chromodomain helicase DNA binding protein 4	0.033
1	76507821	76515304	lysophosphatidic acid receptor 5	0.033
1	76520460	76539139	acrosin binding protein	0.033
1	76472752	76472892	Small Cajal body specific RNA 11	0.033
1	79971022	79992959	chromodomain helicase DNA binding protein 1-like	0.040
1	80151496	80204080	Rho GTPase activating protein 31	0.033
1	80216650	80227498	uroplakin 1B	0.033
1	80278967	80367961	immunoglobulin superfamily, member 11	0.033
1	81680485	81734254	growth associated protein 43	0.033
1	86815012	86834017	leukocyte surface antigen CD47 precursor	0.056
1	86848940	86867576	intraflagellar transport 57 homolog (Chlamydomonas)	0.056
1	88543354	88765491	ephrin type-A receptor 3 precursor	0.040
1	90781478	90861444	DDB1 and CUL4 associated factor 6	0.071
1	90911605	90946738	protein kinase substrate MK2S4	0.071
1	90955206	90960077	Protein CREG1	0.071
1	91027333	91088674	POU domain, class 2, transcription factor 1	0.071
1	91174651	91184283	glycoprotein A33 (transmembrane)	0.060

1	91759587	91767822	acid phosphatase 6, lysophosphatidic	0.060
1	91781764	91800664	gap junction alpha-5 protein	0.060
1	98638187	98668892	coxsackie virus and adenovirus receptor	0.035
1	98700854	98720026	protein EURL	0.035
1	98348216	98348323	gga-let-7c	0.035
1	101488423	101488527	U6 spliceosomal RNA	0.073
1	117479797	117519398	phosphate cytidylyltransferase 1, choline, beta	0.044
1	117529894	117579804		0.044
1	119243266	119250553	eukaryotic translation initiation factor 1A, Y chromosome	0.044
1	119314259	119348635	MAP7 domain containing 2	0.044
1	119398188	119620052	SH3 domain-containing kinase-binding protein 1	0.044
1	119632060	119703945	mitogen-activated protein kinase kinase kinase 15	0.078
1	119705079	119717030	pyruvate dehydrogenase E1 component subunit alpha, somatic form, mitochondrial precursor	0.078
1	119875127	119904791	protein phosphatase, EF-hand calcium binding domain 1	0.078
1	119914133	119926065	retinoschisin 1 precursor	0.078
1	120080416	120151665	sex comb on midleg-like 2 (Drosophila)	0.078
1	127508430	127539291	protein kinase, X-linked	0.078
1	127679910	127698770	matrix-remodelling associated 5	0.078
1	130314510	130614689	gamma-aminobutyric acid (GABA) A receptor, gamma 3	0.076
1	143798561	143884756	Ubiquitin-associated domain-containing protein 2	0.077
1	161506641	161608657	tudor domain-containing protein 3	0.044
1	169373629	169393173	serpin peptidase inhibitor, clade E (nexin, plasminogen activator inhibitor type 1), member 3	0.072
1	169452454	169518079	WD repeat and FYVE domain-containing protein 2	0.072
1	169616748	169647630	ATPase, Cu++ transporting, beta polypeptide	0.057
1	169657527	169662281	asparagine-linked glycosylation protein 11 homolog	0.057
1	169666484	169685951	NIMA-related kinase 5	0.057
1	169701518	169710954	cytoskeleton-associated protein 2	0.057
1	174734826	174993334	microtubule associated tumor suppressor candidate 2	0.069
1	182715565	183138508	contactin-5 precursor	0.061
1	193951674	193966948	diacylglycerol O-acyltransferase 2	0.070

2	27518562	27854244	diacylglycerol kinase, beta 90kDa	0.078
2	31779228	31825127	oxysterol binding protein-like 3	0.072
2	73536943	73537003	Small nucleolar RNA R11/Z151	0.064
2	77784558	78033401	catenin (cadherin-associated protein), delta 2	0.075
2	93602166	93685718	rotatin	0.046
2	93737182	93981838	docking protein 6	0.046
2	94235832	94263036	thioredoxin-related transmembrane protein 3	0.046
2	95477061	95554752	cadherin-7 precursor	0.078
2	97607396	97902750	piezo-type mechanosensitive ion channel component 2	0.074
2	110780501	110807257	trimethylguanosine synthase	0.049
2	110831287	110878413	tyrosine-protein kinase Lyn	0.049
2	110935099	110937180	Zinc finger protein PLAG1	0.049
2	111004143	111017846	epidermal retinol dehydrogenase 2	0.049
2	111052464	111060360	short chain dehydrogenase/reductase family 16C, member 5	0.049
2	110920976	110922025	Serine/threonine-protein kinase mos	0.049
2	110902005	110902071	Small nucleolar RNA U54	0.049
2	111761499	111804172	neutral sphingomyelinase (N-SMase) activation associated factor	0.079
2	112612964	112699976	Chromodomain-helicase-DNA-binding protein 7	0.050
2	113502155	113517713	tocopherol (alpha) transfer protein	0.050
2	113543309	113551391	YTH domain family protein 3	0.050
2	119025058	119150906	Zinc finger homeobox protein 4	0.047
2	119193465	119216508	peroxin 2	0.047
2	121655553	121655716	U1 spliceosomal RNA	0.049
2	125844098	125903394	DNA repair and recombination protein RAD54B	0.058
2	125907865	125917414	ring finger protein 151	0.058
2	125925066	125952171	KIAA1429	0.058
2	125956620	125988739	epithelial splicing regulatory protein 1	0.058
2	126052840	126076741	dpy-19-like 4 (C. elegans)	0.058
2	126082914	126125825	cHz-cadherin precursor	0.058
2	130565762	130772206	oxidation resistance 1	0.043

2	130946459	131114196	angiopoietin-1 precursor	0.043
2	131277175	131326213	R-spondin 2	0.043
2	131405148	131425618	eukaryotic translation initiation factor 3 subunit E	0.043
2	131497565	131532447	tetratricopeptide repeat protein 35	0.043
2	131690687	131704507	thyrotropin-releasing hormone receptor	0.043
2	131729098	131754322	NudC domain containing 1	0.043
2	131770048	131789265	receptor-binding cancer antigen expressed on SiSo cells	0.043
2	131790739	131821463	syntabulin (syntaxin-interacting)	0.043
2	132812344	133381395	CUB and Sushi multiple domains 3	0.043
2	140145617	140283188	ArfGAP with SH3 domain, ankyrin repeat and PH domain 1	0.009
2	140759405	140759511	U6 spliceosomal RNA	0.009
2	142700486	142776708	KH domain containing, RNA binding, signal transduction associated 3	0.035
2	145622308	145655804	solute carrier family 45, member 4	0.014
3	6450555	7094776	neurexin-1-alpha isoform 1 precursor	0.018
3	7268194	7268297	U6 spliceosomal RNA	0.018
3	10819837	10824742	Ewing tumor-associated antigen 1	0.056
3	11105742	11120517	nuclear nucleic acid-binding protein C1D	0.056
3	11131168	11137396	WD repeat-containing protein 92	0.056
3	11137452	11140992	RNA-binding protein PNO1	0.056
3	11142465	11175965	calcineurin subunit B type 1	0.056
3	11215025	11230471	pleckstrin	0.056
3	12135230	12217452	serine palmitoyltransferase, long chain base subunit 3	0.076
3	12844082	12878172	Delta-like protein	0.068
3	12887313	12960238	UPF0492 protein C20orf94 homolog	0.068
3	12961577	12970854	McKusick-Kaufman/Bardet-Biedl syndromes putative chaperonin	0.068
3	12995964	13027937	Synaptosomal-associated protein 25	0.068
3	13125328	13287642	serine/threonine-protein kinase PAK 7	0.068
3	13593275	13906402	phospholipase C, beta 1 (phosphoinositide-specific)	0.079
3	16515941	16518570	left-right determination factor 2 precursor	0.054
3	20175604	20250749	potassium channel, subfamily K, member 2	0.053

3	21605815	21621949	TNF receptor-associated factor 5	0.069
3	21628366	21650478	REST corepressor 3	0.069
3	22667612	22700224	protein kinase domain containing, cytoplasmic	0.050
3	24831731	24859714	prolyl endopeptidase-like	0.070
3	26292215	26454063	tetratricopeptide repeat protein 7A	0.017
3	31632109	31793597	cysteine-rich motor neuron 1 protein precursor	0.065
3	33406631	33650468	SET and MYND domain containing 3	0.029
3	33669293	33956337	kinesin family member 26B	0.029
3	33972419	33996834	EF-hand calcium binding domain 2	0.029
3	34015772	34030943	heterogeneous nuclear ribonucleoprotein U (scaffold attachment factor A)	0.029
3	34039072	34043740	COX20 cytochrome C oxidase assembly factor	0.029
3	34059728	34074390	Desumoylating isopeptidase 2	0.029
3	34116278	34136305	Adenylosuccinate synthetase isozyme 2	0.029
3	34322339	34455636	v-akt murine thymoma viral oncogene homolog 3	0.029
3	34464087	34563969	serologically defined colon cancer antigen 8	0.029
3	34813540	34977620	inactive phospholipase D5	0.029
3	36498791	36661068	ryanodine receptor 2 (cardiac)	0.050
3	37135755	37159463	integral membrane protein GPR137B	0.060
3	45769623	45811385	epilepsy, progressive myoclonus type 2A, Lafora disease (laforin)	0.060
3	45868769	45921436	SNF2 histone linker PHD RING helicase, E3 ubiquitin protein ligase	0.060
3	45937157	46118743	Glutamate receptor metabotropic 1 isoform f transcript variant 1	0.060
3	48447032	48720659	spectrin repeat containing, nuclear envelope 1	0.055
3	48776284	48795822	VIP peptides isoform 2 preproprotein	0.055
3	48910153	48981998	Regulator of G-protein signaling 17	0.055
3	49319843	49341770	opioid receptor, mu 1	0.055
3	50465764	50754522	AT rich interactive domain 1B (SWI1-like)	0.015
3	59223034	59291522	nuclear receptor coactivator 7	0.069
3	77166449	77331130	NADP-dependent malic enzyme	0.047
3	77404161	77449989	ubiquitin protein ligase E3D	0.047
3	77659755	77717628	inhibitor of Bruton agammaglobulinemia tyrosine kinase	0.047

3	77839636	77873063	uncharacterized protein LOC421845	0.047
3	78457902	78570667	2-oxoisovalerate dehydrogenase subunit beta, mitochondrial precursor	0.047
3	80913076	80916878	Elongation factor 1-alpha 1	0.065
3	80928945	80936582	protein MTO1 homolog, mitochondrial	0.065
3	80937537	80943626	Mab-21 domain containing 1	0.065
3	80962161	81225706	potassium voltage-gated channel, KQT-like subfamily, member 5	0.065
3	82085759	82150209	family with sequence similarity 135, member A	0.067
3	88949756	89752149	CUB and Sushi multiple domains 1	0.067
3	92589243	92805041	myelin transcription factor 1-like	0.057
3	95513204	95603900	Membrane-bound O-acyltransferase domain-containing protein 2	0.044
3	95762549	95776222	cleavage and polyadenylation specific factor 3, 73kDa	0.044
3	95785003	95816690	disintegrin and metalloproteinase domain-containing protein 17 precursor	0.044
3	95838634	95862299	14-3-3 protein theta	0.044
3	101880367	101916760	apolipoprotein B precursor	0.044
3	101958856	101964309	tudor domain containing 15	0.044
4	247357	254601	V-set and immunoglobulin domain containing 4	0.069
4	261649	275014	Heat shock factor protein 3	0.069
4	3175772	3206798	kelch-like protein 13	0.060
4	10622204	10691620	Gamma-aminobutyric acid receptor subunit beta-4	0.077
4	20013683	20014840	Neuropeptide Y receptor type 2	0.058
4	32292871	32664499	LPS-responsive vesicle trafficking, beach and anchor containing	0.079
4	38695965	38707538	ufm1-specific protease 2	0.011
4	38707911	38715236	LRP2 binding protein	0.011
4	38717204	38769907	sorting nexin 25	0.011
4	38786566	38804359	KIAA1430	0.011
4	38807037	38809857	ADP/ATP translocase 1	0.011
4	38883037	38919985	long-chain-fatty-acid--CoA ligase 1	0.011
4	38921548	38931422	centromere protein U	0.011
4	38947850	38962863	caspase-3	0.011
4	38987654	39011877	Interferon regulatory factor 2	0.011

4	39065322	39190579	storkhead box 2	0.011
4	39200828	39225725	trafficking protein particle complex subunit 11	0.011
4	39278893	39281865	CDKN2A interacting protein	0.011
4	39331594	39416540	WW and C2 domain containing 2	0.011
4	39276202	39276488	RNase MRP	0.011
4	44658935	44721258	anthrax toxin receptor 2	0.064
4	51964136	51964316	TUC338	0.062
4	56333065	56644463	Ankyrin 2; Uncharacterized protein	0.000
4	57960584	58196338	bone morphogenetic protein receptor type-1B precursor	0.068
4	76956828	76994096	biorientation of chromosomes in cell division 1-like 1	0.060
4	82783104	82842064	Wolf-Hirschhorn syndrome candidate 1	0.029
5	969359	999049	regulator of microtubule dynamics 3	0.034
5	1003403	1011083	GTP cyclohydrolase 1 feedback regulatory protein	0.034
5	1011384	1027952	DnaJ (Hsp40) homolog, subfamily C, member 17	0.034
5	1028182	1029979	Olfactory receptor-like protein COR9	0.034
5	1107502	1274845	leucine zipper protein 2	0.034
5	1041317	1042285	Olfactory receptor-like protein COR9	0.034
5	1046130	1047068	olfactory receptor-like protein COR4	0.034
5	13709148	14005932	BR serine/threonine-protein kinase 2	0.081
5	14631769	14696703	tetraspanin 4	0.075
5	18248884	18303314	CD44 antigen precursor	0.075
5	20702880	20716727	Apoptosis inhibitor 5	0.058
5	20718591	20772087	tetratricopeptide repeat domain 17	0.058
5	20863830	20943207	hydroxysteroid (17-beta) dehydrogenase 12	0.058
5	20943446	20959283	alkB, alkylation repair homolog 3 (E. coli)	0.058
5	21022967	21094349	exostosin glycosyltransferase 2	0.058
5	20572511	20572677	TUC338	0.058
5	21901389	21963072	alpha-(1,6)-fucosyltransferase	0.018
5	25492780	25585952	protein numb homolog	0.055
5	31481161	31528089	aquarius homolog (mouse)	0.029

5	31546121	31552902	Actin, alpha cardiac muscle 1	0.029
5	31568439	31572498	gap junction delta-2 protein	0.029
5	31693846	31748417	syntaxin-binding protein 6	0.029
5	32087430	32252521	RNA-binding protein Nova-1 isoform 1	0.029
5	32002939	32003093	U1 spliceosomal RNA	0.029
5	35909286	35910879	NK2 homeobox 1	0.078
5	35952997	35955492	NK2 homeobox 8	0.078
5	35997255	36015533	paired box protein Pax-9	0.078
5	36015953	36247167	solute carrier family 25 (mitochondrial oxoadipate carrier), member 21	0.078
5	39826229	39837879	Iodothyronine deiodinase	0.040
5	40042540	40090213	thyrotropin receptor isoform 1 precursor	0.040
5	40099534	40119234	transcription initiation factor IIA subunit 1	0.040
5	40144828	40210981	stonin 2	0.040
5	48312771	48378821	ena/VASP-like protein	0.014
5	51711521	51736637	AHNAK nucleoprotein 2	0.073
5	52834900	52872470	C-1-tetrahydrofolate synthase, cytoplasmic	0.072
6	11114347	11184726	sphingosine-1-phosphate lyase 1	0.024
6	11185502	11189454	Pterin-4-alpha-carbinolamine dehydratase	0.024
6	12717748	12721551	40S ribosomal protein S24	0.066
6	12723104	12753909	DNA-directed RNA polymerase III subunit RPC1	0.066
6	15645287	15696643	ubiquitin specific peptidase 54	0.034
6	15705273	15750035	serine/threonine-protein phosphatase 2B catalytic subunit beta isoform	0.034
6	15752205	15762949	Annexin	0.034
6	15922043	15977706	SH3 domain containing ring finger 1	0.034
6	16111040	16115551	zona pellucida sperm-binding protein 4	0.034
6	16971567	16974297	NADH dehydrogenase	0.041
6	16975583	16995413	SEC31 homolog B (S. cerevisiae)	0.041
6	17984757	18098872	WDFY family member 4	0.005
6	23670969	23706526	Collagen alpha-1(XVII) chain 120 kDa linear IgA disease antigen homolog	0.072
6	23730903	23734477	Swi5-dependent recombination DNA repair protein 1 homolog	0.072

7	571200	611944	collagen alpha-1(III) chain precursor	0.044
7	2220524	2272149	NCK-associated protein 1	0.059
7	2280810	2296210	secreted frizzled-related protein 3 precursor	0.059
7	9174695	9279054	dynein, axonemal, heavy chain 7	0.073
7	9281021	9299762	serine/threonine-protein kinase 17B	0.073
7	9822436	9943072	phospholipase C-like 1	0.045
7	11084221	11117761	cyclin-dependent kinase 15	0.073
7	21176600	21260777	RNA-binding motif, single-stranded-interacting protein 1	0.053
7	24731579	24765366	ras-related protein M-Ras	0.028
7	24931014	24973586	erythrocyte membrane protein band 4.1 like 5	0.028
7	25039352	25049178	Zinc finger protein GLI2	0.028
7	25068118	25068894	Inhibin beta B chain	0.028
7	28692505	28874290	dipeptidyl-peptidase 10 (non-functional)	0.061
8	6430857	6533346	RAS protein activator like 2	0.074
8	11522078	11839939	dihydropyrimidine dehydrogenase	0.051
8	11896946	11945494	polypyrimidine tract binding protein 2	0.051
8	12939656	12962759	metal-response element-binding transcription factor 2	0.067
8	12994887	13001198	60S ribosomal protein L5	0.067
8	12997780	12997875	Small nucleolar RNA SNORD21	0.067
8	15416743	15437448	vitellogenin-2 precursor	0.078
8	17790799	17811115	far upstream element-binding protein 1	0.080
8	17817585	17833703	nexilin (F actin binding protein)	0.080
8	22464843	22521965	ELAV-like protein 4	0.078
8	23159775	23170004	transcription factor BTF3 homolog 4	0.053
8	23276661	23291886	origin recognition complex subunit 1	0.053
8	23344643	23353210	glutathione peroxidase 7	0.053
8	25948005	26179911	nuclear factor 1 A-type	0.042
9	6123833	6124579	splA/ryanodine receptor domain and SOCS box containing 4	0.033
9	9788144	9809727	Sodium/potassium-transporting ATPase subunit beta-3	0.067
9	9818914	9863003	transcription factor Dp-2	0.067

9	9877217	9898347	Putative glycerol kinase 5	0.067
9	11061796	11069117	phospholipid scramblase family, member 5	0.077
9	11938121	11940148	phosphatidylinositol glycan anchor biosynthesis, class Z	0.018
9	11940276	11956868	melanotransferrin precursor	0.018
9	11959073	12089444	discs, large homolog 1 (Drosophila)	0.018
9	11878868	11880012	Type-1 angiotensin II receptor	0.018
10	5730113	5734914	TM2 domain containing 3	0.067
10	5735057	5743901	adenosine deaminase-like protein	0.067
10	5747497	5754751	La ribonucleoprotein domain family, member 6	0.067
10	5755975	5794821	leucine rich repeat containing 49	0.067
10	5850094	6086027	thrombospondin, type I, domain containing 4	0.067
10	11600710	11632184	interleukin 16	0.032
10	11728514	11734390	LDLR chaperone MESD	0.032
10	11740664	11788096	KIAA1199	0.032
10	11847474	11877912	Abhydrolase domain-containing protein FAM108C1	0.032
10	11900896	11979084	aryl-hydrocarbon receptor nuclear translocator 2	0.032
10	13154344	13445021	ATP/GTP binding protein-like 1	0.039
10	15328163	15333861	COUP transcription factor 2	0.050
10	15374883	15375051	TUC338	0.050
10	16601477	16763501	ADAM metallopeptidase with thrombospondin type 1 motif, 17	0.033
10	16795493	16809456	ceramide synthase 3	0.033
10	16824590	16834400	lines homolog (Drosophila)	0.033
11	2470647	2483811	dynein, axonemal, assembly factor 1	0.070
11	2484347	2490880	hydroxysteroid dehydrogenase-like protein 1	0.070
11	10376830	10403785	KIAA0355	0.080
11	13255162	13323250	ADAM metallopeptidase with thrombospondin type 1 motif, 18	0.011
11	13424046	13427036	nudix (nucleoside diphosphate linked moiety X)-type motif 7	0.011
11	13623072	13643343	WW domain-containing oxidoreductase	0.011
12	3333636	3452411	centromere protein P	0.055
12	3504495	3594440	isoleucyl-tRNA synthetase	0.055

12	3505491	3505625	Small nucleolar RNA SNORA84	0.055
12	4379532	4465392	Ubiquitin-like modifier-activating enzyme ATG7	0.017
12	8701129	8705214	phosphodiesterase 12	0.057
12	8708924	8713617	ADP-ribosylation factor 5	0.057
12	8722034	8741878	protein FAM116A	0.057
12	8851102	8917084	filamin-B	0.057
12	12141669	12521936	fragile histidine triad	0.056
13	365453	377641	histidyl-tRNA synthetase, cytoplasmic	0.074
13	384539	392849	protein Red	0.074
13	392987	394389	NADH dehydrogenase	0.074
13	380747	380840	Vault RNA	0.074
13	3332166	3492561	dedicator of cytokinesis 2	0.008
13	3402277	3426685	family with sequence similarity 196, member B	0.008
14	8324363	8383242	SMG1 phosphatidylinositol 3-kinase-related kinase	0.055
14	9098581	9109757	trans-golgi network vesicle protein 23 homolog A (S. cerevisiae)	0.078
14	9109210	9113944	nucleotide binding protein 1 (MinD homolog, E. coli)	0.078
14	8935374	8936082	suppressor of cytokine signaling 1	0.078
14	10846837	11212402	RNA binding protein, fox-1 homolog (C. elegans) 1	0.060
14	14919945	14927428	ERI1 exoribonuclease family member 2	0.009
14	14929360	14941980	exonuclease NEF-sp	0.009
14	14972896	14976954	LYR motif containing 1	0.009
14	14983212	15045881	dynein, axonemal, heavy chain 3	0.009
15	2912110	3202073	syntaxin-2	0.051
15	3051055	3169587	G protein-coupled receptor 133	0.051
15	3179902	3184192	GTP-binding nuclear protein Ran	0.051
15	3219108	3322455	RIMS-binding protein 2	0.051
15	3029980	3030156	TUC338	0.051
16	225233	229543	kinesin family member C1	0.073
16	291965	332200	intestinal zipper protein	0.073
16	335307	346680	MHC B-G antigen isoform 2 precursor	0.073

17	245842	271719	ankyrin repeat and MYND domain containing 1	0.081
17	377513	388937	suppressor APC domain containing 2	0.081
17	402427	410036	ectonucleoside triphosphate diphosphohydrolase 2 precursor	0.081
17	415522	418106	chloride intracellular channel 3	0.081
17	420847	451524	ATP-binding cassette, sub-family A (ABC1), member 2	0.081
17	1826168	1863962	general transcription factor IIH, polypeptide 4 precursor	0.028
17	1871842	1873939	tubulin beta-3 chain	0.028
17	1897293	1908429	cofactor of BRCA1	0.028
17	1933420	1939035	Glutamine synthetase	0.028
17	1955243	1981882	arrestin domain-containing protein 1	0.028
17	2025623	2080997	histone-lysine N-methyltransferase, H3 lysine-9 specific 5	0.028
17	2100704	2358309	Voltage-dependent N-type calcium channel subunit alpha-1B	0.028
17	7329258	7353026	retinoid X receptor, alpha	0.053
17	7462510	7561096	collagen alpha-1(V) chain precursor	0.053
17	7609613	7627804	Noelin	0.053
17	7710495	7713122	protein phosphatase 1, regulatory subunit 26	0.053
17	7737254	7744146	1-acylglycerol-3-phosphate O-acyltransferase 2	0.053
17	7723324	7723456	Small nucleolar RNA SNORA17	0.053
17	7722798	7722930	Small nucleolar RNA SNORA17	0.053
18	6254682	6261401	tripartite motif containing 25	0.052
18	6262355	6267812	coilin	0.052
18	6267875	6278615	serine carboxypeptidase 1 precursor	0.052
18	6282874	6369966	RAB11 family interacting protein 4 (class II)	0.052
18	6162700	6163371	noggin precursor	0.052
18	7895218	7917904	axin-2	0.065
18	7997936	8029708	regulator of G-protein signaling 9	0.065
18	8091453	8108190	arylsulfatase G	0.065
18	10096967	10115046	CDP-diacylglycerol--glycerol-3-phosphate 3-phosphatidyltransferase, mitochondrial	0.041
18	10142947	10144362	Thymidine kinase, cytosolic	0.041
18	10145914	10147671	synaptogyrin 2	0.041

18	10154424	10156574	ADP-ribosylation factor-like 16	0.041
18	10181308	10184603	gastric inhibitory polypeptide receptor precursor	0.041
18	10183770	10202043	protein disulfide-isomerase precursor	0.041
18	10205975	10214784	rho GDP-dissociation inhibitor 1	0.041
18	10214965	10219808	Aly/REF export factor	0.041
18	10233635	10238009	NAD-dependent deacetylase sirtuin-7	0.041
18	10240959	10242628	Transcription factor MafG	0.041
18	10253363	10256902	nucleoside diphosphate kinase A	0.041
18	10259334	10316575	sperm associated antigen 9	0.041
18	10393676	10406868	luc7-like protein 3	0.041
18	10407626	10414276	ankyrin repeat domain-containing protein 40	0.041
18	10415256	10447157	ATP-binding cassette, sub-family C (CFTR/MRP), member 3	0.041
18	10578502	10590280	spermatogenesis associated 20	0.041
18	10591546	10596249	epsin 3	0.041
18	10670853	10683456	xylosyltransferase 2	0.041
18	10683850	10688333	CD300a molecule precursor	0.041
18	10690316	10691046	uncharacterized protein LOC769812 precursor	0.041
18	10703909	10709349	RAB37, member RAS oncogene family	0.041
18	10711117	10718410	Na(+)/H(+) exchange regulatory cofactor NHE-RF1	0.041
18	10769812	10777868	glutamate receptor, ionotropic, N-methyl D-aspartate 2C	0.041
18	10812362	10827732	Usher syndrome 1G (autosomal recessive)	0.041
18	10823003	10827945	otopetrin 2	0.041
18	10832874	10838829	otopetrin 3	0.041
18	10867250	10883572	cerebellar degeneration-related protein 2-like	0.041
18	10900894	10907983	BTB/POZ domain-containing protein KCTD2	0.041
18	10116718	10117433	suppressor of cytokine signaling 3	0.041
18	10354079	10355879	protein Tob1	0.041
19	9002904	9082184	Neurofibromin	0.060
19	9024293	9027844	protein EVI2A precursor	0.060
19	9094394	9101824	WD repeat and SOCS box-containing protein 1	0.060

19	9192393	9211569	nitric oxide synthase, inducible	0.060
19	9301908	9313939	BTB/POZ domain-containing adapter for CUL3-mediated RhoA degradation protein 2	0.060
19	9316575	9360577	small G protein signaling modulator 2	0.060
19	9436637	9465791	Lissencephaly-1 homolog	0.060
19	9525771	9567750	Rap1 GTPase-activating protein 2	0.060
19	9059359	9059512	TUC338	0.060
20	9169385	9212722	collagen alpha-1(XX) chain precursor	0.060
20	9218845	9237447	Neuronal acetylcholine receptor subunit alpha-4	0.060
20	9348414	9360272	Elongation factor 1-alpha 1	0.060
20	9373970	9382871	src-related kinase lacking C-terminal regulatory tyrosine and N-terminal myristylation sites	0.060
20	10772139	10813719	catenin, beta like 1	0.079
20	13731017	13739373	dolichyl-phosphate mannosyltransferase polypeptide 1, catalytic subunit	0.039
20	13811824	13824523	partitioning defective 6 homolog beta	0.039
20	13824100	13879197	family with sequence similarity 65, member C	0.039
20	13883933	13922802	Tyrosine-protein phosphatase non-receptor type 1	0.039
20	14084227	14098181	Ubiquitin-conjugating enzyme E2 variant 1	0.039
20	14054696	14055682	CCAAT/enhancer-binding protein beta	0.039
20	14046407	14046516	TUC338	0.039
21	1432928	1434563	transcription factor HES-5	0.040
21	1435527	1459981	pantothenate kinase 4	0.040
21	1456791	1466358	Probable glutamate receptor	0.040
21	1552285	1555875	peroxisome biogenesis factor 10	0.040
21	1558528	1565754	Protein RER1	0.040
21	1679120	1769271	ski oncogene	0.040
21	1849380	1856932	gamma-aminobutyric acid (GABA) A receptor, delta	0.040
21	3252441	3261217	G protein-coupled receptor 157	0.048
21	3470173	3492933	phosphatidylinositol-4,5-bisphosphate 3-kinase catalytic subunit delta isoform	0.048
21	3493075	3527740	calsyntenin-1 precursor	0.048
21	3573056	3581801	Protein LZIC	0.048
21	3581913	3588648	nicotinamide nucleotide adenylyltransferase 1	0.048

21	3590985	3592942	retinol binding protein 7, cellular	0.048
21	3595756	3630094	ubiquitination factor E4B	0.048
21	3639974	3713856	kinesin family member 1B	0.048
21	3719439	3729173	6-phosphogluconate dehydrogenase, decarboxylating	0.048
21	3730260	3733060	Centromere protein S	0.048
21	3705900	3706069	TUC338	0.048
22	243826	248360	gastrokine 1	0.000
22	276543	282395	Bone morphogenetic protein-10; Uncharacterized protein	0.000
22	291968	308523	rho GTPase-activating protein 25	0.000
22	817220	825282	BTB/POZ domain-containing protein KCTD9	0.078
22	827828	830831	Progonadoliberin-1 Gonadoliberin-1 GnRH-associated peptide 1	0.078
22	1597651	1602693	TELO2 interacting protein 2	0.072
22	1989333	2026713	potassium channel, subfamily U, member 1	0.072
22	2203023	2212432	G protein-coupled receptor 124	0.072
22	2219556	2228649	RAB11 family interacting protein 1 (class I)	0.072
22	2230210	2232651	prolactin-releasing peptide receptor-like protein	0.072
23	3502047	3514489	metal regulatory transcription factor 1	0.055
23	4176483	4190009	claspin	0.056
23	4222202	4234872	Proteasome subunit beta type	0.056
23	4256435	4263091	neurochondrin	0.056
23	4264511	4288627	KIAA0319-like	0.056
23	4295102	4296014	interferon alpha-inducible protein 27-like protein 2	0.056
23	4343978	4352202	Gizzard PTB-associated splicing factor; Uncharacterized protein	0.056
23	4361390	4361969	ZMYM6 neighbor	0.056
23	4379948	4393033	discs, large (Drosophila) homolog-associated protein 3	0.056
23	4398099	4400981	connexin 37	0.056
23	4570631	4804042	CUB and Sushi multiple domains 2	0.056
23	4824370	4834663	collagen, type IX, alpha 2	0.056
23	4835192	4847417	small ArfGAP2	0.056
23	4901524	4907272	potassium voltage-gated channel, KQT-like subfamily, member 4	0.056

23	4910836	4916448	tubulointerstitial nephritis antigen-like 1	0.056
23	4918792	4920966	penta-EF-hand domain containing 1	0.056
23	4921631	4939423	collagen, type XVI, alpha 1	0.056
23	4943828	4960852	brain-specific angiogenesis inhibitor 2	0.056
23	5033894	5048705	KH domain-containing, RNA-binding, signal transduction-associated protein 1	0.056
23	5072000	5074434	MARCKS-related protein	0.056
23	5075963	5089294	Histone deacetylase 1	0.056
23	5103396	5107519	eukaryotic translation initiation factor 3 subunit I	0.056
23	5108365	5113741	doublecortin domain containing 2B	0.056
23	5118998	5126267	taxilin alpha	0.056
23	5126931	5142990	importin subunit alpha-7	0.056
23	5145916	5152332	BSD domain-containing protein 1	0.056
23	5179676	5186712	histone-binding protein RBBP4	0.056
23	5208687	5215113	KIAA1522	0.056
23	5216609	5221415	tyrosyl-tRNA synthetase, cytoplasmic	0.056
23	5229742	5235821	fibronectin type III domain containing 5	0.056
23	5283571	5286976	arginine decarboxylase	0.056
23	4404012	4404779	gap junction protein, beta 3, 31kDa	0.056
24	62299	94625	salt-inducible kinase 2	0.067
24	121487	131479	fasciculation and elongation protein zeta 1 (zygin I)	0.067
24	134977	155779	homeobox protein PKNOX2	0.067
24	32960	33547	H2A histone family, member X	0.067
24	5312963	5393749	Down syndrome cell adhesion molecule like 1	0.048
24	5401365	5409343	FXYD domain-containing ion transport regulator 6 precursor	0.048
24	5424232	5428001	interleukin-10 receptor subunit alpha precursor	0.048
24	5448267	5451407	sodium channel, voltage-gated, type II, beta subunit	0.048
24	5459063	5464891	myelin protein zero-like protein 3	0.048
24	5465068	5470649	myelin protein zero-like 2 precursor	0.048
24	5498317	5504696	Coatomer subunit delta	0.048
24	5574124	5587498	probable ATP-dependent RNA helicase DDX6	0.048

24	5590151	5595172	C-X-C chemokine receptor type 5	0.048
24	5601465	5612457	B-cell CLL/lymphoma 9-like	0.048
24	5657151	5658883	ribosomal protein S25	0.048
24	5658964	5660793	trafficking protein particle complex subunit 4	0.048
24	5661176	5668655	solute carrier family 37 (glucose-6-phosphate transporter), member 4	0.048
24	5669910	5683409	hypoxia up-regulated protein 1 precursor	0.048
24	5684174	5705399	pleckstrin homology-like domain, family B, member 1	0.048
24	5732695	5757116	D(2) dopamine receptor	0.048
24	5786865	5800976	tetratricopeptide repeat domain 12	0.048
24	5803421	5884198	neural cell adhesion molecule 1 precursor	0.048
24	6163150	6165998	interleukin-18	0.048
24	6167375	6171382	succinate dehydrogenase	0.048
24	6176797	6179439	PIH1 domain containing 2	0.048
24	6230556	6234006	heat shock 27kDa protein 2	0.048
24	6235234	6238779	Alpha-crystallin B chain	0.048
24	6249882	6274575	ALG9, alpha-1,2-mannosyltransferase	0.048
25	1132880	1134156	cornulin	0.054
25	1696346	1699747	translocon-associated protein subunit beta precursor	0.071
25	1700503	1705069	cathepsin S precursor	0.071
25	1705617	1707797	cathepsin K precursor	0.071
25	1709351	1730021	aryl hydrocarbon receptor nuclear translocator	0.071
25	1751126	1755032	family with sequence similarity 63, member A	0.071
25	1761749	1763263	CDC42 small effector protein 1	0.071
25	1777988	1783588	sema domain, transmembrane domain (TM), and cytoplasmic domain, (semaphorin) 6C	0.071
25	1788992	1961542	tropomodulin 4 (muscle)	0.071
25	1792982	1795236	vacuolar protein sorting 72 homolog (S. cerevisiae)	0.071
26	61868	80894	peptidyl-prolyl cis-trans isomerase FKBP5	0.035
26	87655	88370	colipase precursor	0.035
26	89352	93029	Tetraspan membrane protein of hair cell stereocilia homolog	0.035
26	95396	113328	SRSF protein kinase 1	0.035

26	184444	202634	bromodomain and PHD finger containing, 3	0.035
26	224386	227839	pim-1 oncogene	0.035
26	246613	275469	Voltage-dependent L-type calcium channel subunit alpha-1S	0.035
26	276159	308776	kinesin family member 21B	0.035
27	116781	119543	Dolichyl-diphosphooligosaccharide--protein glycosyltransferase subunit DAD1	0.026
27	1056370	1079949	gametocyte specific factor 1	0.026
27	1081554	1087770	Golgi SNAP receptor complex member 2	0.026
27	1111810	1122644	Protein Wnt	0.026
27	1136146	1157434	wingless-type MMTV integration site family, member 3	0.026
27	1159103	1215306	vesicle-fusing ATPase	0.026
27	18285	19232	olfactory receptor, family 6, subfamily A, member 2	0.026
27	1768088	1844661	acid-sensing (proton-gated) ion channel 2	0.074
27	2357455	2399073	tousled-like kinase 2	0.073
27	4505836	4644100	Eukaryotic translation initiation factor 1	0.039
27	4618778	4622608	keratin, type I cytoskeletal 14	0.039
27	4645878	4646526	gastrin/cholecystokinin-like peptide precursor	0.039
27	4647054	4655682	huntingtin-associated protein 1	0.039
27	4658140	4665314	Plakoglobin	0.039
27	4674822	4678761	leprecan-like 4	0.039
27	4679153	4685639	FK506 binding protein 10, 65 kDa	0.039
27	4685681	4690379	Cytosolic 5'-nucleotidase III-like protein	0.039
27	4698639	4700783	kelch-like family member 11	0.039
27	4701712	4720355	ATP-citrate synthase	0.039
27	4733938	4738865	2',3'-cyclic nucleotide 3' phosphodiesterase	0.039
27	4739049	4758336	dnaJ homolog subfamily C member 7	0.039
27	4758782	4759794	NF-kappa-B inhibitor-interacting Ras-like protein 2	0.039
27	4822592	4826644	DEXH (Asp-Glu-X-His) box polypeptide 58	0.039
27	4827918	4832197	histone acetyltransferase KAT2A	0.039
27	4837933	4841685	Ras-related protein Rab-5C	0.039
27	4847654	4856867	potassium voltage-gated channel, subfamily H (eag-related), member 4	0.039

27	4857256	4858313	hypocretin (orexin) neuropeptide precursor	0.039
27	4871019	4873562	GH3 domain containing	0.039
27	4875164	4886606	signal transducer and activator of transcription 5B	0.039
27	4896267	4907552	Signal transducer and activator of transcription 3	0.039
27	4913996	4926852	polymerase I and transcript release factor	0.039
27	4929945	4958104	V-type proton ATPase 116 kDa subunit a isoform 1	0.039
27	4962219	4963268	17-beta-hydroxysteroid dehydrogenase	0.039
27	4963812	4965610	CoA synthase	0.039
27	4967021	4970406	max-like protein X	0.039
27	4970565	4973603	PSMC3 interacting protein	0.039
27	4982347	4989442	tubulin, gamma 1	0.039
27	4990263	4995457	pleckstrin homology domain containing, family H (with MyTH4 domain) member 3	0.039
27	5003802	5012710	contactin associated protein 1	0.039
27	5030186	5032034	receptor activity-modifying protein 2 precursor	0.039
27	5039150	5051893	WNK lysine deficient protein kinase 4	0.039
27	5055739	5060519	beclin-1	0.039
27	5060694	5067398	Proteasome activator complex subunit 3	0.039
27	5095037	5098091	RUN domain containing 1	0.039
27	5099248	5101501	60S ribosomal protein L27	0.039
27	5102844	5105307	interferon-induced protein 35	0.039
27	5126612	5140086	rho-related GTP-binding protein RhoN	0.039
27	5150701	5171059	breast cancer 1, early onset	0.039
27	5171677	5189435	neighbor of BRCA1 gene 1	0.039
27	5000132	5001181	chemokine (C-C motif) receptor 10	0.039
27	5173809	5173871	Neighbour of BRCA1 gene 2 converved region	0.039
28	509129	534490	heterogeneous nuclear ribonucleoprotein M	0.054
28	558963	564318	U6 snRNA-associated Sm-like protein LSm7	0.054
28	564384	599425	signal peptide peptidase-like 2B precursor	0.054
28	627972	628532	translocase of inner mitochondrial membrane 13 homolog (yeast)	0.054
28	636058	661825	lamin-B2	0.054

28	676818	677614	60S ribosomal protein L36	0.054
28	678763	694768	lon peptidase 1, mitochondrial	0.054
28	701484	724473	solute carrier family 1 (high affinity aspartate/glutamate transporter), member 6	0.054
28	746840	778361	RAN binding protein 3	0.054
28	789271	792789	kelch-like family member 33	0.054
28	803320	808851	butyrophilin subfamily 1 member A1 precursor	0.054
28	832292	846222	ras-related protein Rab-11B	0.054
28	877452	886036	KN motif and ankyrin repeat domains 3	0.054
28	899423	901661	anti-dorsalizing morphogenetic protein precursor	0.054
28	932572	950125	ELAV-like protein 1	0.054
28	1630487	1705053	long-chain-fatty-acid--CoA ligase ACSBG2	0.051
28	1650455	1678571	myeloid/lymphoid or mixed-lineage leukemia (trithorax homolog, Drosophila); translocated to, 1	0.051
28	1712018	1720854	Acidic leucine-rich nuclear phosphoprotein 32 family member B	0.051
28	1729378	1742502	myosin-If	0.051
28	1745352	1791963	ADAM metallopeptidase with thrombospondin type 1 motif, 10	0.051
28	1808517	1817532	Tyrosine-protein kinase	0.051
28	1846964	1848279	actin-like 9	0.051
28	1851800	1862161	mucin 16, cell surface associated	0.051
28	1930170	1932508	Ornithine decarboxylase antizyme 1	0.051
28	1956623	1960825	muellerian-inhibiting factor precursor	0.051
28	1962063	1966197	splicing factor 3A subunit 2	0.051
28	1967285	1973793	Pleckstrin homology domain-containing family J member 1	0.051
28	1978406	2033934	DOT1-like histone H3K79 methyltransferase	0.051
Z	9084930	9095262	carbonic anhydrase IX	0.068
Z	11585813	11602028	glial cell derived neurotrophic factor	0.001
Z	11778982	11853316	EGF-like, fibronectin type III and laminin G domains	0.001
Z	11866374	11907285	leukemia inhibitory factor receptor precursor	0.001
Z	12008448	12039786	oncostatin M receptor	0.001
Z	12046216	12121536	RPTOR independent companion of MTOR, complex 2	0.001
Z	12129418	12192609	FYN-binding protein	0.001

Z	15630610	15707360	integrin alpha-1 precursor	0.064
Z	15721124	15783248	integrin, alpha 2 (CD49B, alpha 2 subunit of VLA-2 receptor)	0.064
Z	18712425	18733642	DEP domain-containing protein 1B	0.053
Z	18735256	18767868	elongation of very long chain fatty acids protein 7	0.053
Z	18829576	18879586	mimitin, mitochondrial precursor	0.053
Z	18876476	18953581	Small integral membrane protein 15	0.053
Z	18687376	18687619	Prostate androgen-regulated transcript 1 conserved region 2	0.053
Z	20013551	20051436	regulator of G-protein signaling 7 binding protein	0.008
Z	20070886	20186949	CWC27 spliceosome-associated protein homolog (S. cerevisiae)	0.008
Z	36402033	36535271	nuclear receptor ROR-beta	0.044
Z	36568084	36656370	transient receptor potential cation channel, subfamily M, member 6	0.044
Z	40679500	40686431	Iron-sulfur cluster assembly 1 homolog, mitochondrial	0.062
Z	44205924	44207300	nuclear factor interleukin-3-regulated protein	0.000
Z	51426184	51474648	leucyl/cystinyl aminopeptidase	0.065
Z	55091689	55091845	TUC338	0.000
Z	62474981	62580840	versican core protein precursor	0.016
Z	63601953	63729018	Ras protein-specific guanine nucleotide-releasing factor 2	0.000
Z	63759855	63876936	mutS homolog 3 (E. coli)	0.000
Z	63876645	63893410	dihydrofolate reductase	0.000
Z	68346670	68380646	intraflagellar transport 74 homolog (Chlamydomonas)	0.000
Z	68352558	68358247	leucine rich repeat containing 19	0.000
Z	68386224	68425628	TEK tyrosine kinase, endothelial	0.000
Z	68486091	68486519	MOB kinase activator 3B	0.000
