# Supplementary material for: Population Genomic Analyses Based on 1 Million SNPs in Commercial Egg Layers
Source: PLoS One. 2014 Apr 16;9(4):e94509. doi: 10.1371/journal.pone.0094509 (PMC3989219; doi:10.1371/journal.pone.0094509)
Supplement: Table S4 — List of genes in upper 1% FST distribution in comparision of brown layers and white layers. (PDF) [file pone.0094509.s004.pdf]

Table S4. List of genes in upper 1% $F_{ST}$ distribution in comparision of brown layers and white layers.

| Chr | Start | End | Description | Fst |
|---|---|---|---|---|
| 1 | 9325096 | 9459193 | semaphorin-3E precursor | 0.686 |
| 1 | 9561909 | 9881531 | piccolo presynaptic cytomatrix protein | 0.686 |
| 1 | 14213411 | 14241547 | phosphatidylinositol-4,5-bisphosphate 3-kinase catalytic subunit gamma isoform | 0.713 |
| 1 | 14328390 | 14346800 | protein kinase, cAMP-dependent, regulatory, type II, beta | 0.713 |
| 1 | 14356759 | 14367631 | HMG box-containing protein 1 | 0.713 |
| 1 | 14369255 | 14553213 | conserved oligomeric Golgi complex subunit 5 | 0.713 |
| 1 | 14553188 | 14563663 | dihydrouridine synthase 4-like (S. cerevisiae) | 0.713 |
| 1 | 14567866 | 14591310 | B-cell receptor-associated protein 29 | 0.713 |
| 1 | 14621478 | 14632510 | E3 ubiquitin-protein ligase Hakai | 0.713 |
| 1 | 14632208 | 14642808 | chloride anion exchanger | 0.713 |
| 1 | 14666134 | 14679885 | dihydrolipoyl dehydrogenase, mitochondrial | 0.713 |
| 1 | 14681577 | 14715087 | Laminin subunit beta-1 | 0.713 |
| 1 | 14777556 | 14800582 | endoplasmic reticulum-Golgi intermediate compartment protein 2 | 0.713 |
| 1 | 14916517 | 15054085 | solute carrier family 2 (facilitated glucose transporter), member 13 | 0.713 |
| 1 | 24794262 | 24882798 | hepatocyte growth factor receptor precursor | 0.507 |
| 1 | 25847642 | 26191414 | FoxP2; Uncharacterized protein | 0.753 |
| 1 | 26299344 | 26325360 | protein phosphatase 1, regulatory subunit 3A | 0.753 |
| 1 | 28473454 | 28508702 | calcium-independent phospholipase A2-gamma | 0.568 |
| 1 | 31999958 | 32082280 | monocarboxylate transporter 2 | 0.526 |
| 1 | 35865662 | 36009723 | protein tyrosine phosphatase, receptor type, R | 0.607 |
| 1 | 40131212 | 40428161 | protein tyrosine phosphatase, receptor type, f polypeptide (PTPRF), interacting protein (liprin), alpha 2 | 0.560 |
| 1 | 40619495 | 40678658 | methyltransferase like 25 | 0.560 |
| 1 | 42095834 | 42213439 | Alpha-1,3-mannosyl-glycoprotein 4-beta-N-acetylglucosaminyltransferase C | 0.636 |
| 1 | 43171637 | 43175978 | dual specificity protein phosphatase 6 | 0.642 |
| 1 | 43207669 | 43254712 | WD repeat-containing protein 51B | 0.642 |
| 1 | 43273706 | 43305815 | plasma membrane calcium-transporting ATPase 1 | 0.642 |
| 1 | 46488737 | 46510905 | lamina-associated polypeptide 2, isoform beta | 0.568 |

| | | | | |
|---|---|---|---|---|
| 1 | 46514910 | 46522801 | phosphate carrier protein, mitochondrial | 0.568 |
| 1 | 51257450 | 51266551 | ras-related C3 botulinum toxin substrate 2 | 0.634 |
| 1 | 51269724 | 51274129 | somatostatin receptor type 3 | 0.634 |
| 1 | 51336790 | 51344019 | potassium channel tetramerization domain containing 17 | 0.634 |
| 1 | 51349989 | 51355027 | Sulfurtransferase | 0.634 |
| 1 | 51355166 | 51362344 | thiosulfate sulfurtransferase | 0.634 |
| 1 | 51419978 | 51430209 | neutrophil cytosol factor 4 | 0.634 |
| 1 | 51455040 | 51463393 | Parvalbumin, muscle | 0.634 |
| 1 | 51573280 | 51580672 | thioredoxin, mitochondrial | 0.634 |
| 1 | 51598550 | 51666512 | myosin-9 | 0.634 |
| 1 | 51759800 | 51835231 | RNA binding protein, fox-1 homolog (C. elegans) 2 | 0.634 |
| 1 | 51849854 | 51853554 | Myoglobin | 0.634 |
| 1 | 51939574 | 51947830 | DNA replication licensing factor MCM5 | 0.634 |
| 1 | 51949101 | 51955226 | heme oxygenase 1 | 0.634 |
| 1 | 51962485 | 51983162 | Target of Myb protein 1 | 0.634 |
| 1 | 51511551 | 51511715 | TUC338 | 0.634 |
| 1 | 52524419 | 52799322 | glycosyltransferase-like protein LARGE1 | 0.562 |
| 1 | 59065375 | 59100295 | dynamin-1-like protein | 0.655 |
| 1 | 60438890 | 60496703 | beta-1,4-N-acetyl-galactosaminyl transferase 3 | 0.529 |
| 1 | 63429485 | 63435977 | microsomal glutathione S-transferase 1 | 0.601 |
| 1 | 66735234 | 66851127 | alpha-N-acetylneuraminide alpha-2,8-sialyltransferase | 0.621 |
| 1 | 68357089 | 68442009 | liprin-beta-1 | 0.580 |
| 1 | 68516162 | 68549653 | ADP-ribosylation factor GTPase-activating protein 3 | 0.580 |
| 1 | 68556334 | 68619317 | protein kinase C and casein kinase substrate in neurons protein 2 isoform 1 | 0.580 |
| 1 | 68626823 | 68645266 | tubulin tyrosine ligase-like family, member 1 | 0.580 |
| 1 | 68667862 | 68672968 | malonyl CoA:ACP acyltransferase (mitochondrial) | 0.580 |
| 1 | 68683782 | 68704548 | tubulin--tyrosine ligase-like protein 12 | 0.580 |
| 1 | 68715574 | 68928375 | signal peptide, CUB domain, EGF-like 1 | 0.580 |
| 1 | 68833800 | 68833975 | TUC338 | 0.580 |
| 1 | 69852304 | 69938056 | proline-rich protein 5 | 0.551 |

| | | | | |
|---|---|---|---|---|
| 1 | 70002408 | 70079769 | rho GTPase-activating protein 8 | 0.551 |
| 1 | 70098157 | 70154426 | PHD finger protein 21B | 0.551 |
| 1 | 72902806 | 73148067 | fatty acyl CoA reductase 2 | 0.737 |
| 1 | 84610640 | 84660808 | interphotoreceptor matrix proteoglycan 2 precursor | 0.640 |
| 1 | 84665305 | 84703930 | SUMO1/sentrin specific peptidase 7 | 0.640 |
| 1 | 84714811 | 84724364 | PEST proteolytic signal-containing nuclear protein | 0.640 |
| 1 | 84747544 | 84751604 | ribosomal protein L24 | 0.640 |
| 1 | 84799655 | 84816004 | NF-kappa-B inhibitor zeta | 0.640 |
| 1 | 87878151 | 87933199 | pleckstrin homology-like domain, family B, member 2 | 0.569 |
| 1 | 91358735 | 91424437 | prostaglandin F2 receptor inhibitor | 0.551 |
| 1 | 1E+08 | 1E+08 | neural cell adhesion molecule 2 | 0.587 |
| 1 | 1.08E+08 | 1.08E+08 | Down syndrome cell adhesion molecule | 0.599 |
| 1 | 1.14E+08 | 1.15E+08 | dystrophin | 0.599 |
| 1 | 1.17E+08 | 1.17E+08 | DNA polymerase | 0.672 |
| 1 | 1.17E+08 | 1.18E+08 | phosphate cytidylyltransferase 1, choline, beta | 0.672 |
| 1 | 1.17E+08 | 1.17E+08 | Small Cajal body specific RNA 24 | 0.672 |
| 1 | 1.19E+08 | 1.2E+08 | SH3 domain-containing kinase-binding protein 1 | 0.510 |
| 1 | 1.24E+08 | 1.24E+08 | chloride channel, voltage-sensitive 4 | 0.575 |
| 1 | 1.45E+08 | 1.45E+08 | UDP-glucose:glycoprotein glucosyltransferase 2 precursor | 0.514 |
| 1 | 1.47E+08 | 1.47E+08 | glypican 5 | 0.656 |
| 1 | 1.49E+08 | 1.49E+08 | SLIT and NTRK-like family, member 5 | 0.821 |
| 1 | 1.52E+08 | 1.52E+08 | NEDD4 family-interacting protein 2 | 0.931 |
| 1 | 1.52E+08 | 1.52E+08 | RNA binding motif protein 26 | 0.931 |
| 1 | 1.52E+08 | 1.52E+08 | protein sprouty homolog 2 | 0.931 |
| 1 | 1.54E+08 | 1.54E+08 | ubiquitin carboxyl-terminal hydrolase isozyme L3 | 0.539 |
| 1 | 1.54E+08 | 1.54E+08 | TBC1 domain family, member 4 | 0.539 |
| 1 | 1.55E+08 | 1.55E+08 | Kruppel-like factor 12 | 0.539 |
| 1 | 1.62E+08 | 1.62E+08 | diaphanous-related formin 3 | 0.623 |
| 1 | 1.64E+08 | 1.64E+08 | Small nucleolar RNA U2-30 | 0.633 |
| 1 | 1.64E+08 | 1.64E+08 | Small nucleolar RNA U2-19 | 0.633 |

| | | | | |
|---|---|---|---|---|
| 1 | 1.65E+08 | 1.65E+08 | von Willebrand factor A domain containing 8 | 0.683 |
| 1 | 1.66E+08 | 1.66E+08 | diacylglycerol kinase, eta | 0.683 |
| 1 | 1.67E+08 | 1.67E+08 | family with sequence similarity 194, member B | 0.600 |
| 1 | 1.67E+08 | 1.67E+08 | Spermatid-associated protein | 0.600 |
| 1 | 1.67E+08 | 1.67E+08 | siah E3 ubiquitin protein ligase family member 3 | 0.600 |
| 1 | 1.67E+08 | 1.67E+08 | plastin-2 | 0.600 |
| 1 | 1.67E+08 | 1.67E+08 | leucine rich repeat containing 63 | 0.600 |
| 1 | 1.67E+08 | 1.67E+08 | KIAA0226-like | 0.600 |
| 1 | 1.68E+08 | 1.68E+08 | esterase D | 0.600 |
| 1 | 1.68E+08 | 1.68E+08 | 5-hydroxytryptamine (serotonin) receptor 2A, G protein-coupled | 0.600 |
| 1 | 1.68E+08 | 1.68E+08 | succinate-CoA ligase, ADP-forming, beta subunit | 0.600 |
| 1 | 1.68E+08 | 1.68E+08 | integral membrane protein 2B | 0.600 |
| 1 | 1.68E+08 | 1.68E+08 | retinoblastoma-associated protein | 0.600 |
| 1 | 1.68E+08 | 1.68E+08 | regulator of chromosome condensation (RCC1) and BTB (POZ) domain containing protein 2 | 0.600 |
| 1 | 1.68E+08 | 1.68E+08 | fibronectin type-III domain-containing protein 3a | 0.600 |
| 1 | 1.68E+08 | 1.68E+08 | motilin receptor | 0.600 |
| 1 | 1.68E+08 | 1.69E+08 | calcium binding protein 39-like | 0.600 |
| 1 | 1.75E+08 | 1.75E+08 | microtubule associated tumor suppressor candidate 2 | 0.549 |
| 1 | 1.76E+08 | 1.76E+08 | cyclin-dependent kinase 8 | 0.566 |
| 1 | 1.76E+08 | 1.76E+08 | ring finger protein (C3H2C3 type) 6 | 0.566 |
| 1 | 1.76E+08 | 1.76E+08 | ATPase, aminophospholipid transporter, class I, type 8A, member 2 | 0.566 |
| 1 | 1.77E+08 | 1.77E+08 | spastic ataxia of Charlevoix-Saguenay (sacsin) | 0.581 |
| 1 | 1.78E+08 | 1.78E+08 | glia-activating factor | 0.581 |
| 1 | 1.85E+08 | 1.85E+08 | solute carrier family 36 (proton/amino acid symporter), member 4 | 0.741 |
| 1 | 1.91E+08 | 1.92E+08 | teneurin transmembrane protein 4 | 0.711 |
| 1 | 1.92E+08 | 1.92E+08 | asparaginyl-tRNA synthetase 2, mitochondrial (putative) | 0.711 |
| 1 | 1.93E+08 | 1.93E+08 | tsukushin precursor | 0.593 |
| 2 | 5408261 | 5613834 | sodium channel, voltage-gated, type V, alpha subunit | 0.561 |
| 2 | 13816140 | 13922505 | neuropilin-1 precursor | 0.600 |
| 2 | 13975831 | 14017232 | integrin beta-1 precursor | 0.600 |

| | | | | |
|---|---|---|---|---|
| 2 | 13771061 | 13771230 | TUC338 | 0.600 |
| 2 | 19881822 | 19979217 | ras suppressor protein 1 | 0.581 |
| 2 | 24256193 | 24345252 | calcium-binding mitochondrial carrier protein Aralar2 | 0.525 |
| 2 | 24450646 | 24457181 | split hand/foot malformation (ectrodactyly) type 1 | 0.525 |
| 2 | 45255770 | 45389575 | cytoplasmic linker associated protein 2 | 0.535 |
| 2 | 45413332 | 45443733 | programmed cell death 6-interacting protein | 0.535 |
| 2 | 50484818 | 50543299 | cyclin-dependent kinase 13 | 0.751 |
| 2 | 50564486 | 50571264 | M-phase specific PLK1 interacting protein | 0.751 |
| 2 | 57910351 | 57932198 | glycosylphosphatidylinositol specific phospholipase D1 | 0.632 |
| 2 | 62603034 | 62615535 | phosphatase and actin regulator 1 | 0.531 |
| 2 | 67611323 | 67673626 | myosin light chain kinase family, member 4 | 0.542 |
| 2 | 67719974 | 67729598 | serpin B6 | 0.542 |
| 2 | 67730365 | 67739883 | Heterochromatin-associated protein MENT | 0.542 |
| 2 | 67745615 | 67751998 | Heterochromatin-associated protein MENT | 0.542 |
| 2 | 67753130 | 67763712 | serpin peptidase inhibitor, clade B (ovalbumin), member 2 | 0.542 |
| 2 | 67771785 | 67779357 | Ovalbumin | 0.542 |
| 2 | 67790408 | 67796318 | ovalbumin-related protein Y | 0.542 |
| 2 | 74276764 | 74466274 | Cadherin; Uncharacterized protein | 0.632 |
| 2 | 76311201 | 76496830 | trio Rho guanine nucleotide exchange factor | 0.518 |
| 2 | 78393973 | 78403910 | T-complex protein 1 subunit epsilon | 0.614 |
| 2 | 78404319 | 78411897 | family with sequence similarity 173, member B | 0.614 |
| 2 | 79220949 | 79424359 | adenylate cyclase 2 (brain) | 0.541 |
| 2 | 80447666 | 80505627 | von Willebrand factor C domain containing 2 | 0.633 |
| 2 | 80510980 | 80537096 | Zona pellucida-binding protein 1 | 0.633 |
| 2 | 80612647 | 80685641 | DNA-binding protein Ikaros | 0.633 |
| 2 | 80708843 | 80754977 | dopa decarboxylase (aromatic L-amino acid decarboxylase) | 0.633 |
| 2 | 83099424 | 83462516 | formin homology 2 domain containing 3 | 0.566 |
| 2 | 84812789 | 85025689 | molybdenum cofactor sulfurase | 0.699 |
| 2 | 86065381 | 86068294 | iroquois homeobox 4 | 0.766 |
| 2 | 97144112 | 97156128 | cell death activator CIDE-A | 0.550 |

| | | | | |
|---|---|---|---|---|
| 2 | 1.03E+08 | 1.03E+08 | RIO kinase 3 | 0.559 |
| 2 | 1.03E+08 | 1.03E+08 | Niemann-Pick disease, type C1 | 0.559 |
| 2 | 1.03E+08 | 1.03E+08 | ankyrin repeat domain 29 | 0.559 |
| 2 | 1.03E+08 | 1.03E+08 | laminin, alpha 3 | 0.559 |
| 2 | 1.03E+08 | 1.03E+08 | Oxysterol-binding protein | 0.559 |
| 2 | 1.08E+08 | 1.08E+08 | U6 spliceosomal RNA | 0.553 |
| 2 | 1.1E+08 | 1.1E+08 | V-type proton ATPase subunit H | 0.614 |
| 2 | 1.1E+08 | 1.1E+08 | Regulator of G-protein signaling 20 | 0.614 |
| 2 | 1.1E+08 | 1.1E+08 | transcription elongation factor A protein 1 | 0.614 |
| 2 | 1.11E+08 | 1.11E+08 | tyrosine-protein kinase Lyn | 0.529 |
| 2 | 1.17E+08 | 1.17E+08 | telomeric repeat-binding factor 1 | 0.523 |
| 2 | 1.23E+08 | 1.23E+08 | cyclic nucleotide gated channel beta 3 | 0.611 |
| 2 | 1.24E+08 | 1.24E+08 | nibrin | 0.532 |
| 2 | 1.24E+08 | 1.24E+08 | Calbindin | 0.532 |
| 2 | 1.27E+08 | 1.27E+08 | lysosomal protein transmembrane 4 beta | 0.523 |
| 2 | 1.28E+08 | 1.28E+08 | vacuolar protein sorting 13 homolog B (yeast) | 0.537 |
| 2 | 1.28E+08 | 1.28E+08 | cytochrome c oxidase subunit 6C | 0.537 |
| 2 | 1.37E+08 | 1.37E+08 | hyaluronan synthase 2 | 0.579 |
| 2 | 1.47E+08 | 1.47E+08 | t-SNARE domain containing 1 | 0.530 |
| 3 | 8094160 | 8384451 | anaplastic lymphoma receptor tyrosine kinase | 0.519 |
| 3 | 9521292 | 9539167 | protein pellino homolog 1 | 0.560 |
| 3 | 13302980 | 13394073 | 1-phosphatidylinositol-4,5-bisphosphate phosphodiesterase beta-4 | 0.628 |
| 3 | 20511715 | 20538253 | N-lysine methyltransferase SMYD2 | 0.580 |
| 3 | 28093815 | 28285437 | dishevelled associated activator of morphogenesis 2 | 0.716 |
| 3 | 28650945 | 28730284 | glucagon-like peptide 1 receptor precursor | 0.716 |
| 3 | 28737986 | 28830043 | dynein, axonemal, heavy chain 8 | 0.716 |
| 3 | 28867771 | 28979903 | BTB/POZ domain-containing protein 9 | 0.716 |
| 3 | 29337956 | 29424515 | MAM domain-containing glycosylphosphatidylinositol anchor protein 1 precursor | 0.716 |
| 3 | 29519203 | 29541046 | FtsJ methyltransferase domain containing 2 | 0.716 |
| 3 | 29557966 | 29574363 | probable alanyl-tRNA synthetase, mitochondrial | 0.716 |

| | | | | |
|---|---|---|---|---|
| 3 | 29217044 | 29217179 | TUC338 | 0.716 |
| 3 | 40939403 | 41033678 | SPARC related modular calcium binding 2 | 0.671 |
| 3 | 41121124 | 41158394 | sulfotransferase | 0.671 |
| 3 | 41160297 | 41172875 | dishevelled-binding antagonist of beta-catenin 2 | 0.671 |
| 3 | 41393868 | 41474285 | myeloid/lymphoid or mixed-lineage leukemia (trithorax homolog, Drosophila); translocated to, 4 | 0.671 |
| 3 | 41687479 | 41708127 | unc-93 homolog A (C. elegans) | 0.671 |
| 3 | 41711697 | 41713292 | tubulin tyrosine ligase-like family, member 2 | 0.671 |
| 3 | 41759132 | 41766064 | C-C chemokine receptor type 6 | 0.671 |
| 3 | 41783071 | 41803951 | FGFR1 oncogene partner | 0.671 |
| 3 | 41810099 | 41831299 | ribonuclease T2 precursor | 0.671 |
| 3 | 41958446 | 42114412 | ribosomal protein S6 kinase, 90kDa, polypeptide 2 | 0.671 |
| 3 | 42131326 | 42141404 | brain protein 44-like protein | 0.671 |
| 3 | 42233586 | 42241528 | brachyury protein | 0.671 |
| 3 | 42463951 | 42626252 | phosphodiesterase 10A | 0.671 |
| 3 | 43765827 | 44445298 | parkinson protein 2, E3 ubiquitin protein ligase (parkin) | 0.750 |
| 3 | 44773338 | 44826366 | cation-independent mannose-6-phosphate receptor precursor | 0.750 |
| 3 | 44874173 | 44881975 | T-complex protein 1 subunit alpha | 0.750 |
| 3 | 44881987 | 44889927 | acetyl-CoA acetyltransferase, cytosolic | 0.750 |
| 3 | 44923214 | 44929994 | superoxide dismutase | 0.750 |
| 3 | 45071689 | 45111718 | fibronectin type III domain containing 1 | 0.750 |
| 3 | 45215648 | 45566922 | utrophin | 0.750 |
| 3 | 45769623 | 45811385 | epilepsy, progressive myoclonus type 2A, Lafora disease (laforin) | 0.750 |
| 3 | 45868769 | 45921436 | SNF2 histone linker PHD RING helicase, E3 ubiquitin protein ligase | 0.750 |
| 3 | 45937157 | 46118743 | Glutamate receptor metabotropic 1 isoform f transcript variant 1 | 0.750 |
| 3 | 46182574 | 46256838 | androglobin | 0.750 |
| 3 | 46388675 | 46489008 | syntaxin binding protein 5 (tomosyn) | 0.750 |
| 3 | 46522593 | 46647011 | sterile alpha motif domain containing 5 | 0.750 |
| 3 | 47153562 | 47250156 | uronyl-2-sulfotransferase | 0.750 |
| 3 | 47394890 | 47423136 | peptidylprolyl isomerase (cyclophilin)-like 4 | 0.750 |
| 3 | 47425648 | 47442269 | uncharacterized protein C6orf72 homolog precursor | 0.750 |

| | | | | |
|---|---|---|---|---|
| 3 | 47446107 | 47459848 | Katanin p60 ATPase-containing subunit A1 | 0.750 |
| 3 | 47503537 | 47537193 | Protein-L-isoaspartate(D-aspartate) O-methyltransferase | 0.750 |
| 3 | 47538737 | 47606787 | low density lipoprotein receptor-related protein 11 | 0.750 |
| 3 | 44874859 | 44874995 | Small nucleolar RNA SNORA29 | 0.750 |
| 3 | 44880145 | 44880277 | Small nucleolar RNA SNORA20 | 0.750 |
| 3 | 48276571 | 48431441 | estrogen receptor | 0.758 |
| 3 | 48447032 | 48720659 | spectrin repeat containing, nuclear envelope 1 | 0.758 |
| 3 | 48776284 | 48795822 | VIP peptides isoform 2 preproprotein | 0.758 |
| 3 | 48910153 | 48981998 | Regulator of G-protein signaling 17 | 0.758 |
| 3 | 50465764 | 50754522 | AT rich interactive domain 1B (SWI1-like) | 0.731 |
| 3 | 53248977 | 53250435 | cbp/p300-interacting transactivator 2 | 0.511 |
| 3 | 54485370 | 54505293 | BCL2-associated transcription factor 1 | 0.555 |
| 3 | 58029862 | 58422557 | protein tyrosine phosphatase, receptor type, K | 0.530 |
| 3 | 63229377 | 63243518 | RWD domain containing 1 | 0.673 |
| 3 | 63261261 | 63269158 | uncharacterized protein LOC769904 | 0.673 |
| 3 | 63276143 | 63280172 | family with sequence similarity 26, member F | 0.673 |
| 3 | 63431647 | 63439612 | Collagen alpha-1(X) chain | 0.673 |
| 3 | 63472387 | 63519299 | fyn-related kinase | 0.673 |
| 3 | 64239389 | 64261685 | histone deacetylase 2 | 0.673 |
| 3 | 65392000 | 65429033 | tyrosine-protein kinase Fyn | 0.577 |
| 3 | 65542811 | 65609587 | REV3-like, polymerase (DNA directed), zeta, catalytic subunit | 0.577 |
| 3 | 65721376 | 65726791 | general transcription factor 3C polypeptide 6 | 0.577 |
| 3 | 65744041 | 65761269 | S-adenosylmethionine decarboxylase proenzyme | 0.577 |
| 3 | 66033229 | 66074352 | pre-mRNA-processing factor 17 | 0.577 |
| 3 | 66201896 | 66268774 | polyphosphoinositide phosphatase precursor | 0.577 |
| 3 | 66269578 | 66325155 | adenylate kinase 9 | 0.577 |
| 3 | 67757131 | 67863760 | Blimp-1 | 0.575 |
| 3 | 73318964 | 73462488 | Ephrin type-A receptor 7 | 0.667 |
| 3 | 74210444 | 74210526 | U6 spliceosomal RNA | 0.667 |
| 3 | 79802794 | 79856460 | Interphotoreceptor matrix proteoglycan 1 | 0.675 |

| | | | | |
|---|---|---|---|---|
| 3 | 79860207 | 79930603 | myosin-VI | 0.675 |
| 3 | 79966253 | 79995357 | SUMO1/sentrin specific peptidase 6 | 0.675 |
| 3 | 80071280 | 80164378 | filamin A interacting protein 1 | 0.675 |
| 3 | 80180051 | 80193545 | cell cycle control protein 50A | 0.675 |
| 3 | 80197965 | 80201301 | cytochrome c oxidase subunit 7A2, mitochondrial | 0.675 |
| 3 | 80212182 | 80312375 | collagen alpha-1(XII) chain precursor | 0.675 |
| 3 | 80775681 | 80855396 | CD109 molecule | 0.675 |
| 3 | 81337238 | 81638081 | regulating synaptic membrane exocytosis 1 | 0.578 |
| 3 | 86044771 | 86130756 | DNA primase large subunit | 0.540 |
| 3 | 96729661 | 96737554 | PQ loop repeat containing 3 | 0.721 |
| 3 | 96746492 | 96802174 | Rho-associated, coiled-coil containing protein kinase 2 | 0.721 |
| 3 | 96852537 | 96861622 | transcription factor E2F6 | 0.721 |
| 3 | 96906122 | 96965158 | growth regulation by estrogen in breast cancer 1 | 0.721 |
| 3 | 96971362 | 97044067 | Lipin 1; Uncharacterized protein | 0.721 |
| 3 | 97427885 | 97448910 | tribbles homolog 2 | 0.721 |
| 3 | 98343307 | 98504175 | neuroblastoma amplified sequence | 0.721 |
| 3 | 98505120 | 98526381 | ATP-dependent RNA helicase DDX1 | 0.721 |
| 3 | 98778414 | 98780948 | N-myc proto-oncogene protein | 0.721 |
| 3 | 99739837 | 99814889 | Visinin-like protein 1 | 0.551 |
| 3 | 1.04E+08 | 1.05E+08 | putative Polycomb group protein ASXL2 | 0.536 |
| 4 | 32207746 | 32286859 | doublecortin-like kinase 2 | 0.799 |
| 4 | 46222167 | 46249662 | Phosphatidate cytidylyltransferase | 0.553 |
| 4 | 49571259 | 49794271 | solute carrier family 4, sodium bicarbonate cotransporter, member 4 | 0.699 |
| 4 | 49817085 | 49822899 | vitamin D-binding protein precursor | 0.699 |
| 4 | 49832925 | 49847032 | neuropeptide FF receptor 2 | 0.699 |
| 4 | 49890856 | 49977356 | ADAM metallopeptidase with thrombospondin type 1 motif, 3 | 0.699 |
| 4 | 50153707 | 50226185 | ankyrin repeat domain 17 | 0.699 |
| 4 | 50253145 | 50269174 | serum albumin precursor | 0.699 |
| 4 | 50342296 | 50345374 | interleukin-8 precursor | 0.699 |
| 4 | 50355001 | 50359957 | Interleukin-8 | 0.699 |

| | | | | |
|---|---|---|---|---|
| 4 | 50470435 | 50669744 | ephrin type-A receptor 5 precursor | 0.699 |
| 4 | 50510247 | 50510326 | Small nucleolar RNA R12 | 0.699 |
| 4 | 55856553 | 55899875 | 2-hydroxyacylsphingosine 1-beta-galactosyltransferase precursor | 0.551 |
| 4 | 56052213 | 56091725 | arylsulfatase family, member J | 0.551 |
| 4 | 58208189 | 58447378 | netrin receptor UNC5C precursor | 0.534 |
| 4 | 59910288 | 60049717 | protein phosphatase 3, catalytic subunit, alpha isozyme | 0.616 |
| 4 | 69781392 | 69796531 | death domain containing 1 | 0.537 |
| 4 | 69799893 | 69923066 | ArfGAP with RhoGAP domain, ankyrin repeat and PH domain 2 | 0.537 |
| 4 | 75401346 | 75452145 | Ligand-dependent nuclear receptor corepressor-like protein | 0.732 |
| 4 | 75480300 | 75503309 | non-SMC condensin I complex, subunit G | 0.732 |
| 4 | 81411667 | 81420730 | alpha-2-macroglobulin receptor-associated protein precursor | 0.584 |
| 4 | 81465866 | 81491136 | docking protein 7 | 0.584 |
| 4 | 81495986 | 81536120 | HGF activator | 0.584 |
| 4 | 81642832 | 81659465 | Myb/SANT-like DNA-binding domain containing 1 | 0.584 |
| 4 | 81699702 | 81774162 | huntingtin | 0.584 |
| 4 | 81780294 | 81810730 | G protein-coupled receptor kinase 4 | 0.584 |
| 4 | 81861191 | 81918087 | alpha-adducin | 0.584 |
| 4 | 81924388 | 81959922 | SH3 domain-binding protein 2 | 0.584 |
| 4 | 82006422 | 82013592 | TNFAIP3-interacting protein 2 | 0.584 |
| 4 | 82049672 | 82097810 | family with sequence similarity 193, member A | 0.584 |
| 4 | 82143274 | 82159327 | RING finger protein 4 | 0.584 |
| 4 | 82408395 | 82444435 | max dimerization protein 4 | 0.584 |
| 4 | 82459166 | 82544586 | polymerase (DNA directed) nu | 0.584 |
| 4 | 81902213 | 81902370 | U1 spliceosomal RNA | 0.584 |
| 5 | 10010798 | 10123603 | inscuteable homolog (Drosophila) | 0.626 |
| 5 | 12238926 | 12267404 | receptor-type tyrosine-protein phosphatase eta | 0.755 |
| 5 | 13148583 | 13163279 | Insulin-like growth factor II | 0.537 |
| 5 | 13377709 | 13388547 | 39S ribosomal protein L23, mitochondrial | 0.537 |
| 5 | 13389730 | 13416288 | Troponin T, fast skeletal muscle isoforms | 0.537 |
| 5 | 13434000 | 13472383 | lymphocyte-specific protein 1 | 0.537 |

| | | | | |
|---|---|---|---|---|
| 5 | 13473898 | 13476632 | Troponin I, fast skeletal muscle | 0.537 |
| 5 | 17230853 | 17528495 | SH3 and multiple ankyrin repeat domains 2 | 0.553 |
| 5 | 17708714 | 17717820 | rhombotin-2 | 0.553 |
| 5 | 17763945 | 17795688 | caprin-1 | 0.553 |
| 5 | 22947434 | 22948426 | midkine precursor | 0.550 |
| 5 | 22949353 | 22992334 | diacylglycerol kinase zeta | 0.550 |
| 5 | 23071172 | 23192606 | PHD finger protein 21A | 0.550 |
| 5 | 23200237 | 23205076 | glycosyltransferase-like protein LARGE2 | 0.550 |
| 5 | 23227971 | 23241314 | mitogen-activated protein kinase 8 interacting protein 1 | 0.550 |
| 5 | 23244457 | 23264618 | cryptochrome-2 | 0.550 |
| 5 | 23265765 | 23269277 | GDP-fucose transporter 1 | 0.550 |
| 5 | 22944471 | 22945943 | muscarinic acetylcholine receptor M4 | 0.550 |
| 5 | 23959882 | 23993960 | inositol-trisphosphate 3-kinase A | 0.631 |
| 5 | 24865079 | 24890722 | vam6/Vps39-like protein | 0.648 |
| 5 | 24915661 | 24936853 | neutral alpha-glucosidase C | 0.648 |
| 5 | 24937025 | 24960748 | calpain-3 | 0.648 |
| 5 | 25000359 | 25015379 | synaptosomal-associated protein 23 | 0.648 |
| 5 | 25024800 | 25029487 | HAUS augmin-like complex subunit 2 | 0.648 |
| 5 | 26489235 | 26559259 | pecanex homolog (Drosophila) | 0.583 |
| 5 | 26586981 | 26631624 | mitogen-activated protein kinase kinase kinase 9 | 0.583 |
| 5 | 33101816 | 33216460 | serine/threonine-protein kinase D1 | 0.517 |
| 5 | 34375148 | 34959900 | neuronal PAS domain protein 3 | 0.680 |
| 5 | 35188533 | 35193787 | E2F-associated phosphoprotein | 0.680 |
| 5 | 35195367 | 35218018 | sorting nexin-6 | 0.680 |
| 5 | 35241369 | 35244169 | Cofilin-2 | 0.680 |
| 5 | 35253580 | 35299381 | bromodomain adjacent to zinc finger domain, 1A | 0.680 |
| 5 | 34864564 | 34864741 | TUC338 | 0.680 |
| 5 | 34463947 | 34464039 | Vault RNA | 0.680 |
| 5 | 38541886 | 38599635 | serine palmitoyltransferase 2 | 0.573 |
| 5 | 38619465 | 38632326 | alkylated DNA repair protein alkB homolog 1 | 0.573 |

| | | | | |
|---|---|---|---|---|
| 5 | 38634500 | 38651966 | SNW domain containing 1 | 0.573 |
| 5 | 38657380 | 38732532 | uncharacterized aarF domain-containing protein kinase 1 precursor | 0.573 |
| 5 | 39826229 | 39837879 | Iodothyronine deiodinase | 0.634 |
| 5 | 40042540 | 40090213 | thyrotropin receptor isoform 1 precursor | 0.634 |
| 5 | 40099534 | 40119234 | transcription initiation factor IIA subunit 1 | 0.634 |
| 5 | 40144828 | 40210981 | stonin 2 | 0.634 |
| 5 | 42319730 | 42344911 | spermatogenesis associated 7 | 0.612 |
| 5 | 48067831 | 48083159 | cholesterol 24-hydroxylase | 0.595 |
| 5 | 48159648 | 48241305 | echinoderm microtubule associated protein like 1 | 0.595 |
| 5 | 48312771 | 48378821 | ena/VASP-like protein | 0.595 |
| 5 | 48418077 | 48431424 | transcriptional repressor protein YY1 | 0.595 |
| 5 | 48456828 | 48465791 | solute carrier family 25, member 47 | 0.595 |
| 5 | 48490625 | 48546715 | WD repeat domain 25 | 0.595 |
| 5 | 48566385 | 48715173 | brain-enriched guanylate kinase-associated | 0.595 |
| 5 | 53874253 | 53882060 | small nuclear RNA activating complex, polypeptide 1, 43kDa | 0.603 |
| 5 | 53884053 | 53914606 | hypoxia-inducible factor 1-alpha | 0.603 |
| 5 | 58202924 | 58265575 | DDHD domain containing 1 | 0.622 |
| 5 | 58454254 | 58457967 | Bone morphogenetic protein 4 | 0.622 |
| 6 | 8142505 | 8170935 | cyclin-dependent kinase 1 | 0.519 |
| 6 | 11510433 | 11530618 | proactivator polypeptide precursor | 0.628 |
| 6 | 11543130 | 11545869 | carbohydrate sulfotransferase 3 | 0.628 |
| 6 | 11556862 | 11568617 | Histone H2A | 0.628 |
| 6 | 12928166 | 13365708 | calcium-activated potassium channel subunit alpha-1 | 0.546 |
| 6 | 19439617 | 19465235 | tankyrase-2 | 0.564 |
| 6 | 20586643 | 20593197 | cyclin J | 0.614 |
| 6 | 20638215 | 20730717 | B-cell linker protein | 0.614 |
| 6 | 20754954 | 20843012 | DNA nucleotidylexotransferase | 0.614 |
| 6 | 21051932 | 21053666 | VENT homeobox | 0.614 |
| 6 | 21074071 | 21135637 | kinase non-catalytic C-lobe domain (KIND) containing 1 | 0.614 |
| 7 | 3031382 | 3433912 | receptor tyrosine-protein kinase erbB-4 | 0.517 |

| | | | | |
|---|---|---|---|---|
| 7 | 4722132 | 4740447 | melanophilin | 0.543 |
| 7 | 16404276 | 16412287 | secernin 3 | 0.623 |
| 7 | 16412437 | 16433377 | Corepressor interacting with RBPJ 1 | 0.623 |
| 7 | 16463674 | 16548896 | Obg-like ATPase 1 | 0.623 |
| 7 | 17199467 | 17208239 | pyruvate dehydrogenase kinase, isozyme 1 | 0.639 |
| 7 | 17214612 | 17252865 | integrin alpha-6 precursor | 0.639 |
| 7 | 17770145 | 17966292 | myosin IIIB | 0.547 |
| 7 | 18583602 | 18668725 | serine threonine kinase 39 | 0.652 |
| 7 | 21293508 | 21321944 | Integrin beta | 0.594 |
| 7 | 21327981 | 21363749 | secretory phospholipase A2 receptor precursor | 0.594 |
| 7 | 26451247 | 26646791 | adenylate cyclase type 5 | 0.624 |
| 7 | 26653371 | 26673051 | protein-tyrosine phosphatase-like member B | 0.624 |
| 7 | 26674970 | 26859021 | myosin light chain kinase, smooth muscle | 0.624 |
| 7 | 30459507 | 30701723 | thrombospondin, type I, domain containing 7B | 0.539 |
| 7 | 31350097 | 31932431 | low density lipoprotein receptor-related protein 1 | 0.549 |
| 7 | 35234890 | 35264450 | G protein-activated inward rectifier potassium channel 1 | 0.525 |
| 8 | 822750 | 960453 | netrin G1 | 0.799 |
| 8 | 987129 | 1134223 | guanine nucleotide exchange factor VAV3 | 0.799 |
| 8 | 2165461 | 2220563 | serine/threonine-protein kinase Nek7 | 0.532 |
| 8 | 5890359 | 5957040 | acyl-CoA binding domain containing 6 | 0.637 |
| 9 | 2069219 | 2087633 | kelch-like 6 | 0.531 |
| 9 | 5411909 | 5436836 | retinol binding protein 1, cellular | 0.731 |
| 9 | 5566977 | 5861817 | calsyntenin 2 | 0.731 |
| 9 | 7800341 | 7823434 | plasminogen activator inhibitor type 1, member 2 precursor | 0.563 |
| 9 | 12913816 | 12999648 | fibroblast growth factor 12 | 0.629 |
| 9 | 13178354 | 13185135 | urotensin-2B precursor | 0.629 |
| 9 | 13186472 | 13199826 | osteocrin precursor | 0.629 |
| 9 | 20419761 | 20464371 | cholinesterase precursor | 0.605 |
| 9 | 23218003 | 23220395 | profilin-2 | 0.780 |
| 9 | 23226057 | 23254253 | E3 ubiquitin-protein ligase RNF13 | 0.780 |

| | | | | |
|---|---|---|---|---|
| 9 | 23254409 | 23259438 | COMM domain containing 2 | 0.780 |
| 9 | 23264511 | 23304671 | WW domain containing transcription regulator 1 | 0.780 |
| 9 | 23359472 | 23377519 | ceruloplasmin (ferroxidase) | 0.780 |
| 9 | 23410228 | 23425241 | glycogenin-1 | 0.780 |
| 10 | 3103512 | 3116708 | neuronal acetylcholine receptor subunit beta-4 precursor | 0.511 |
| 10 | 3121889 | 3127878 | Neuronal acetylcholine receptor subunit alpha-3 | 0.511 |
| 10 | 3952464 | 4034731 | vacuolar protein sorting 13 homolog C (S. cerevisiae) | 0.511 |
| 10 | 5072623 | 5098236 | Kruppel-like factor 13 | 0.666 |
| 10 | 5146745 | 5227651 | transient receptor potential cation channel, subfamily M, member 1 | 0.666 |
| 10 | 6434758 | 6619897 | disintegrin and metalloproteinase domain-containing protein 10 precursor | 0.671 |
| 10 | 6480269 | 6521283 | lipase, hepatic | 0.671 |
| 10 | 6572777 | 6596733 | aquaporin 9 | 0.671 |
| 10 | 6604818 | 6661741 | Retinal dehydrogenase 2 | 0.671 |
| 10 | 6816944 | 6857906 | cingulin-like 1 | 0.671 |
| 10 | 6903270 | 7058903 | transcription factor 12 | 0.671 |
| 10 | 19265373 | 19502893 | CTD small phosphatase-like protein 2 | 0.595 |
| 10 | 19310281 | 19330984 | protein CASC4 precursor | 0.595 |
| 11 | 4357663 | 4419746 | RPGRIP1-like | 0.540 |
| 11 | 4456387 | 4469147 | AKT-interacting protein | 0.540 |
| 11 | 10016612 | 10144234 | carbohydrate (N-acetylgalactosamine 4-0) sulfotransferase 8 | 0.644 |
| 11 | 10168414 | 10216329 | potassium channel tetramerization domain containing 15 | 0.644 |
| 11 | 10356455 | 10374076 | protein LSM14 homolog A | 0.644 |
| 11 | 10376830 | 10403785 | KIAA0355 | 0.644 |
| 11 | 10450875 | 10471700 | glucose-6-phosphate isomerase | 0.644 |
| 11 | 10610357 | 10619189 | SUMO-activating enzyme subunit 2 | 0.644 |
| 11 | 10624075 | 10629096 | programmed cell death protein 2-like | 0.644 |
| 11 | 10773808 | 10782445 | carbonic anhydrase VII | 0.644 |
| 11 | 10786610 | 10798981 | NEDD8-activating enzyme E1 regulatory subunit | 0.644 |
| 11 | 10817084 | 10841597 | cytoplasmic dynein 1 light intermediate chain 2 | 0.644 |
| 11 | 10894386 | 10903372 | CKLF-like MARVEL transmembrane domain-containing protein 3 | 0.644 |

| | | | | |
|---|---|---|---|---|
| 11 | 10906480 | 10917656 | thymidine kinase 2, mitochondrial | 0.644 |
| 11 | 10927995 | 10964663 | protein BEAN1 | 0.644 |
| 11 | 10982217 | 11011468 | cadherin-5 precursor | 0.644 |
| 11 | 11582884 | 11662192 | cadherin-11 precursor | 0.644 |
| 11 | 10763464 | 10765059 | pyruvate dehyrogenase phosphatase catalytic subunit 2 | 0.644 |
| 11 | 12288497 | 12432361 | cadherin-8 | 0.661 |
| 11 | 12638991 | 12639143 | TUC338 | 0.661 |
| 11 | 18267773 | 18286192 | transcription factor 25 (basic helix-loop-helix) | 0.667 |
| 11 | 18290821 | 18292152 | tubulin beta-4 chain | 0.667 |
| 11 | 18293266 | 18296749 | differentially expressed in FDCP 8 homolog (mouse) | 0.667 |
| 11 | 18300555 | 18308190 | growth arrest-specific 8 | 0.667 |
| 11 | 18308197 | 18309804 | urate (hydroxyiso-) hydrolase, pseudogene | 0.667 |
| 11 | 18310977 | 18316940 | cadherin 3, type 1, P-cadherin (placental) | 0.667 |
| 11 | 18317572 | 18326276 | cadherin-1 precursor | 0.667 |
| 11 | 18355336 | 18357463 | hyaluronan synthase 3 | 0.667 |
| 11 | 18358802 | 18360418 | CTF8, chromosome transmission fidelity factor 8 homolog (S. cerevisiae) | 0.667 |
| 11 | 18365413 | 18370296 | syntrophin, beta 2 (dystrophin-associated protein A1, 59kDa, basic component 2) | 0.667 |
| 11 | 18378690 | 18380054 | 60S ribosome subunit biogenesis protein NIP7 homolog | 0.667 |
| 11 | 18383995 | 18391771 | Telomeric repeat-binding factor 2 | 0.667 |
| 11 | 18396032 | 18407915 | outer mitochondrial membrane cytochrome b5 | 0.667 |
| 11 | 18415930 | 18474957 | nuclear factor of activated T-cells 5 | 0.667 |
| 11 | 18482780 | 18484568 | NAD(P)H dehydrogenase, quinone 1 | 0.667 |
| 11 | 18487655 | 18519315 | NEDD4-like E3 ubiquitin-protein ligase WWP2 | 0.667 |
| 11 | 18520346 | 18526403 | proteasome (prosome, macropain) 26S subunit, non-ATPase, 7 | 0.667 |
| 11 | 19150949 | 19161411 | pre-mRNA-splicing factor ATP-dependent RNA helicase PRP16 | 0.667 |
| 11 | 18287877 | 18288821 | melanocyte-stimulating hormone receptor | 0.667 |
| 12 | 13983652 | 14275283 | membrane associated guanylate kinase, WW and PDZ domain containing 1 | 0.559 |
| 12 | 18847535 | 19073767 | glutamate receptor, metabotropic 7 | 0.581 |
| 13 | 4187413 | 4738485 | teneurin-2 isoform 1 | 0.685 |
| 13 | 10737034 | 10764716 | IL2-inducible T-cell kinase | 0.565 |

| | | | | |
|---|---|---|---|---|
| 13 | 13243833 | 13260270 | glutamine-fructose-6-phosphate transaminase 2 | 0.528 |
| 13 | 13261655 | 13281786 | Mitogen-activated protein kinase 9 | 0.528 |
| 13 | 16744797 | 16844189 | Rho GTPase-activating protein 26 | 0.567 |
| 14 | 6602197 | 6621651 | leucine carboxyl methyltransferase 1 | 0.598 |
| 15 | 2912110 | 3202073 | syntaxin-2 | 0.595 |
| 15 | 3051055 | 3169587 | G protein-coupled receptor 133 | 0.595 |
| 15 | 3179902 | 3184192 | GTP-binding nuclear protein Ran | 0.595 |
| 15 | 3029980 | 3030156 | TUC338 | 0.595 |
| 15 | 4855484 | 4908313 | dynein, axonemal, heavy chain 10 | 0.636 |
| 15 | 4909039 | 4924155 | V-type proton ATPase 116 kDa subunit a isoform 2 | 0.636 |
| 15 | 4933001 | 4937788 | general transcription factor IIH, polypeptide 3, 34kDa | 0.636 |
| 15 | 4941748 | 4948314 | ATP-dependent RNA helicase DDX55 | 0.636 |
| 15 | 4977536 | 4979234 | small nuclear ribonucleoprotein 35kDa (U11/U12) | 0.636 |
| 15 | 4986361 | 4990790 | SET domain containing (lysine methyltransferase) 8 | 0.636 |
| 15 | 5002524 | 5026119 | protein strawberry notch homolog 1 | 0.636 |
| 15 | 5038370 | 5051012 | cyclin-dependent kinase 2-associated protein 1 | 0.636 |
| 15 | 5053460 | 5077681 | M-phase phosphoprotein 9 | 0.636 |
| 15 | 5080304 | 5188058 | phosphatidylinositol transfer protein, membrane-associated 2 | 0.636 |
| 15 | 5192449 | 5195746 | ADP-ribosylation factor-like protein 6-interacting protein 4 | 0.636 |
| 15 | 5208507 | 5219304 | ATP-binding cassette, sub-family B (MDR/TAP), member 9 | 0.636 |
| 15 | 5243010 | 5257347 | huntingtin-interacting protein 1-related protein | 0.636 |
| 15 | 5264075 | 5270199 | Density-regulated protein | 0.636 |
| 15 | 5297891 | 5339287 | Sarcoplasmic/endoplasmic reticulum calcium ATPase 2 | 0.636 |
| 15 | 6301498 | 6347392 | HECT domain containing E3 ubiquitin protein ligase 4 | 0.514 |
| 18 | 297153 | 334809 | myosin, heavy chain 13, skeletal muscle | 0.662 |
| 18 | 2569645 | 2666638 | protoheme IX farnesyltransferase, mitochondrial | 0.544 |
| 18 | 4721036 | 4751867 | SAP30 binding protein | 0.662 |
| 18 | 4750809 | 4787690 | RecQ protein-like 5 | 0.662 |
| 18 | 4763901 | 4774954 | uncharacterized protein LOC770371 | 0.662 |
| 18 | 6953318 | 6958961 | importin subunit alpha-2 | 0.645 |

| | | | | |
|---|---|---|---|---|
| 18 | 6970301 | 7017419 | bromodomain PHD finger transcription factor | 0.645 |
| 18 | 7017750 | 7029054 | nucleolar protein 11 | 0.645 |
| 18 | 7205116 | 7244436 | voltage-dependent calcium channel gamma-4 subunit | 0.645 |
| 18 | 7895218 | 7917904 | axin-2 | 0.598 |
| 18 | 7997936 | 8029708 | regulator of G-protein signaling 9 | 0.598 |
| 18 | 8091453 | 8108190 | arylsulfatase G | 0.598 |
| 18 | 8136004 | 8149977 | cAMP-dependent protein kinase type I-alpha regulatory subunit | 0.598 |
| 18 | 8153051 | 8162773 | family with sequence similarity 20, member A | 0.598 |
| 18 | 8232456 | 8254284 | ATP-binding cassette, sub-family A (ABC1), member 5 | 0.598 |
| 18 | 8260526 | 8295962 | mitogen-activated protein kinase kinase 6 | 0.598 |
| 18 | 8467064 | 8473808 | inward rectifier potassium channel 2 | 0.598 |
| 19 | 7382617 | 7394940 | ribosomal protein S6 kinase beta-1 | 0.575 |
| 20 | 593577 | 597282 | TBC/LysM-associated domain containing 2 | 0.667 |
| 20 | 597387 | 623826 | SAM domain and HD domain-containing protein 1 | 0.667 |
| 20 | 626685 | 647924 | retinoblastoma-like 1 (p107) | 0.667 |
| 20 | 650631 | 698519 | chromodomain helicase DNA binding protein 6 | 0.667 |
| 20 | 731978 | 793938 | ral GTPase-activating protein subunit beta | 0.667 |
| 20 | 835504 | 867279 | NDRG family member 3 | 0.667 |
| 20 | 869594 | 872258 | Src-like-adaptor 2 | 0.667 |
| 20 | 875509 | 927530 | PHD finger protein 20 | 0.667 |
| 20 | 1049215 | 1303743 | copine I | 0.667 |
| 20 | 1135479 | 1137453 | reactive oxygen species modulator 1 | 0.667 |
| 20 | 1137517 | 1148952 | cysteine desulfurase, mitochondrial | 0.667 |
| 20 | 1438852 | 1442860 | growth/differentiation factor 5 precursor | 0.667 |
| 20 | 1510312 | 1559445 | ubiquinol-cytochrome c reductase complex chaperone | 0.667 |
| 20 | 1565847 | 1590302 | family with sequence similarity 83, member C | 0.667 |
| 20 | 1591146 | 1598063 | eukaryotic translation initiation factor 6 | 0.667 |
| 20 | 1601528 | 1644296 | matrix metallopeptidase 24 (membrane-inserted) | 0.667 |
| 20 | 1643850 | 1862123 | adenosylhomocysteinase | 0.667 |
| 20 | 1667567 | 1673056 | dynein, light chain, roadblock-type 1 | 0.667 |

| | | | | |
|---|---|---|---|---|
| 20 | 5699881 | 5712513 | adenosine deaminase | 0.757 |
| 20 | 5740430 | 5750933 | serine incorporator 3 precursor | 0.757 |
| 20 | 5748294 | 5755903 | alpha-tocopherol transfer protein-like | 0.757 |
| 20 | 5763173 | 5777781 | hepatocyte nuclear factor 4-alpha | 0.757 |
| 20 | 5792281 | 5795875 | R3H domain containing-like | 0.757 |
| 20 | 5837979 | 5845069 | uncharacterized protein C20orf111 homolog | 0.757 |
| 20 | 5847927 | 5855091 | junctophilin 2 | 0.757 |
| 20 | 5895586 | 5918161 | solute carrier family 13 (sodium-dependent dicarboxylate transporter), member 3 | 0.757 |
| 20 | 5923593 | 5925045 | TP53 regulating kinase | 0.757 |
| 20 | 5928136 | 5935198 | solute carrier family 2 (facilitated glucose transporter), member 10 | 0.757 |
| 20 | 6012484 | 6104467 | eyes absent homolog 2 | 0.757 |
| 20 | 6203701 | 6257441 | nuclear receptor coactivator 3 | 0.757 |
| 20 | 6529946 | 6608286 | phosphatidylinositol-3,4,5-trisphosphate-dependent Rac exchange factor 1 | 0.757 |
| 20 | 6685428 | 6743411 | ADP-ribosylation factor guanine nucleotide-exchange factor 2 (brefeldin A-inhibited) | 0.757 |
| 20 | 6747113 | 6768662 | CSE1 chromosome segregation 1-like (yeast) | 0.757 |
| 20 | 6780571 | 6803177 | double-stranded RNA-binding protein Staufen homolog 1 | 0.757 |
| 20 | 6994464 | 7004637 | transglutaminase 3 | 0.757 |
| 20 | 7082081 | 7177518 | phosphatase and actin regulator 3 | 0.757 |
| 20 | 7219136 | 7226342 | family with sequence similarity 217, member B | 0.757 |
| 20 | 7702268 | 8001028 | cadherin-4 precursor | 0.757 |
| 20 | 7216161 | 7216973 | protein phosphatase 1, regulatory subunit 3D | 0.757 |
| 20 | 6814040 | 6814131 | Small nucleolar SNORD12/SNORD106 | 0.757 |
| 20 | 6811970 | 6812061 | Small nucleolar SNORD12/SNORD106 | 0.757 |
| 20 | 6813048 | 6813139 | Small nucleolar SNORD12/SNORD106 | 0.757 |
| 20 | 6814693 | 6814787 | Small nucleolar SNORD12/SNORD106 | 0.757 |
| 20 | 8527860 | 8554223 | solute carrier organic anion transporter family member 4A1 | 0.527 |
| 20 | 8575357 | 8620192 | neurotensin receptor type 1 | 0.527 |
| 20 | 8689083 | 8720874 | collagen alpha-3(IX) chain precursor | 0.527 |
| 20 | 8779439 | 8785907 | Glucose-induced degradation protein 8 homolog | 0.527 |
| 20 | 8837210 | 8837872 | class E basic helix-loop-helix protein 23 | 0.527 |

| | | | | |
|---|---|---|---|---|
| 20 | 10874294 | 10878191 | lysosomal protective protein precursor | 0.510 |
| 20 | 10878671 | 10881926 | phospholipid transfer protein precursor | 0.510 |
| 20 | 10887208 | 10895373 | PDX1 C-terminal inhibiting factor 1 | 0.510 |
| 21 | 3033463 | 3151837 | arginine-glutamic acid dipeptide (RE) repeats | 0.517 |
| 21 | 4679337 | 4732155 | F-actin-capping protein subunit beta isoforms 1 and 2 | 0.634 |
| 21 | 4764694 | 4774114 | Neuroblastoma suppressor of tumorigenicity 1 | 0.634 |
| 21 | 4776632 | 4780155 | 5-hydroxytryptamine receptor 6 | 0.634 |
| 21 | 4822545 | 4823109 | ring finger protein 186 | 0.634 |
| 21 | 4825556 | 4833537 | OTU domain containing 3 | 0.634 |
| 21 | 4835306 | 4836901 | group IIE secretory phospholipase A2 precursor | 0.634 |
| 21 | 4849821 | 4857034 | ATP-dependent RNA helicase DDX19B | 0.634 |
| 21 | 4890645 | 4891820 | family with sequence similarity 43, member B | 0.634 |
| 21 | 4893462 | 4897243 | cytidine deaminase | 0.634 |
| 21 | 4897971 | 4906221 | PTEN induced putative kinase 1 | 0.634 |
| 21 | 4906433 | 4908768 | Agmatinase, mitochondrial | 0.634 |
| 21 | 4909535 | 4918231 | dnaJ homolog subfamily C member 16 precursor | 0.634 |
| 21 | 4918316 | 4924583 | caspase 9, apoptosis-related cysteine peptidase | 0.634 |
| 21 | 4925271 | 4927174 | chymotrypsin C (caldecrin) | 0.634 |
| 21 | 4931027 | 4938907 | forkhead-associated (FHA) phosphopeptide binding domain 1 | 0.634 |
| 21 | 4965242 | 4973503 | kazrin, periplakin interacting protein | 0.634 |
| 21 | 5606164 | 5615523 | methylenetetrahydrofolate reductase (NAD(P)H) | 0.571 |
| 23 | 203721 | 213621 | UBX domain protein 11 | 0.560 |
| 23 | 230940 | 237139 | connector enhancer of kinase suppressor of Ras 1 | 0.560 |
| 23 | 861071 | 915914 | human immunodeficiency virus type I enhancer binding protein 3 | 0.597 |
| 24 | 1073171 | 1109460 | GIRK4; Uncharacterized protein | 0.554 |
| 24 | 6163150 | 6165998 | interleukin-18 | 0.543 |
| 24 | 6167375 | 6171382 | succinate dehydrogenase | 0.543 |
| 24 | 6176797 | 6179439 | PIH1 domain containing 2 | 0.543 |
| 25 | 1156086 | 1174496 | coatomer subunit alpha | 0.524 |
| 25 | 1174614 | 1186654 | nicastrin precursor | 0.524 |

| | | | | |
|---|---|---|---|---|
| 26 | 3399930 | 3416570 | CTTNBP2 N-terminal like | 0.518 |
| 26 | 3436365 | 3447136 | protein Wnt-2b | 0.518 |
| 26 | 3455410 | 3474542 | Suppressor of tumorigenicity 7 protein-like | 0.518 |
| 26 | 3474674 | 3483306 | F-actin-capping protein subunit alpha-1 | 0.518 |
| 26 | 3486369 | 3492328 | putative helicase MOV-10 | 0.518 |
| 26 | 3492792 | 3497763 | Rho-related GTP-binding protein RhoC | 0.518 |
| 26 | 3499167 | 3506175 | protein phosphatase, Mg2+/Mn2+ dependent, 1J | 0.518 |
| 26 | 3552668 | 3567609 | monocarboxylate transporter 1 | 0.518 |
| 26 | 3631292 | 3656668 | membrane-associated guanylate kinase, WW and PDZ domain-containing protein 3 | 0.518 |
| Z | 22034805 | 22073103 | PAP associated domain containing 4 | 0.802 |
| Z | 25582286 | 25666831 | dedicator of cytokinesis 8 | 1.000 |
| Z | 25808415 | 25864441 | doublesex- and mab-3-related transcription factor 1 | 1.000 |
| Z | 25902813 | 25905855 | doublesex and mab-3 related transcription factor 2 | 1.000 |
| Z | 26195620 | 26294557 | SWI/SNF related, matrix associated, actin dependent regulator of chromatin, subfamily a, member 2 | 1.000 |
| Z | 26415507 | 26430542 | very low density lipoprotein receptor | 1.000 |
| Z | 26488495 | 26512565 | KIAA0020 | 1.000 |
| Z | 26531633 | 26586078 | regulatory factor X, 3 (influences HLA class II expression) | 1.000 |
| Z | 26708083 | 26866140 | GLIS family zinc finger 3 | 1.000 |
| Z | 35753341 | 35806349 | retinal dehydrogenase 1 | 0.858 |
| Z | 35881518 | 35899231 | annexin A1 | 0.858 |
| Z | 39039875 | 39114050 | transducin-like enhancer of split 1 (E(sp1) homolog, Drosophila) | 0.952 |
| Z | 39574349 | 39615592 | RAS and EF-hand domain containing | 0.952 |
| Z | 39667818 | 39812156 | FERM domain containing 3 | 0.952 |
| Z | 39900490 | 39924774 | kinesin family member 27 | 0.952 |
| Z | 39936510 | 39954264 | Heterogeneous nuclear ribonucleoprotein K | 0.952 |
| Z | 39954655 | 39958510 | recQ-mediated genome instability protein 1 | 0.952 |
| Z | 38576413 | 38576570 | U1 spliceosomal RNA | 0.952 |
| Z | 42249104 | 42257660 | uncharacterized protein LOC427470 | 1.000 |
| Z | 42279475 | 42284756 | Cryptic protein | 1.000 |
| Z | 42326478 | 42396311 | leucine rich repeat containing 2 | 1.000 |

| | | | | |
|---|---|---|---|---|
| Z | 43031150 | 43106716 | Spindlin-Z | 1.000 |
| Z | 43127717 | 43128550 | nucleoredoxin-like 2 | 1.000 |
| Z | 43300148 | 43343693 | sphingosine-1-phosphate receptor 3 | 1.000 |
| Z | 43311888 | 43377895 | SHC (Src homology 2 domain containing) transforming protein 3 | 1.000 |
| Z | 43412815 | 43443779 | SECIS binding protein 2 | 1.000 |
| Z | 43446316 | 43483379 | Semaphorin-4D | 1.000 |
| Z | 43589019 | 43592114 | growth arrest and DNA damage-inducible protein GADD45 gamma | 1.000 |
| Z | 43922919 | 43975109 | Tyrosine-protein kinase SYK | 1.000 |
| Z | 44067508 | 44176409 | methylglutaconyl-CoA hydratase, mitochondrial isoform 1 | 1.000 |
| Z | 44325334 | 44479158 | tyrosine-protein kinase transmembrane receptor ROR2 precursor | 1.000 |
| Z | 44617583 | 44658144 | CDC42 small effector 2 | 1.000 |
| Z | 44758626 | 44762622 | histidine triad nucleotide-binding protein 1 | 1.000 |
| Z | 44957617 | 45108982 | chondroitin sulfate synthase 3 | 1.000 |
| Z | 45135087 | 45136473 | KIAA1024-like | 1.000 |
| Z | 45523478 | 45562834 | YTH domain containing 2 | 1.000 |
| Z | 45574534 | 45769715 | mutated in colorectal cancers | 1.000 |
| Z | 45777089 | 45804230 | decapping mRNA 2 | 1.000 |
| Z | 45811319 | 45828704 | receptor expression-enhancing protein 5 | 1.000 |
| Z | 45832341 | 45839741 | signal recognition particle 19kDa | 1.000 |
| Z | 45850647 | 45906194 | adenomatous polyposis coli | 1.000 |
| Z | 46011143 | 46145593 | erythrocyte membrane protein band 4.1 like 4A | 1.000 |
| Z | 46263610 | 46283078 | neuronal protein 3.1 | 1.000 |
| Z | 44205924 | 44207300 | nuclear factor interleukin-3-regulated protein | 1.000 |
| Z | 46144006 | 46144137 | Small nucleolar RNA SNORA13 | 1.000 |
| Z | 45526269 | 45526443 | U2 spliceosomal RNA | 1.000 |
| Z | 54549996 | 54642132 | ATP-binding cassette sub-family A member 1 | 0.825 |
| Z | 54741875 | 54804427 | solute carrier family 44 (choline transporter), member 1 | 0.825 |
| Z | 54832501 | 54860934 | fibronectin type III and SPRY domain containing 1-like | 0.825 |
| Z | 54866069 | 54882250 | fukutin | 0.825 |
| Z | 57916145 | 58180255 | Protein FAM172A | 0.885 |

| | | | | |
|---|---|---|---|---|
| Z | 62344745 | 62440821 | Hyaluronan and proteoglycan link protein 1 | 0.794 |
| Z | 63574950 | 63597283 | creatine kinase S-type, mitochondrial | 0.926 |
| Z | 63601953 | 63729018 | Ras protein-specific guanine nucleotide-releasing factor 2 | 0.926 |
| Z | 63759855 | 63876936 | mutS homolog 3 (E. coli) | 0.926 |
| Z | 63876645 | 63893410 | dihydrofolate reductase | 0.926 |
| Z | 63984743 | 63988783 | adenylate kinase isoenzyme 6 | 0.926 |
| Z | 63988631 | 64006407 | cell cycle checkpoint protein RAD17 | 0.926 |
| Z | 65644355 | 65663759 | A-kinase anchor protein 2 | 0.869 |
| Z | 65673926 | 65679018 | solute carrier family 46, member 2 | 0.869 |
| Z | 65685568 | 65734943 | sorting nexin family member 30 | 0.869 |
| Z | 65744965 | 65755449 | SOSS complex subunit C | 0.869 |
