# Supplementary material for: Population Genomic Analyses Based on 1 Million SNPs in Commercial Egg Layers
Source: PLoS One. 2014 Apr 16;9(4):e94509. doi: 10.1371/journal.pone.0094509 (PMC3989219; doi:10.1371/journal.pone.0094509)
Supplement: Table S5 — List of pathways and gene ontologies in lower 1% distribution of the comparison of commercial layers and out-group. (PDF) [file pone.0094509.s005.pdf]

Table S5. List of pathways and gene ontologies in lower 1% distribution of the comparison of commercial layers and out-group.

Description	# Genes anotated	Genes of pathways (%)*	P-Value
thiolester hydrolase activity	10	1.44	0.001
potassium channel	6	0.86	0.002
potassium ion binding	7	1.01	0.003
voltage-gated channel	6	0.86	0.003
potassium transport	7	1.01	0.004
ubiquitin thiolesterase activity	8	1.15	0.004
alkali metal ion binding	7	1.01	0.007
glutamine family amino acid metabolic process	6	0.86	0.007
potassium	7	1.01	0.008
macromolecule catabolic process	19	2.74	0.010
regulation of multicellular organism growth	6	0.86	0.010
enzyme linked receptor protein signaling pathway	15	2.16	0.011
behavior	14	2.02	0.012
plasma membrane part	36	5.19	0.012
Golgi membrane	10	1.44	0.013
plasma membrane	52	7.49	0.013
GTPase activation	5	0.72	0.014
Posttranslational modification, protein turnover, chaperones	10	1.44	0.015
RNA splicing	7	1.01	0.019
voltage-gated cation channel activity	10	1.44	0.019
transmembrane receptor protein tyrosine kinase signaling pathway	11	1.59	0.021
modification-dependent macromolecule catabolic process	13	1.87	0.022
modification-dependent protein catabolic process	13	1.87	0.022
mRNA metabolic process	9	1.3	0.022
protein catabolic process	15	2.16	0.023

proteolysis involved in cellular protein catabolic process	14	2.02	0.023
cellular protein catabolic process	14	2.02	0.025
regulation of growth	11	1.59	0.027
activator	13	1.87	0.030
cellular macromolecule catabolic process	16	2.31	0.030
regulation of lipid metabolic process	5	0.72	0.030
voltage-gated potassium channel activity	8	1.15	0.030
proteolysis	30	4.32	0.035
apical part of cell	6	0.86	0.037
transcription activator activity	10	1.44	0.037
ubiquitin-dependent protein catabolic process	9	1.3	0.037
Golgi apparatus part	11	1.59	0.040
positive regulation of multicellular organismal process	9	1.3	0.043
positive regulation of transferase activity	8	1.15	0.043
gated channel activity	15	2.16	0.046
in utero embryonic development	9	1.3	0.046
RNA polymerase II transcription factor activity	7	1.01	0.048

*Percentage of the genes of the pathway which were among the annotated genes.
