# Supplementary material for: Population Genomic Analyses Based on 1 Million SNPs in Commercial Egg Layers
Source: PLoS One. 2014 Apr 16;9(4):e94509. doi: 10.1371/journal.pone.0094509 (PMC3989219; doi:10.1371/journal.pone.0094509)
Supplement: Table S6 — List of pathways and gene ontologies in upper 1% distribution of the comparison of commercial layers and out-group. (PDF) [file pone.0094509.s006.pdf]

Table S6. List of pathways and gene ontologies in upper 1% distribution of the comparison of commercial layers and out-group.

**Description**	**# Genes anotated**	**Genes of pathways (%)***	**P-Value**
regulation of cell proliferation	27	3.92	0.000
proteoglycan	7	1.02	0.001
regulation of apoptosis	24	3.49	0.001
cell cycle process	15	2.18	0.002
cytoskeleton organization	16	2.33	0.002
cytosol	17	2.47	0.002
regulation of cell death	24	3.49	0.002
regulation of programmed cell death	24	3.49	0.002
carbohydrate biosynthetic process	8	1.16	0.003
cell cycle	18	2.62	0.003
positive regulation of cell proliferation	17	2.47	0.003
positive regulation of cell communication	13	1.89	0.004
ribonucleoprotein complex assembly	5	0.73	0.004
negative regulation of transcription from RNA polymerase II promoter	10	1.45	0.005
neurotransmitter binding	10	1.45	0.005
neurotransmitter receptor activity	10	1.45	0.005
phosphoprotein phosphatase activity	12	1.74	0.005
regulation of transcription from RNA polymerase II promoter	22	3.20	0.005
ligand-gated channel activity	11	1.60	0.008
ligand-gated ion channel activity	11	1.60	0.008
positive regulation of apoptosis	12	1.74	0.008
positive regulation of programmed cell death	12	1.74	0.008
positive regulation of signal transduction	12	1.74	0.008
ionic channel	12	1.74	0.009
positive regulation of cell death	12	1.74	0.009

cytoplasmic membrane-bounded vesicle	12	1.74	0.010
membrane-bounded vesicle	12	1.74	0.013
protein amino acid dephosphorylation	10	1.45	0.013
tube development	13	1.89	0.013
dephosphorylation	11	1.60	0.014
mitotic cell cycle	9	1.31	0.014
cysteine-type peptidase activity	8	1.16	0.015
phosphate metabolic process	42	6.10	0.015
phosphorus metabolic process	42	6.10	0.015
protein tyrosine phosphatase activity	9	1.31	0.015
proteinaceous extracellular matrix	16	2.33	0.015
compositionally biased region:Cys-rich	6	0.87	0.016
compositionally biased region:Ser-rich	7	1.02	0.016
regulation of growth	12	1.74	0.016
ion channel activity	20	2.91	0.017
channel activity	20	2.91	0.018
passive transmembrane transporter activity	20	2.91	0.018
substrate specific channel activity	20	2.91	0.018
DNA damage response, signal transduction	5	0.73	0.019
regulation of nervous system development	9	1.31	0.019
cytoplasmic vesicle	12	1.74	0.020
synapse	12	1.74	0.021
extracellular ligand-gated ion channel activity	8	1.16	0.022
negative regulation of transcription, DNA-dependent	11	1.60	0.022
extracellular matrix	16	2.33	0.023
cell cycle phase	10	1.45	0.024
positive regulation of transcription from RNA polymerase II promoter	15	2.18	0.024
regulation of neuron differentiation	7	1.02	0.024
tube morphogenesis	9	1.31	0.024
hemopoietic or lymphoid organ development	12	1.74	0.025

hydrolase	28	4.07	0.025
cysteine-type endopeptidase activity	6	0.87	0.027
identical protein binding	15	2.18	0.027
negative regulation of RNA metabolic process	11	1.60	0.027
vesicle	12	1.74	0.028
hemopoiesis	11	1.60	0.029
positive regulation of transcription, DNA-dependent	16	2.33	0.029
chloride channel activity	5	0.73	0.030
ion transport	14	2.03	0.030
positive regulation of nucleobase, nucleoside, nucleotide and nucleic acid metabolic process	20	2.91	0.030
positive regulation of DNA metabolic process	5	0.73	0.031
pattern binding	7	1.02	0.032
polysaccharide binding	7	1.02	0.032
positive regulation of RNA metabolic process	16	2.33	0.032
neurotransmitter receptor	6	0.87	0.033
glycosaminoglycan binding	6	0.87	0.034
immune system development	12	1.74	0.034
cellular carbohydrate biosynthetic process	5	0.73	0.036
calcium ion transport	8	1.16	0.037
negative regulation of multicellular organismal process	7	1.02	0.037
nuclear envelope	7	1.02	0.037
cell surface	11	1.60	0.038
positive regulation of nitrogen compound metabolic process	20	2.91	0.038
small GTPase regulator activity	12	1.74	0.038
induction of apoptosis	7	1.02	0.040
induction of programmed cell death	7	1.02	0.040
negative regulation of cell proliferation	10	1.45	0.040
repeat:LRR 3	5	0.73	0.040
placenta development	5	0.73	0.041
protein dimerization activity	15	2.18	0.041

actin filament-based process	9	1.31	0.042
myeloid cell differentiation	6	0.87	0.042
cell junction	13	1.89	0.044
phosphatase activity	13	1.89	0.044
synapse part	9	1.31	0.045
organelle envelope	16	2.33	0.046
postsynaptic cell membrane	7	1.02	0.048
synapse	8	1.16	0.050

*Percentage of the genes of the pathway which were among the annotated genes.
