# Supplementary material for: Population Genomic Analyses Based on 1 Million SNPs in Commercial Egg Layers
Source: PLoS One. 2014 Apr 16;9(4):e94509. doi: 10.1371/journal.pone.0094509 (PMC3989219; doi:10.1371/journal.pone.0094509)
Supplement: Table S7 — List of pathways and gene ontologies in lower 1% FST distribution in comparison of brown layers and white layers. (PDF) [file pone.0094509.s007.pdf]

Table S7. List of pathways and gene ontologies in lower 1% F_{ST} distribution in comparison of brown layers and white layers.

**Description**	**# Genes anotated**	**Genes of pathways (%)***	**P-Value**
plasma membrane	50	8.80	0.000
plasma membrane part	36	6.34	0.000
cell junction	15	2.64	0.001
anti-apoptosis	8	1.41	0.003
nucleotide binding	72	12.68	0.004
RNA binding	18	3.17	0.006
GTPase activation	5	0.88	0.007
sensory organ development	12	2.11	0.007
enzyme linked receptor protein signaling pathway	14	2.46	0.009
behavior	13	2.29	0.010
cellular amino acid catabolic process	5	0.88	0.012
regulation of growth	11	1.94	0.012
sensory perception of sound	5	0.88	0.012
amine catabolic process	5	0.88	0.017
neuron differentiation	13	2.29	0.017
sensory perception of mechanical stimulus	5	0.88	0.017
negative regulation of transcription	12	2.11	0.019
regulation of cell proliferation	19	3.35	0.020
ear development	7	1.23	0.022
cell motility	11	1.94	0.023
localization of cell	11	1.94	0.023
cell motion	13	2.29	0.024
N-Glycan biosynthesis	6	1.06	0.024
transcription activator activity	9	1.58	0.028
positive regulation of transcription from RNA polymerase II promoter	13	2.29	0.029
regulation of multicellular organism growth	5	0.88	0.030
negative regulation of gene expression	12	2.11	0.039

Notch signaling pathway	6	1.06	0.040
cell migration	10	1.76	0.041
negative regulation of cellular biosynthetic process	13	2.29	0.042
carboxylic acid catabolic process	5	0.88	0.043
organic acid catabolic process	5	0.88	0.043
negative regulation of nucleobase, nucleoside, nucleotide and nucleic acid metabolic process	12	2.11	0.047
positive regulation of cell proliferation	12	2.11	0.047
negative regulation of biosynthetic process	13	2.29	0.048
basolateral plasma membrane	6	1.06	0.049

*Percentage of the genes of the pathway which were among the annotated genes.
