# Supplementary material for: Population Genomic Analyses Based on 1 Million SNPs in Commercial Egg Layers
Source: PLoS One. 2014 Apr 16;9(4):e94509. doi: 10.1371/journal.pone.0094509 (PMC3989219; doi:10.1371/journal.pone.0094509)
Supplement: Table S8 — List of pathways and gene ontologies in upper1% FST distribution in comparison of brown layers and white layers. (PDF) [file pone.0094509.s008.pdf]

Table S8. List of pathways and gene ontologies in upper1% F_{ST} distribution in comparison of brown layers and white layers.

Description	# Genes anotated	Genes of pathways (%)*	P-Value
actin binding	15	2.6	0.000
actin cytoskeleton organization	12	2.1	0.000
actin filament-based process	13	2.2	0.000
adenyl nucleotide binding	65	11.2	0.000
adenyl ribonucleotide binding	63	10.9	0.000
ATP binding	62	10.7	0.000
atp-binding	34	5.9	0.001
cytoskeletal protein binding	18	3.1	0.001
cytoskeleton organization	15	2.6	0.001
cellular response to stress	18	3.1	0.002
erythrocyte homeostasis	6	1.0	0.002
nuclear envelope	9	1.6	0.002
phosphate metabolic process	40	6.9	0.002
phosphorus metabolic process	40	6.9	0.002
protein amino acid phosphorylation	32	5.5	0.002
protein kinase activity	31	5.4	0.002
purine ribonucleotide binding	69	11.9	0.002
ribonucleotide binding	69	11.9	0.002
envelope	19	3.3	0.003
hemopoiesis	12	2.1	0.003
organelle envelope	19	3.3	0.003
phosphorylation	35	6.1	0.003
purine nucleotide binding	71	12.3	0.003
homeostatic process	21	3.6	0.004
immune system development	13	2.2	0.004
nuclear chromosome part	8	1.4	0.004
protein tyrosine kinase activity	13	2.2	0.004

response to oxygen levels	6	1.0	0.004
transferase	30	5.2	0.004
anatomical structure homeostasis	7	1.2	0.007
hemopoietic or lymphoid organ development	12	2.1	0.008
nuclear chromosome	8	1.4	0.008
erythrocyte differentiation	5	0.9	0.009
basolateral plasma membrane	8	1.4	0.010
glycoprotein	38	6.6	0.012
glycosylation site:N-linked (GlcNAc...)	37	6.4	0.012
Natural killer cell mediated cytotoxicity	9	1.6	0.012
nucleotide binding	78	13.5	0.012
regulation of phosphorylation	13	2.2	0.012
nucleotide phosphate-binding region:ATP	16	2.8	0.013
regulation of cell adhesion	7	1.2	0.013
actin-binding	9	1.6	0.014
kinase	19	3.3	0.015
regulation of phosphate metabolic process	13	2.2	0.016
regulation of phosphorus metabolic process	13	2.2	0.016
intracellular signaling cascade	28	4.8	0.018
response to hypoxia	5	0.9	0.018
calcium	18	3.1	0.019
lipid binding	13	2.2	0.019
positive regulation of signal transduction	10	1.7	0.019
topological domain:Cytoplasmic	28	4.8	0.019
protein complex binding	5	0.9	0.020
cellular homeostasis	13	2.2	0.021
myeloid cell differentiation	6	1.0	0.022
cell membrane	22	3.8	0.023
locomotory behavior	9	1.6	0.023
positive regulation of cell communication	10	1.7	0.024

behavior	12	2.1	0.027
DNA metabolic process	18	3.1	0.029
transferase activity, transferring sulfur-containing groups	6	1.0	0.030
B cell activation	5	0.9	0.031
calcium ion binding	29	5.0	0.033
cell adhesion	10	1.7	0.033
reproductive cellular process	7	1.2	0.034
phosphoprotein	26	4.5	0.035
post-embryonic development	5	0.9	0.035
cytosol	13	2.2	0.037
response to DNA damage stimulus	12	2.1	0.039
enzyme binding	10	1.7	0.040
regulation of cell morphogenesis	5	0.9	0.040
homeostasis of number of cells	6	1.0	0.041
membrane fraction	11	1.9	0.043
regulation of cell proliferation	18	3.1	0.043
cell migration	10	1.7	0.044
topological domain:Extracellular	22	3.8	0.044

*Percentage of the genes of the pathway which were among the annotated genes.
